# Low Nitrogen Fertilization Adapts Rice Root Microbiome to Low Nutrient Environment by Changing Biogeochemical Functions

**DOI:** 10.1264/jsme2.ME13110

**Published:** 2014-01-24

**Authors:** Seishi Ikeda, Kazuhiro Sasaki, Takashi Okubo, Akifumu Yamashita, Kimihiro Terasawa, Zhihua Bao, Dongyan Liu, Takeshi Watanabe, Jun Murase, Susumu Asakawa, Shima Eda, Hisayuki Mitsui, Tadashi Sato, Kiwamu Minamisawa

**Affiliations:** 1 Graduate School of Life Sciences, Tohoku University, Katahira, Aoba-ku, Sendai, Miyagi 980–8577, Japan; 2 Memuro Research Station, National Agricultural Research Center for Hokkaido Region, Shinsei, Memuro-cho, Kasaigun, Hokkaido 082–0081, Japan; 3 Soil Biology and Chemistry, Graduate School of Bioagricultural Sciences, Nagoya University, Chikusa, Nagoya 464–8601, Japan

**Keywords:** rice paddy field, nitrogen fertilizer, methane cycle, metagenome analysis, root microbiome

## Abstract

Reduced fertilizer usage is one of the objectives of field management in the pursuit of sustainable agriculture. Here, we report on shifts of bacterial communities in paddy rice ecosystems with low (LN), standard (SN), and high (HN) levels of N fertilizer application (0, 30, and 300 kg N ha^−1^, respectively). The LN field had received no N fertilizer for 5 years prior to the experiment. The LN and HN plants showed a 50% decrease and a 60% increase in biomass compared with the SN plant biomass, respectively. Analyses of 16S rRNA genes suggested shifts of bacterial communities between the LN and SN root microbiomes, which were statistically confirmed by metagenome analyses. The relative abundances of *Burkholderia*, *Bradyrhizobium* and *Methylosinus* were significantly increased in root microbiome of the LN field relative to the SN field. Conversely, the abundance of methanogenic archaea was reduced in the LN field relative to the SN field. The functional genes for methane oxidation (*pmo* and *mmo*) and plant association (*acdS* and *iaaMH*) were significantly abundant in the LN root microbiome. Quantitative PCR of *pmoA*/*mcrA* genes and a ^13^C methane experiment provided evidence of more active methane oxidation in the rice roots of the LN field. In addition, functional genes for the metabolism of N, S, Fe, and aromatic compounds were more abundant in the LN root microbiome. These results suggest that low-N-fertilizer management is an important factor in shaping the microbial community structure containing key microbes for plant associations and biogeochemical processes in paddy rice ecosystems.

Rice is one of the most important cereal crops in the world and is grown mainly in flooded paddy fields with the implicit or explicit objective of sustainably maintaining soil fertility (25). Biogeochemical processes including the emission of methane, a greenhouse gas, occur actively in paddy rice ecosystems, and the rhizosphere in a paddy field is considered to be a hot spot for the redox transformation of a variety of inorganic substances, including Fe, S and N (21, 25, 28). These processes are principally mediated by microbial communities. Rice roots play a role in that they supply O_2_ and root organic matter (exudates, dead roots) to the rhizosphere. Microbial community dynamics and structure-function relationships have been intensively studied for methanogens and methanotrophs in flooded rice paddies (7, 10, 21, 25, 33). However, the complex systems of key microbial players and their functions in these biogeochemical processes have been not fully elucidated in paddy rice fields.

Nitrogen is the most important mineral nutrient for crop production, and an adequate supply of N fertilizer is essential for sustaining high yields. However, the production of synthetic N fertilizers requires large amounts of fossil fuel, and their heavy use results in environmental problems. Human alteration of N cycles is therefore held to harm human and ecosystem health (11). Nitrogen fertilization levels are generally thought to affect methane emission from rice fields, although this topic is currently under debate (1, 5, 44, 48). To attain more sustainable agriculture, knowledge-based optimum N fertilization has been proposed in agricultural ecosystems, including paddy fields (22).

Massive parallel sequencing technologies enable us to characterize more comprehensive, whole structures of microbial communities in diverse environments and therefore allow us to assess changes in community composition by environmental factors (29). A technique for bacterial cell enrichment from plant tissues (18) has provided a gateway for the metagenome analysis of plant-associated bacteria (20). By combining these methodologies, we are now able to assess the functional diversities of plant-associated microbiomes by metagenomic analysis, as shown for a diverse range of environments (29).

Here we report global shifts in microbial communities in paddy rice ecosystems under different N fertilizer regimes by analyses of bacterial 16S rRNA genes and community metagenomics. The results of microbial communities revealed that low N fertilization management has considerable impact on the biogeochemical processes and plant nutrient uptake of a wide variety of substances in a paddy field ecosystem.

## Materials and Methods

### Plant materials

Rice (*Oryza sativa* L. cv. Nipponbare) seeds were germinated on filter paper (Advantec-Toyo, Tokyo, Japan) at 30°C. After 2 days, the germinated seeds were sown in commercial soil (Mitsui-Toatsu No. 3, Tokyo, Japan) in a 60×30 cm cell tray (1.5 cm cell diameter, 3 cm depth) and grown in a greenhouse under natural light for 4 weeks. A total of 300 seedlings, 100 for each treatment, were transplanted on 20 May 2009 into planting hills arranged in a square pattern of 10×10 plants at 30 cm spacing in experimental paddy fields of Kashimadai Experimental Station (Tohoku University; (38°27″37′ N and 141°5″33′ E). Rice plants were cultivated under waterlogged conditions (water depth, 30 cm). Shoot length, tiller number, and shoot fresh weight were measured at the booting stage (90 days after transplanting).

### Fertilization

Rice seedlings were grown in three neighboring fields (approximately 400 m^2^ each) at different levels of N fertilization (standard N, SN; low N, LN; and high N, HN) (Fig. S1). From 2004 to 2009, the SN paddy field was fertilized with N, P, and K (Temairazu 666; Co-op Chemical, Tokyo, Japan) at a rate of 30 kg ha^−1^ each (expressed as N, P_2_O_5_ and K_2_O). In the LN field, only P and K were applied using P–K fertilizer No. 46 (Co-op Chemical) at 30 kg ha^−1^ each (expressed as P_2_O_5_ and K_2_O). The LN paddy field had been managed for rice cultivation using identical field management methods as for the SN field except for the withholding of N fertilizer from 2004 on. In the HN paddy field, for the 2009 growing season, N, P, and K were applied at 30 kg ha^−1^ each (expressed as N, P_2_O_5_ and K_2_O) as basal fertilizer similar to the SN field. During cultivation, 30 or 60 kg ha^−1^ of ammonium sulfate (Ube Agri-Materials, Tokyo, Japan) was additionally applied every 2 weeks until 270 kg N ha^−1^ had been applied. Thus, the HN field was totally subjected to 300 kg ha^−1^ as N fertilizer.

### DNA preparation

Rice plants and bulk soils were sampled from the field at the booting stage on 26 August 2009. The rice plants were carefully washed with tap water until no soil particles remained. Three sets of the composite samples including at least three rice plants were independently prepared for the roots or shoots (approximately 100 g each) in each treatment, and then stored at −80°C until they were used. The composite samples of roots or shoots were homogenized without surface sterilization to prepare shoot- and root-associated bacterial cells containing both epiphytes and endophytes. The bacterial cells were independently extracted and purified using a method for the enrichment of bacterial cells from each set of the plant composite samples (18). DNase treatment was added to the procedure to remove plant DNA. After the final bacterial cell suspension was incubated in the presence of recombinant DNase I (Takara, Otsu, Japan) for 20 min at 37°C, according to the instructions of the manufacturer, the reaction was terminated by the addition of 0.5 M EDTA at a final concentration of 25 mM. Bulk soils were sampled similar to the rice plants (0–15 cm depth and triplicates), and then stored at −80°C until they were used. Total DNA of the enriched bacterial cells and bulk soils was extracted by the method of Ikeda *et al.* (17, 20). The quality and quantity of DNA were assessed spectrophotometrically from the absorbance at a wavelength of 260 nm (A_260_) and the A_260_/A_230_ and A_260_/A_280_ ratios.

### Sequence analyses

The construction of PCR clone libraries of rice shoot and roots grown in the LN, SN and HN fields for 16S rRNA genes, their sequencing by the Sanger method, statistics, and phylogenetic analysis were carried out as described previously (17–20). Briefly, 50 ng total bacterial DNA was used as a template in a final reaction volume of 60 μL, including 90 pmol of each primer and 9 U of *Ex Taq* DNA polymerase (Takara Bio, Otsu, Japan). The universal primers 27F (50-AGAGTTTGATCMTGGCTCAG-30) and 1525R (50-AAGGAGGTGWTCCARCC-30) were used. A partial sequence of the 16S rRNA gene was obtained using the 27F primer. Then the 16S rRNA gene (corresponding to bp 109–665 of the *Escherichia coli* 16S rRNA gene) was used for sequence analyses. Only for the Sanger method, three replicated samples were combined for each treatment before sequencing.

The DNAs of the enriched bacterial cells from the LN and SN root microbiomes were shotgun-sequenced by means of a 454 GS FLX Titanium pyrosequencer with three replications for statistical analysis. The metagenomic reads in each sample were taxonomically assigned according to the best-hit pairs in BLASTX analysis against the GenBank NR database with an E-value threshold of 10^−10^. Gene IDs for all hits were collected and phylogenetically placed with an in-house script. For pyrosequencing of 16S rRNA genes of soil- and root-associated bacteria, the 16S rRNA genes were amplified using Bac-27F and MID-518R primers and sequenced on the 454 GS FLX Titanium pyrosequencer. Taxonomy was assigned using the Ribosomal Database Project (RDP) MultiClassifier with a minimum support threshold of 50%. The Fast UniFrac algorithm (13) was used to determine the degree of bacterial diversity between the communities. More detailed information on sequence analyses is given in the Supplementary Materials.

### Prediction of functional gene categories from metagenome sequences

The metabolic potentials of each microbiome were also assessed by BLASTX search (E-value <10^−5^) of the SEED database on the MG-RAST server (35). The metagenomic reads were uploaded to the MG-RAST Web site (http://metagenomics.nmpdr.org/). After we retrieved the target functional gene categories for N_2_ fixation, methane cycling, and plant hormones, we selected subcategories whose abundance was >2× in the LN roots that in the SN roots (*P* <0.01). More detailed information on functional gene analysis is given in the Supplementary Materials.

### Quantitative PCR

Quantitative PCR was carried out with a Thermal Cycler Dice Real Time System (TaKaRa, Shiga, Japan) with primers mcrA-f and mcrA-r (30) for the *mcrA* gene (47), or A189f (15) and mb661r (9) for the *pmoA* gene (26). The PCR conditions were 45 cycles of denaturation at 95°C for 40 s, annealing at 55°C for 30 s, and extension at 72°C for 60 s for *mcrA*; and 40 cycles of denaturation at 95°C for 30 s, annealing at 65.5°C for 20 s, and extension at 72°C for 40 s for *pmoA*. Clones of *pmoA* genes derived from *Methylosinus trichosporium* strain OB3b (acc. no. U31650) and *Methylomonas* sp. strain Fw12E-Y (acc. no. AB538965) were used as the standard reference for the quantification of *pmoA* genes.

### Feeding of ^13^C-labeled methane

Rice roots (*Oryza sativa* cv. Nipponbare) were sampled from the LN and SN paddy fields and then washed with tap water. The root systems were immediately introduced into the bag assembly (approximately 1 liter) with a sampling port (2). The gas phase in the assembly was replaced by 10% (v/v) ^13^C-labeled methane (99.9 atom%; Shoko, Tokyo, Japan) in the air. Negative control was conducted with the same assembly without ^13^C-labeled methane. After static incubation of the root systems in the assembly at 25°C for 26 h in the dark, they were dried at 80°C for 3 days and then powdered in a blender (HBF400; Hamilton Beach, Glen Allen, VA, USA). To estimate the amount of ^13^C assimilated by methanotrophs in the root systems, the ^13^C and total carbon contents of the powdered rice root tissues were determined by a mass spectrometer (EA1110-DELTA^plus^ Advantage ConFlo III System; Thermo Fisher Scientific, Bremen, Germany).

### Soil analysis

Bulk soil of paddy fields under low N (LN) and standard N (SN) conditions was air-dried, and sieved through a 2-mm-mesh sieve. Total N, NH_4_^+^, and NO_3_^−^ were determined as described (16). Organic substance content and cation exchange capacity (CEC) were determined by Tokachi Federation Cooperatives (Tokachi, Obihiro, Japan).

### Nucleotide sequence accession number

Nucleotide sequences of 16S rRNA bacterial genes have been deposited in the DDBJ database as follows: LN shoots (accession numbers AB579015–AB579146), SN shoots (AB579147– AB579297), HN shoots (AB579298–AB579456), LN roots (AB579457–AB579568), SN roots (AB579569–AB579659), and HN roots (AB579660–AB579765). Metagenomic and 16S rRNA sequences obtained by the 454 GS FLX Titanium pyrosequencer have been deposited in the NCBI database under project ID 61421 (DDBJ Sequence Read Archive, DRA000321).

## Results

### Rice growth

**Fig. 1:**
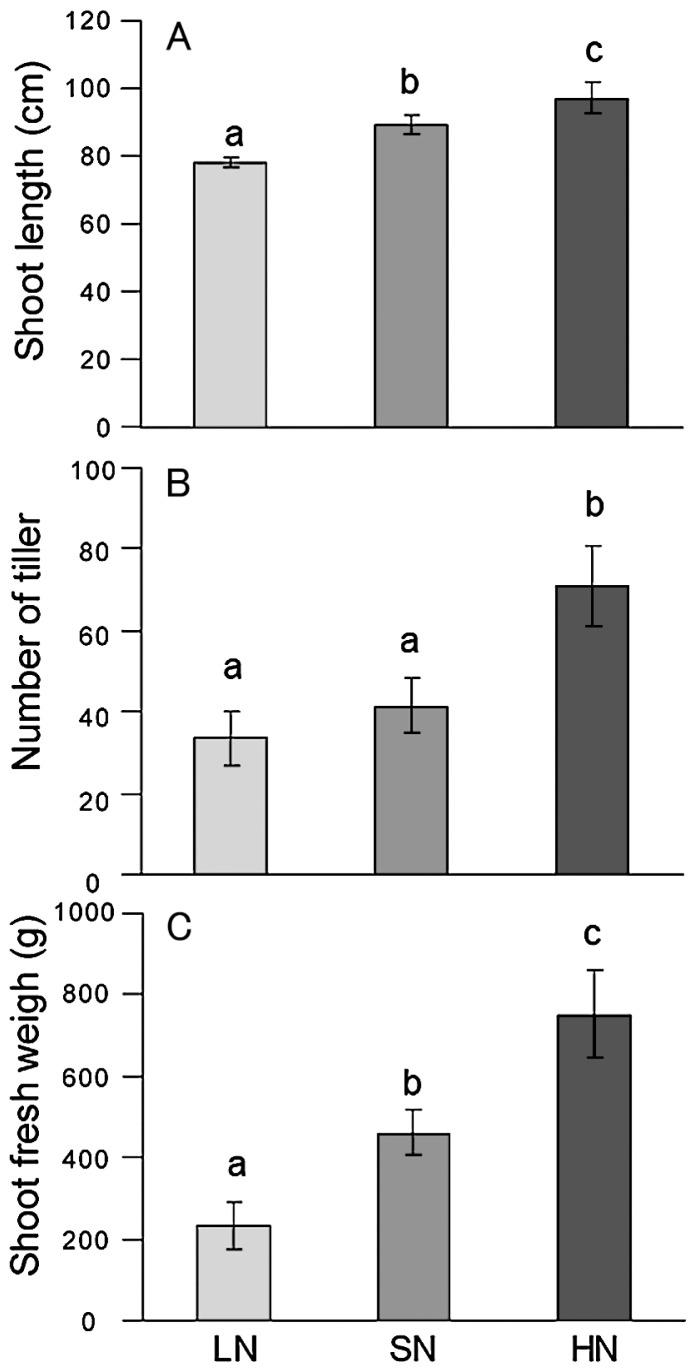
Growth parameters of rice grown under low (LN), standard (SN), and high (HN) N fertilization regimes. (A) Shoot length, (B) number of tillers, and (C) fresh weight of shoots. Data show the mean ± SEM. Whole plants were destructively sampled at the booting stage on 27 August 2009 and weighed. Bars bearing the same letter (a–c) within a panel are not significantly different according to Tukey’s test for pairwise mean comparison at α = 0.05.

Rice plants were grown in paddy fields with three levels of N fertilizer application—low (LN), standard N (SN) and high (HN)—with application rates of 0, 30, and 300 kg N ha^−1^, respectively. Prior to the experiments, the LN field had not received N fertilizer for 5 years (Fig. S1). Rice growth positively responded to the amount of N fertilizer application (Fig. 1, Fig. S1). The shoot length, and biomass of the LN plants significantly decreased as compared with the SN plants (Fig. 1). On the other hand, the growth indexes (shoot length, tiller number and shoot fresh weight) of HN plants significantly increased as compared with the SN plants (Fig. 1). Based on the SN plant biomass (shoot fresh weight), LN and HN plants showed a 50% decrease and a 60% increase in plant biomass, respectively. Thus, we examined how the rice-associated bacterial communities responded to N fertilizer levels.

### Phylogenetic diversity by clone library analysis

The enriched bacterial cells from rice shoots and roots were subjected to clone library analyses of bacterial 16S rRNA genes. The root microbiome of rice plants from the LN fields had the lowest number of operational taxonomic units (OTUs) and the lowest values of the Shannon and Simpson diversity indexes among all libraries (Table 1). For the shoot microbiomes, the numbers of OTUs and diversity indexes among libraries were similar regardless of the N fertilization level.

**Fig. 2:**
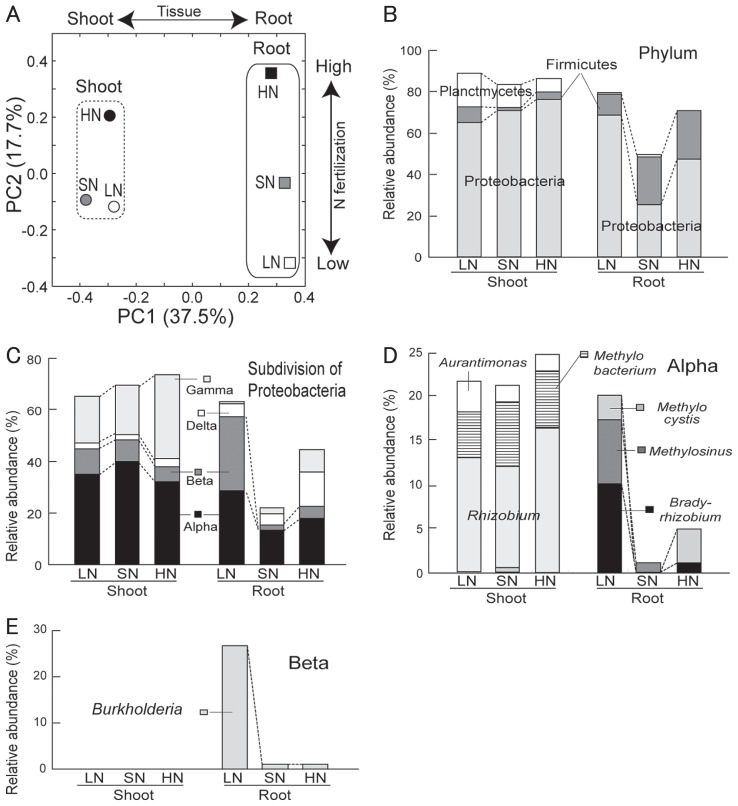
Principal coordinate analysis (PCoA) and phylogenetic compositions of 16S ribosomal RNA gene clone libraries of rice shoot- and root-associated bacterial communities under low (LN), standard (SN), and high (HN) N fertilization. Panel A, PCoA ordination was constructed using Unifrac distances. (B–E) Relative abundances of the phyla (B), subdivision of *Proteobacteria* (C), and the major genera of *Alphaproteobacteria* (D) and *Betaproteobacteria* (E). Alpha, Beta, Gamma, and Delta indicate *Alpha*-, *Beta*-, *Gamma*-, and *Delta*-*proteobacteria*, respectively.

In principal coordinate analysis (PCoA), shoot and root microbiomes were differentiated along PC1 (37.5%; Fig. 2A), whereas the effects of the N fertilization level were explained as unidirectional shifts in community structure along PC2 (17.7%) for both microbiomes (Fig. 2A). In particular, the root microbiomes appeared to have distinct community structures in response to different N fertilizer levels (Fig. 2A).

The assessment of phylogenetic composition using RDP Classifier revealed that *Proteobacteria* were the dominant taxa across all libraries at phylum level (Fig. 2B). In the root microbiomes, the second major phylum was *Firmicutes*, whereas in the shoot microbiomes, it was *Planctomycetes* (Fig. 2B). In *Proteobacteria*, *Alpha*- and *Gammaproteobacteria* were stably dominant in the shoots, whereas the abundance of *Alpha*-, *Beta*- and *Delta*-*proteobacteria* in the roots responded to N levels (Fig. 2C). In particular, *Betaproteobacteria* were markedly abundant in the LN root microbiomes (Fig. 2C). At genus level, the relative abundances of *Bradyrhizobium* and *Methylosinus* (Fig. 2D) and *Burkholderia* (Fig. 2E) were increased exclusively in the LN root microbiome. In the shoot microbiomes, *Rhizobium*, *Methylobacterium* and *Aurantimonas* were major genera, but their abundance did not respond to the N fertilizer level (Fig. 2D).

**Fig. 3:**
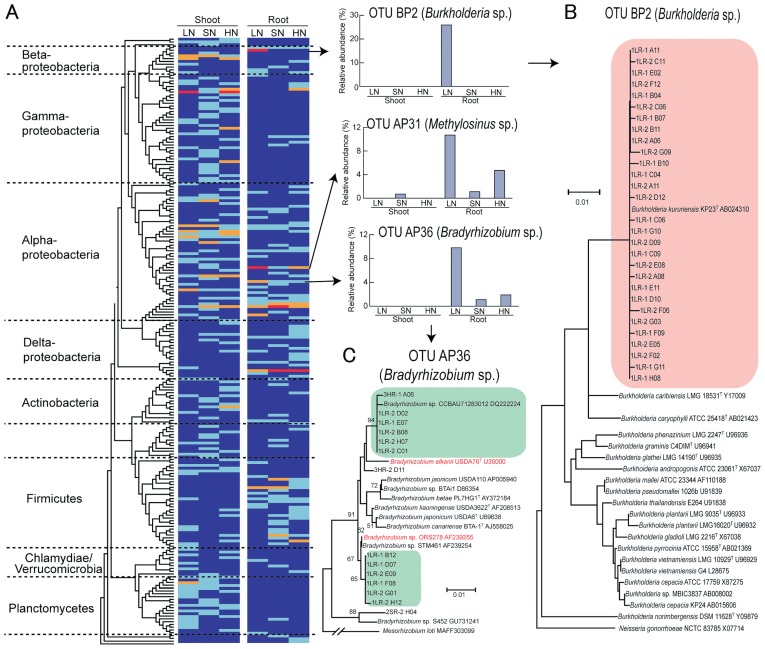
Phylogenetic distribution of operational taxonomic units (OTUs) of the clone libraries of 16S ribosomal RNA genes for rice shoot- and root-associated bacteria under low (LN), standard (SN), and high (HN) N fertilization. (A) The relative abundances of OTUs in each library are indicated in the heat map by different colors: sky blue (<3%), orange (3%–10%) and red (>10%), against a background of dark blue (0%). Bar graphs indicate the abundance of the three OTUs that noticeably fluctuated with N levels. (B) The phylogenetic tree of OTU BP2 is represented by *Burkholderia* sp. All sequences were grouped into a cluster (pink shading) including *B. kururiensis* KP23^T^. (C) The phylogenetic tree of OTU AP36 is represented by *Bradyrhizobium* sp. Most sequences fell into two clusters (green shading) that are phylogenetically close to *B. elkanii* and photosynthetic *Bradyrhizobium* sp. ORS278. The scale represents 0.01 substitutions per site. The numbers at the nodes are the proportions of 1000 bootstrap resamplings, and values of <50% are not shown. The phylogenetic tree for OTU AP31 represented by *Methylocystis* sp. is shown in Fig. S2.

Clustering analysis (Fig. 3A) allowed the identification of OTUs responsible for the community shifts in LN fertilization at species level. The results showed that OTUs BP2 (*Burkholderia* sp.), AP31 (*Methylosinus* sp.), and AP36 (*Bradyrhizobium* sp.) were especially abundant in the LN roots (Fig. 3A). When phylogenetic trees were constructed using clone sequences belonging to each OTU, all clones of OTU BP2 were grouped into a tight and distinct cluster including *Burkholderia kururiensis* KP23^T^ (50) (Fig. 3B). Most of the clones in OTU AP36 fell into two major clusters that were phylogenetically close to photosynthetic stem-nodulating *Bradyrhizobium* sp. ORS278 (12) and *Bradyrhizobium elkanii* USDA76^T^ (42) (Fig. 3C). Half of the clones in OTU AP31 were grouped into a cluster including *Methylosinus sporium* (Fig. S2).

### Phylogeny of metagenome sequences

Clone library analysis based on the Sanger method provided a high quality of sequences to elucidate phylogenetic relationships among rice root microbiomes (Figs. 2 and 3). However, it was not subjected to rigorous statistical analysis because relatively low numbers of the sequences (Table 1) were analyzed individually using combined DNA preparations from triplicate DNA samples. To statistically evaluate the phylogenetic compositions and functional aspects of the root microbiomes, metagenomic analyses were conducted by a pyrosequencer for the LN and SN root microbiomes with three replications (Table S1). A BLASTX search against the GenBank NR database assigned the resultant metagenome data of 369,021 reads from the LN roots to superkingdoms as 66.7% Bacteria, 0.9% Archaea, and 0.6% Eukaryota; and of 474,538 reads of the SN roots as 49.3% Bacteria, 3.4% Archaea, and 0.5% Eukaryota based on the total number of reads (Table S1).

**Fig. 4:**
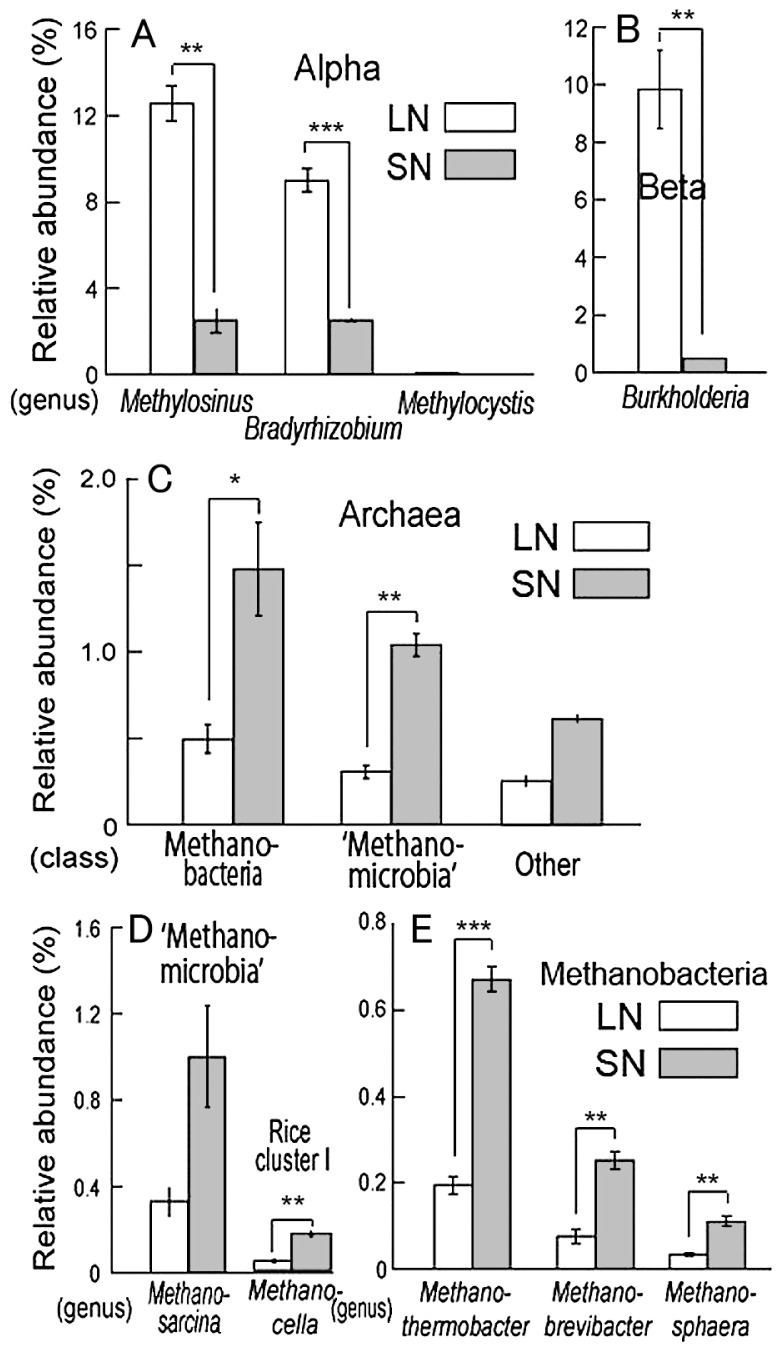
Phylogenetic compositions retrieved from the metagenomic data of rice root-associated bacteria from plants grown under low (LN) and standard (SN) N fertilization. Panels show the relative abundances of (A) *Alphaproteobacteria*, (B) *Betaproteobacteria*, (C) Archaea, (D) ‘*Methanomicrobia*’ and (E) *Methanobacteria*. Data were normalized on the basis of total number of reads (Table S1) for each biological replication (Table S1). Each bar shows the mean ± SEM. *0.01<*P*<0.05; **0.001<*P*<0.01; ***P<0.001 (*t*-test).

The phylogenetic compositions of bacteria from the metagenome data (Fig. 4) clearly confirmed the results of the comparative analyses of the 16S rRNA gene clone libraries for Bacteria domain (Fig. 2BCDE). In particular, the relative abundances of *Methylosinus*, *Bradyrhizobium* and *Burkholderia* were again significantly greater in LN roots than in SN roots (Fig. 4AB), consistent with the results of clone library analyses. In the Archaea domain, dominant taxa at the class level were *Euryarchaeota*, including ‘*Methanomicrobia*’ and *Methanobacteria*, the relative abundances of which were significantly lower in LN roots than in SN roots (Fig. 4CDE). *Methanosarcina* and *Methanocella*, which consist of a ubiquitous key methanogen group in rice paddy fields (40, 41), also showed reduced abundance in LN roots (Fig. 4D).

### Frequency of functional genes relevant to methane cycle

The frequency of functional genes was analyzed (35) (Table 2, Table S2). The abundances of the *pmo* and *mmo* genes, which encode particulate methane monooxygenase (pMMO) and soluble cytoplasmic methane monooxygenase (sMMO), were significantly greater in LN roots than in SN roots (Table 2). In contrast, *mcr* genes, which encode methyl-coenzyme M reductase, are functional genes of methanogenic archaea, were significantly less abundant in LN roots than in SN roots. When we determined the gene copy ratio of *pmoA*/*mcrA* by quantitative PCR of the DNA preparations, the *pmoA/mcrA* ratio of LN roots (1.66 ± 1.16; *n* = 3) was significantly higher than that of SN roots (0.32 ± 0.34, *n* = 6).

### ^13^C methane metabolism of LN and SN rice roots

To estimate the methane-oxidizing activity of methanotrphs inhabiting the root systems, the rice root systems were exposed to 10% (v/v) ^13^C-labeled methane (99.9 atom%). Although the ^13^C assimilated by methanotrophs in the root systems was markedly diluted with large amounts of unlabeled C in the rice roots, small but significant increases in ^13^C concentrations were observed by ^13^C-labeled methane exposure (Table 3). Base on total carbon content, dry weight of rice roots and the difference in ^13^C concentrations, the rate of ^13^C-methane assimilation was calculated (Table 3). As a result, the rate of incorporation of ^13^C-labeled methane gas into LN roots (81 nmol h^−1^ g^−1^ [dry weight]) was significantly higher than that into SN roots (42 nmol h^−1^ g^−1^ [dry weight]) (Table 3).

### Other genes impacted by low N fertilizer management

Differences in the abundance of other functional genes were also assessed (35). Gene abundance relevant to N, S, Fe, aromatics, and plant hormone metabolism was significantly higher in LN roots than in SN roots. The nominated functions were regarded as urea and nitrate utilization for N metabolism, sulfur oxidation and utilization of organic sulfur compounds for S metabolism, biosynthesis and transport of pyoverdine siderophore and ferrichrome iron receptor for Fe metabolism, utilization of benzoate, salicylate and phenylpropanoid for aromatics metabolism, and *acdS* and *iaaH* genes for plant hormone metabolism (Table 2). Based on BLAST X top hits, the organisms carrying *nifH* were different between LN and SN roots (Table S2). The *nifH* fragments were homologous to those of *Methylosinus* and *Bradyrhizobium* in LN roots, both of which have been observed with high abundance in the analyses of 16S rRNA genes (Fig. 2D) and metagenome phylogeny (Fig. 4A). On the other hand, SN roots harbored *nifH* fragments homologous to those of methanogenic archaea, *Deltaproteobacteria* and *Clostridia*, all of which are anaerobic N_2_-fixing organisms (Table S2).

### Bacterial communities in paddy field soils

**Fig. 5:**
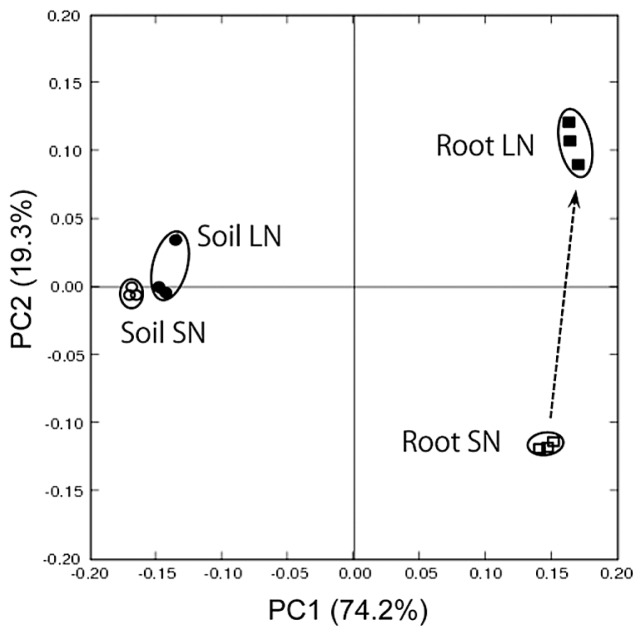
Principal coordinate analysis of the 16S rRNA gene libraries of bacterial communities in bulk soils (Soil) and rice roots (Root) under low (LN) and standard (SN) N fertilization conditions. The ordination was constructed using Unifrac distances. The percentage of variation explained by the plotted principal coordinates is indicated on the axes.

To examine whether the difference in bacterial communities between LN and SN microbiomes extends to paddy soil, deep sequencing of bacterial 16S rRNA genes was conducted for bulk soil DNA (LN and SN soils) with a pyrosequencer (Fig. S1, Table S3). PCoA by Fast UniFrac (13) demonstrated that soil and root samples were differentiated along PC1 (74.2%), while the communities of root-associated bacteria shifted from SN roots to LN roots along PC2 (19.3%) (Fig. 5). In contrast to root samples, soil samples formed a tight cluster (Fig. 5), indicating that soil microbial communities were less affected by the level of N fertilization as a whole. However, when the community structures of soil bacteria were examined in detail, three orders in *Proteobacteria* (*Rhizobiales*, *Rhodospirillales* and *Burkholderiales*) were shown to be significantly more abundant in LN soil than in SN soil (Fig. S3).

## Discussion

The present study revealed that N fertilizer management has a profound impact on the bacterial community of a rice paddy ecosystem. A relatively short period (5 years) without N fertilization caused a marked change in the composition of phylogenetic and functional diversities of the rice root microbiome (Figs. 2, 4). Withholding N fertilization also changed soil chemical properties, especially organic substance content and cation exchange capacity (CEC) (Table S5), although in contrast with the root microbiome, the soil bacterial communities remained relatively stable (Fig. 5), although the analysis of soil bacterial communities (Fig. S1, Table S3) suggests that the cessation of N input slightly changed the abundance of certain groups of *Proteobacteria* in the rice paddy field soil. The interactions between organic substances and cations (based on CEC) in soils influence nutrient availability (N, P, S) and root architecture (46). These data therefore suggest that no N input changed the root microbiome and soil properties via the plant–soil interface towards an ecosystem that was more likely to be sustainable, as discussed in the subsequent paragraphs.

Recently, Sessitsch *et al.* (43) reported metagenomic analysis of a bacterial endophyte community from surface-sterilized rice roots, showing many genes with endophytic lifestyles, such as plant polymer–degrading enzymes and detoxification of reactive oxygen species. However, our data showed more geochemical and nutritional aspects, because the bacterial cells included epiphytes and endophytes in rice roots under low N fertilization.

Intensive analyses of microbial communities in disease-suppressive soils have led to the identification of key microbes for disease suppression and their community functions (34). Thus, it is crucial to survey key microbes that support ecosystem functions by community analyses. Clone library analysis indicated that the root-associated bacterial community in the LN field harbored lower diversity than other communities examined (Table 1). Our analyses (Figs. 2DE, 4AB) demonstrated that *Burkholderia*, *Bradyrhizobium*, and *Methylosinus* became much more abundant in the LN root microbiome, indicating that they are potential key players in the low N soil environment. Phylogenetic analyses revealed that these dominant populations of *Burkholderia* and *Bradyrhizobium* are closely related to *B. kururiensis* KP23^T^ and the photosynthetic *Bradyrhizobium* sp. ORS287, respectively (Fig. 3BC).

*Burkholderia kururiensis* KP23^T^ was originally isolated from an aquifer polluted with trichloroethylene (50). Later, Mattos *et al.* (32) reported the ability of *B. kururiensis* to endophytically colonize rice plants and to promote both plant growth and rice grain yield in pot experiments. *B. kururiensis* KP23^T^ displays N_2_-fixing activity, IAA biosynthesis (32), and ACC deaminase activity (39). Interestingly, the roots of upland rice (*tropical japonica*) likely accommodated a bacterial species close to *B. kururiensis* (14). The *nifH*, *iaaMH* and *acdS* genes that are phylogenetically close to those of *Burkholderia* were also found in analyses of the root microbiome in the LN field (Tables 2, S2). Photosynthetic *Bradyrhizobium* has been reported as a natural endophyte of wild rice plants (6). Inoculation with *Bradyrhizobium* sp. ORS278 increased shoot growth and the grain yield of rice, indicating its potential ability to enhance rice production (6). We also found *nifH*, *iaaMH* and *acdS* genes that are phylogenetically close to those of *Bradyrhizobium* sp. ORS278 and BTAi1 in the root microbiome of the LN field (Tables 2, S2). The phylogenetic trees of *nifH* and *acdS* genes are generally congruent with the 16S rRNA gene tree (3, 49). Therefore, it is conceivable that the *Burkholderia* and *Bradyrhizobium* species that carry *nifH*, *iaaMH* and *acdS* were dominant in the rice roots of the LN field, suggesting that these microbes could support plant growth in nutrient-poor environments by performing their N_2_-fixing activity, IAA biosynthesis, and ACC deaminase activity.

In general, the methane cycle occurs in rice paddy field ecosystems: methanogens produce methane from root exudates or other organic materials in anoxic bulk soil, and the methane diffuses to the rice roots, where active methane oxidation occurs by methanotrophs (5). A recent study suggested that the rice root-associated microbiome sustains both methanogenesis and methanotrophs (4, 24). Here, the abundance of methanogens was significantly reduced in the LN root microbiome rather than the SN microbiome (Fig. 4CD). Interestingly, these methanogens are known to be preferentially associated with roots (27), suggesting that plant–methanogen interaction is considerably affected by fertilization management.

In LN roots, the frequencies of *pmo* and *mmo* genes were significantly increased (Table 2) in parallel with an increase in the abundance of methanotroph in LN roots (*Methylosinus* in Figs. 2D, 4A). Type II methanotrophs (*Methylocystis* and *Methylosinus*) are considered to be indicative species of survival under adverse conditions (5, 24). In contrast, the frequency of *mcr* genes tended to decrease in LN roots (Table 2), consistent with the reduced abundance of methanogenic archaea in LN roots (Fig. 4CDE). The ratio of *pmoA*/*mcrA* and the ^13^C-labeled methane incorporation rate increased significantly in LN roots compared with SN roots. Therefore, our results suggest that methane oxidizers were relatively abundant in LN roots than in SN roots compared to methanogens in terms of gene copy number. As for activity (Table 3), the root microbiome would have higher methane-oxidizing activity in the LN field than in the SN field, because methane- oxidizing bacteria generally use CH_4_ as their sole carbon source (38).

There is ongoing debate about the possible effects of N fertilizer application on methane emissions from rice fields. Nitrogen fertilizer often reduced methane emissions from experimental microcosms (5) and fields (48), which likely depends on the dose and type of nitrogen fertilizers by meta-analysis (1). Because the net impact of nitrogen fertilizers on methane emissions was affected by many abiotic and biotic factors of rice paddy ecosystems including bulk soils, rhizosphere soils and rice plants, it is crucial to understand the underlying mechanisms (4). Our results suggest that low N fertilization management could change the rice root microbiomes relevant to methane cycles, methanogenesis and methanotrophs.

The functional genes for the metabolism of N, S, Fe, and aromatics were more abundant in LN roots than in SN roots (Table 2). Searches of the gene functions led us to speculate that no input of N fertilizer may affect not only the methane cycle, but also other biogeochemical processes of a wide variety of substances that facilitate adaptation to low-nutrient environments. For example, the bacteria associated with rice roots in the absence of N fertilizer can utilize urea-related compounds and nitrate as N sources (46). Such a microbial community can also efficiently utilize organic S (23, 45) and pyoverdine siderophores (8) as S and Fe sources, respectively. Low N-input management substantially reduced rice growth (Figs. 1, S1): presumably, then, root exudates were less available under LN than under SN. Thus, root microbiomes under the condition of low N input may be capable of catabolizing recalcitrant substances in the soil, such as aromatic compounds, as C, N and S sources to alleviate nutrient deficiencies. Abundant genes for Fe acquisition and transport (Table 2) may also accelerate competitive colonization by typical symbiotic bacteria like *Pseudomonas putida* KT2440 (36) of roots under poor nutrient conditions.

The LN root microbiome harbored more abundant functional marker genes for sulfur oxidation (*sox*) (31) and methane oxidation (*pmoA* and *mmoX*) than the SN root microbiome (Table 2), suggesting a more oxidative environment in the LN field than in the SN field. In contrast, from the *nifH* phylogeny, anaerobic microbes (methanogenic archaea, *Anaeromyxobacter*, and *Clostridium*) were found exclusively in the SN root microbiome (Tables 2, S2). These findings suggest that the microbial community of the rice rhizosphere had adapted to a more aerobic environment during a relatively short period (5 years) with no input of N fertilizer management.

Low N fertilization management often changes soil chemistry, including the enhancement of soil organic carbon (37). The no-N-fertilizer treatment here represents an extreme form of field management for the purposes of basic research because of rice biomass reduction in the LN field. However, the results here show that no-N-fertilizer treatment enhanced the abundance of specific microbes OTUs BP2 (*Burkholderia* sp.), AP31 (*Methylosinus* sp.) and AP36 (*Bradyrhizobium* sp.), and functional genes for the metabolism of N, S, Fe and aromatics. In addition, active methane oxidation likely occurred in the rice roots of the LN field. For further validation of these organisms and geochemical processes as biological makers, it would be fascinating to isolate and characterize the presumptive key players, such as *Burkholderia*, *Methylosinus* and *Bradyrhizobium*, and to seek a way to technologically regulate them toward more beneficial geochemical/nutritional processes and plant associations in agricultural settings.

## Supplementary Materials



## Figures and Tables

**Table 1:** Statistical analysis of 16S rRNA gene clone libraries derived from rice shoot- and root-associated bacteria^a^

Tissue	Shoot			Root		
		
N fertilization level^b^	LN	SN	HN	LN	SN	HN
Statistics						
No. of sequences	132	151	159	112	91	106
Library coverage, *C_x_* (%)^c^	71.2	69.5	75.5	82.1	61.5	70.8
No. of OTUs^d^	59	71	62	33	44	53
Diversity indexes						
Shannon index (*H*′)	3.7	3.9	3.7	2.8	3.2	3.7
Simpson index (1/*D*)	33.1	36.7	30.7	10.1	13.8	38.9

a Abbreviations: OTUs, operational taxonomic units; rRNA, ribosomal RNA. The lower values of number of OTUs and diversity indexes are shaded in grey.

b The LN, SN, and HN indicate N fertilization at low, standard, and high levels (see main text).

c *C_x_* = 1 − (*n_x_*/N), where *n_x_* is the number of singletons encountered only once in a library and *N* is the total number of clones.

d OTUs were defined at 95% sequence identity. Shaded values are the lowest number of OUT and diversity indexes.

**Table 2:** Frequency of functional genes retrieved from metagenomic data of the bacterial community of rice roots grown under low (LN) and standard (SN) N fertilization^a^

Category	Subcategory or function	Gene	Frequency (10^−5^)		Ratio LN/SN	*P t*-test

			LN	SN		
N_2_ fixation						
	Nitrogenase reductase	*nifH*	13.4	9.0	1.5	0.111
Methane cycle						
	Particulate methane monooxygenase	*pmo*	28.8	7.0	4.1	0.003
	Soluble methane monooxygenase	*mmo*	27.5	5.9	4.7	0.000
	Methyl-coenzyme M reductase	*mcr*	3.8	10.8	0.4	0.020
Plant hormone related						
	ACC deaminase	*acdS*	6.1	1.3	4.7	0.011
	Tryptophan-2-monooxygenase	*iaaM*	5.4	0.1	44.5	0.075
	Indoleacetamide hydrolase	*iaaH*	8.5	1.9	4.5	0.009
	Indole-3-pyruvate decarboxylase	*ipdC*	0.1	0.3	0.4	0.219
N metabolism						
	Urease	*ure*	6.0	1.8	3.3	0.016
	Urea carboxylase		5.4	1.6	3.4	0.049
	Urea transporter		27.3	8.7	3.1	0.001
	Nitrate transporter		13.9	4.1	3.4	0.000
S metabolism						
	Sulfur oxidation	*sox*	11.3	4.4	2.6	0.008
	Alkensulfonate transporter		34.3	15.1	2.3	0.000
	Desulfurization enzyme	*ssu*	23.7	10.7	2.2	0.000
Fe metabolism						
	Pyoverdin biosynthesis	*pvd*	62.6	27.8	2.3	0.001
	Ferrichrome transport		56.6	23.0	2.5	0.002
Aromatics metabolism						
	Benzoate (aerobic path)		21.6	10.8	2.0	0.002
	Benzoate (anaerobic path)		13.4	5.8	2.3	0.013
	Salicylate and salicylate ester		2.1	0.3	7.6	0.010
	Phenylpropanoid		23.3	8.0	2.9	0.021

a Gray shading shows values for which a significant difference exists between the LN and SN roots. Frequency shows the ratios of gene numbers in each functional category (Table S2) to total number of reads for three biological replications with different MID barcodes (Table S1). *P* indicates probability value of *t*-test between the LN and SN root microbiomes for each functional category. See the explanations of Subcategory or function in Supplementary Materials and Methods.

**Table 3:** Incorporation of ^13^C from labeled methane gas into rice root systems under low (LN) and standard (SN) N fertilization*

Rice field	Gas phase	^13^C concentration			CH_4_ assimilation by methanotrophs	
	
		(‰)	(atom%)	(atom% excess)	(μmol plant^−1^)	(nmol h^−1^ g^−1^ dry root weight)
LN	None	−28.2 ± 0.0	1.075 ± 0.000	—	—	—
LN	^13^CH_4_	−21.7 ± 1.4	1.082 ± 0.002	0.0071 ± 0.0015^‡^	11.2 ± 1.9^†^	81 ± 22^†^
SN	None	−28.1 ± 0.1	1.075 ± 0.000	—	—	—
SN	^13^CH_4_	−24.5 ± 1.7	1.079 ± 0.002	0.0039 ± 0.0019	5.6 ± 1.7	42 ± 14

* Rice roots were sampled from the LN and SN paddy fields at the booting stage, and then washed well with tap water. The root systems were exposed to 10% (v/v) ^13^C-labeled methane at 25°C for 26 h. The incorporation of ^13^C from ^13^C-labeled methane gas into the rice root systems was determined in triplicate from ^13^C concentration, C content, and dry weight. Values are the means ± standard deviation of triplicate determinations. Asterisks indicate significant differences between LN and SN roots by *t* test (^†^*P* <0.05, ^‡^*P* <0.01)
