# Supplementary material for: Low Nitrogen Fertilization Adapts Rice Root Microbiome to Low Nutrient Environment by Changing Biogeochemical Functions
Source: Microbes Environ. 2014 Jan 24;29(1):50–9. doi: 10.1264/jsme2.ME13110 (PMC4041235; doi:10.1264/jsme2.ME13110)
Supplement: Supplementary file 1 [file 29_50_s1.pdf]

Supplementary Materials

Low nitrogen fertilization adapts rice root microbiome to low nutrient environment by changing biogeochemical functions

Seishi Ikeda,[a,b] Kazuhiro Sasaki,[a] Takashi Okubo,[a] Akifumu Yamashita,[a] Kimihiro Terasawa,[a] Zhihua Bao,[a] Dongyan Liu,[c] Takeshi Watanabe,[c] Jun Murase,[c] Susumu Asakawa,[c] Shima Eda,[a] Hisayuki Mitsui,[a] Tadashi Sato,[a] Kiwamu Minamisawa[a]

[a] Graduate School of Life Sciences, Tohoku University, Katahira, Aoba-ku, Sendai, Miyagi 980-8577, Japan

[b] Memuro Research Station, National Agricultural Research Center for Hokkaido Region, Shinsei, Memuro-cho, Kasaigun, Hokkaido 082-0081, Japan

[c] Soil Biology and Chemistry, Graduate School of Bioagricultural Sciences, Nagoya University, Chikusa, Nagoya 464-8601, Japan

Supplementary Material and Methods

Sequence analysis of clone libraries for 16S rRNA genes

The PCR clone libraries for 16S rRNA genes were constructed and sequenced by the Sanger method as described (1, 2). Library coverage was calculated with the non-parametric estimator *C* (3) as described by Kemp and Aller (4). The reciprocal of Simpson's index (1/*D*) was used as a measure of diversity to evaluate the level of dominance in a community (5). The number of shared OTUs between libraries was calculated using SONS (6). UniFrac (7) was used to examine the differences between clone libraries. A tree file generated by CLUSTALW (8) and an environment file, which links a file to a library, were uploaded to UniFrac (7). Principal coordinate analysis (PCoA) was performed in UniFrac with the abundance-weighted option (7).

Metagenome sequencing of rice root–associated bacteria

DNAs of the enriched bacterial cells from rice roots grown under low N (LN) and standard N (SN) fertilization were shotgun-sequenced on a 454 GS FLX Titanium pyrosequencer (Roche Applied Science, Mannheim, Germany). Approximately 500 ng of DNA was fragmented with a Covaris Model S1 sonicator (Covaris, Woburn, MA). Triplicate shotgun libraries derived from biologically independent root samples (LN or SN roots) were constructed using an MID (barcode) Adaptor Kit (Roche Applied Science). After titration, 2.7 DNA-copies per bead were used for the main sequencing run. After emulsion PCR and subsequent bead recovery, the DNA beads for LN or SN roots with three MIDs were loaded onto each half of the Pico TiterPlate and pyrosequenced. For all analyses, replicate reads were removed

using a 454 Replicate Filter (Gomez-Alvarez et al. 2009) with the following parameters: 0.9 (identity > 90%), length difference requirement = 0, and three beginning banes checked (9). Metagenomic reads in each sample were taxonomically assigned according to the best-hit pairs in the BLASTX analysis against the GenBank nr database with an e-value threshold of 10^{-10}. Gene IDs for all hits were collected and phylogenetically placed with an in-house script.

Prediction of functional gene categories from metagenome sequences

The metabolic potentials of each microbiome were assessed by BLASTX (E-value < 10^{-5}) search against the SEED database using the MG-RAST server (10). MG-RAST assigns sequences to three hierarchical levels of metabolic subsystems, which consist of groups of genes that comprise a metabolic function. Our metagenomic reads were uploaded to the MG-RAST Web site (http://metagenomics.nmpdr.org/).

Differences in the functional gene abundances of the root microbiomes between the LN and SN roots were assessed by a public resource for the automatic functional analysis of metagenomes, MG-RAST (Metagenome Rapid Annotation using Subsystem Technology) (10). We selected subcategories whose abundance was significantly >2× in LN roots than in SN roots ($P < 0.01$). We then combined the respective abundances of the subcategory names according to functional aspects and recalculated the combined abundances and their statistics (Table 1). The subcategories in MG-RAST contained genes for synthesis of the following proteins: Urease alpha, beta, and gamma subunits (EC 3.5.1.5) and urease accessory protein UreDEFG; urea ABC transporter, urea ABC transporter and urea carboxylase-related ABC transporter: nitrate ABC transporter, nitrate ABC transporter and nitrate/nitrite transporter: sulfur

oxidation, sulfur oxidation protein SoxRSWXYZABCDH; alkanesulfonate transporter; alkanesulfonates ABC transporter, ABC-type nitrate/sulfonate/bicarbonate transport system and alkanesulfonates transport system permease protein; desulfurization enzyme, alkanesulfonate monooxygenase (EC 1.14.14.5), alpha-ketoglutarate-dependent taurine dioxygenase (EC 1.14.11.17), sulfonate monooxygenase, arylsulfatase (EC 3.1.6.1), organosulfonate utilization protein SsuF, alkanesulfonate-binding protein, probable dibenzothiophene desulfurization enzyme, dibenzothiophene desulfurization enzyme B and FMN reductase (EC 1.5.1.29). ACC (1-aminocyclopropane-1-carboxylic acid) deaminase (EC 3.5.99.7) degrades ACC, an intermediate of ethylene biosynthesis in plants. IAA (indole-3-acetic acid) is a plant hormone that is biosynthesized via IAM (indole-3-acetoamide) or IPyA (indole-3-pyruvic acid) pathways. (EC 1.13.12.3). IAM pathway is mediated by Tryptophan-2-monooxygenase (EC 1.13.12.3) and Indoleacetamide hydrolase (EC 3.5.1.-), which are encoded by *iaaM* and *iaaH* genes. Indole-3-pyruvate decarboxylase (EC 4.1.1.74) encoded by *ipdC* gene is a key enzyme for IPyA pathway.

Pyrosequencing of 16S rRNA genes of soil- and root-associated bacteria

For each DNA sample, we amplified the 16S rRNA genes using a primer set, Bac-27F (5′-CCTATCCCCTGTGTGCCTTGGCAGTCTCAG_agagtttgatcmtggctca-3′), and MID-518R (5′-CCATCTCATCCCTGCGTGTCTCCGACTCAG_ barcode (MID)_ ttaccgcggctgctgg-3′), in which the nucleotide sequences shown in lower case are universal sequences of bacterial 16S rRNA genes. The MID-518R primer contains the sequences of the Titanium A adaptor, Key sequence (TCAG), and barcode sequences (MIDs). The Bac-27F primer contains the sequences of the Titanium B adaptor and

Key sequence. These primers target the V1–V3 regions (11-13). A sample of 5 ng of total DNA was used as the template in a final reaction volume of 12 μL, including 25 pmol of each primer and 2 U of Ex Taq HS DNA polymerase (Takara Bio, Otsu, Japan). Cycling conditions were as follows: initial denaturation for 2 min at 94 °C; 25 cycles of 30 s at 94 °C, 30 s at 55 °C, and 90 s at 72 °C; and a final extension for 10 min at 72 °C. The PCR products were resolved by 1% agarose gel electrophoresis in 1 × TAE buffer. PCR products of the predicted size (~500 and ~700 bp) were extracted from the gels using a Wizard SV gel and PCR Clean-Up System (Promega, Tokyo, Japan). Products were sequenced by a 454 GS FLX Titanium pyrosequencer. The 16S rRNA gene sequences were assigned to each sample according to sample-specific barcodes. Sequences were then checked for (i) perfect match with primers; (ii) length of >250 nucleotides without barcode and primer sequences; and (iii) no ambiguous nucleotide sequences (denoted by N). Only the first 250-bp after the proximal PCR primer of each sequence was trimmed, because the quality of sequences became degraded beyond this point. Taxonomy was assigned using the Ribosomal Database Project (RDP) MultiClassifier with a minimum support threshold of 50% (14). The Fast UniFrac algorithm (15) was used to determine the degree of bacterial diversity shared between communities. The Greengenes core set (16) was chosen as the reference tree. Our 16S rRNA sequences were mapped to their closest relative in the reference tree using BLAST's megablast protocol (17), and hits with an E-value < $1e^{-30}$ were considered to be significant. The sample ID mapping file, which links the sequence ID in the reference tree to the sample ID that it came from, was uploaded to the Fast UniFrac Web site (http://www.bmf.colorado.edu/fastunifrac). PCoA was performed with the abundance-weighted option.

References

1. Ikeda, S., T. Okubo, T. Kaneko, et al. 2010. Community shifts of soybean-associated bacteria responding to different nodulation phenotypes and N levels. ISME J. 4:315–326.
2. Ikeda, S., M. Anda, S. Inaba, et al. 2011. Autoregulation of nodulation interferes with impact of nitrogen fertilization levels on the leaf-associated bacteria community in soybeans. Appl. Environ. Microbiol. 77:1973–1980.
3. Good, I.J. 1953. The population frequencies of species and the estimation of population parameters. Biometrika 40:237–264.
4. Kemp, P.F., and J.Y. Aller. 2004. Bacterial diversity in aquatic and other environments: What 16S rDNA libraries can tell us. FEMS Microbiol. Ecol. 47:161–177.
5. Zhou, J., B. Xia, D.S. Treves, L.Y. Wu, T.L. Marsh, R.V. O'Neill, A.V. Palumbo, and J.M. Teidje. 2002. Spatial and resource factors influencing high microbial diversity in soil. Appl. Environ. Microbiol. 68:326–334.
6. Schloss, P.D., and J. Handelsman. 2005. Introducing DOTUR, a computer program for defining operational taxonomic units and estimating species richness. Appl. Environ. Microbiol. 71:1501–1506.
7. Lozupone, C., and R. Knight. 2005. UniFrac: A new phylogenetic method for comparing microbial communities. Appl. Environ. Microbiol. 71:8228–8235.
8. Thompson, J.D., D.G. Higgins, and T.J. Gibson. 1994. CLUSTAL W: Improving the sensitivity of progressive multiple sequence alignment through sequence weighting, position-specific gap penalties and weight matrix choice. Nucleic Acids Res. 22:4673–4680.
9. Gomez-Alvarez, V., T.K. Teal, and T.M. Schmidt. 2009. Systematic artifacts in metagenomes from complex microbial communities. ISME J. 3:1314–1317.
10. Meyer, F., D. Paarmann, M. D'Souza M, et al. 2008. The metagenomics RAST

server – a public resource for the automatic phylogenetic and functional analysis of metagenomes. BMC Bioinformatics 9:386.

11. Wei, H., L. Dong, T. Wang, et al. 2010. Structural shifts of gutmicrobiota as surrogate endpoints for monitoring host health changes induced bycarcinogen exposure. FEMS Microbiol. Ecol. 73:577–586.

12. Chun, J., K.Y. Kim, J.H. Lee, and Y. Choi. 2010. The analysis of oral microbial communities of wild-type and toll-like receptor 2-deficient mice using a 454 GS FLX Titanium pyrosequencer. BMC Microbiol. 10:101

13. Weisburg, W.G., SM. Barns, D.A. Pelletier, D.J. Lane. 1991. 16S ribosomal DNA amplification for phylogenetic study. J. Bacteriol. 173:697–703.

14. Wang, Q.G., M. Garrity, J.M. Tiedje, and J.R. Cole. 2007. Naïve bayesian classifier for rapid assignment of rRNA sequences into the new bacterial taxonomy. Appl. Environ. Microbiol. 73:5261–5267.

15. Hamady, M., C. Lozupone, and R. Knight. 2009. Fast UniFrac: facilitating high-throughput phylogenetic analyses of microbial communities including analysis of pyrosequencing and PhyloChip data. ISME J. 4:17–27.

16. DeSantis, T.Z., P. Hugenholtz, N. Larsen, et al. 2006. Greengenes, a chimera-checked 16S rRNA gene database and workbench compatible with ARB. Appl. Environ. Microbiol. 72:5069–5072.

17. Altschul, S.F., W. Gish, W. Miller, E.W. Myers, and D.J. Lipman. 1990. Basic local alignment search tool. J. Mol. Biol. 215:403–410.

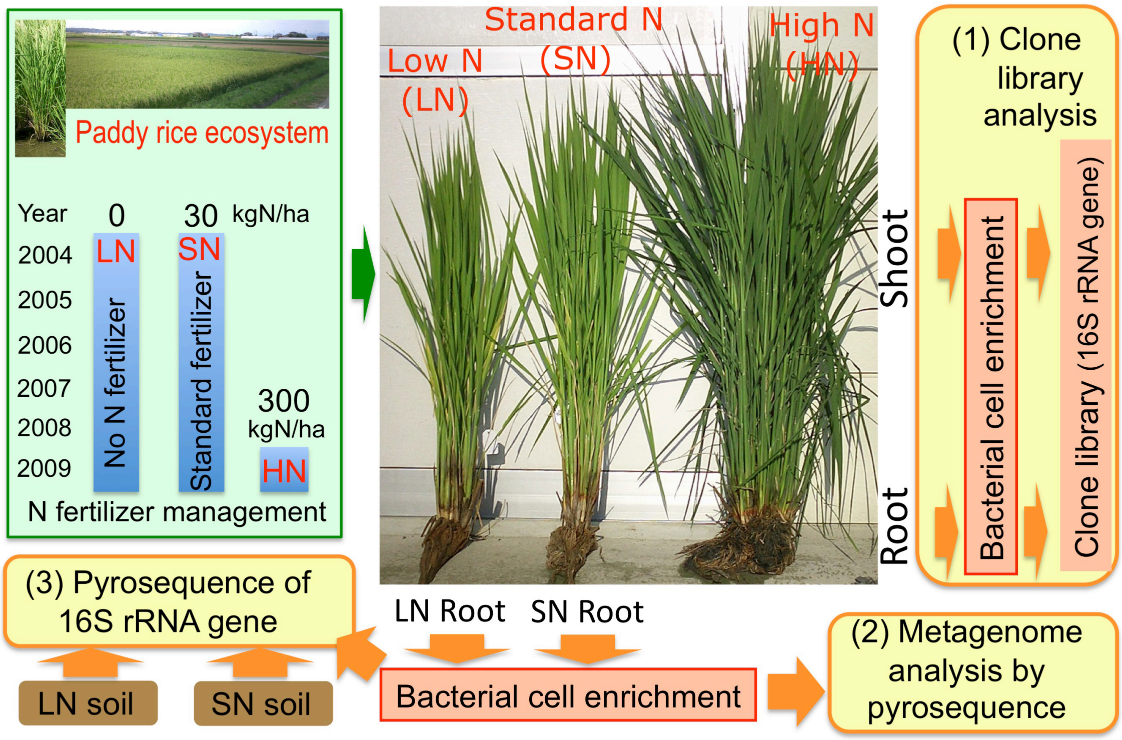

Fig. S1. Schematic of the experimental strategy used to characterize the microbiota of a rice paddy ecosystem under low (LN), standard (SN), and high (HN) N fertilization. Prior to the experiment in 2009, LN field received no N fertilizer for 5 years.

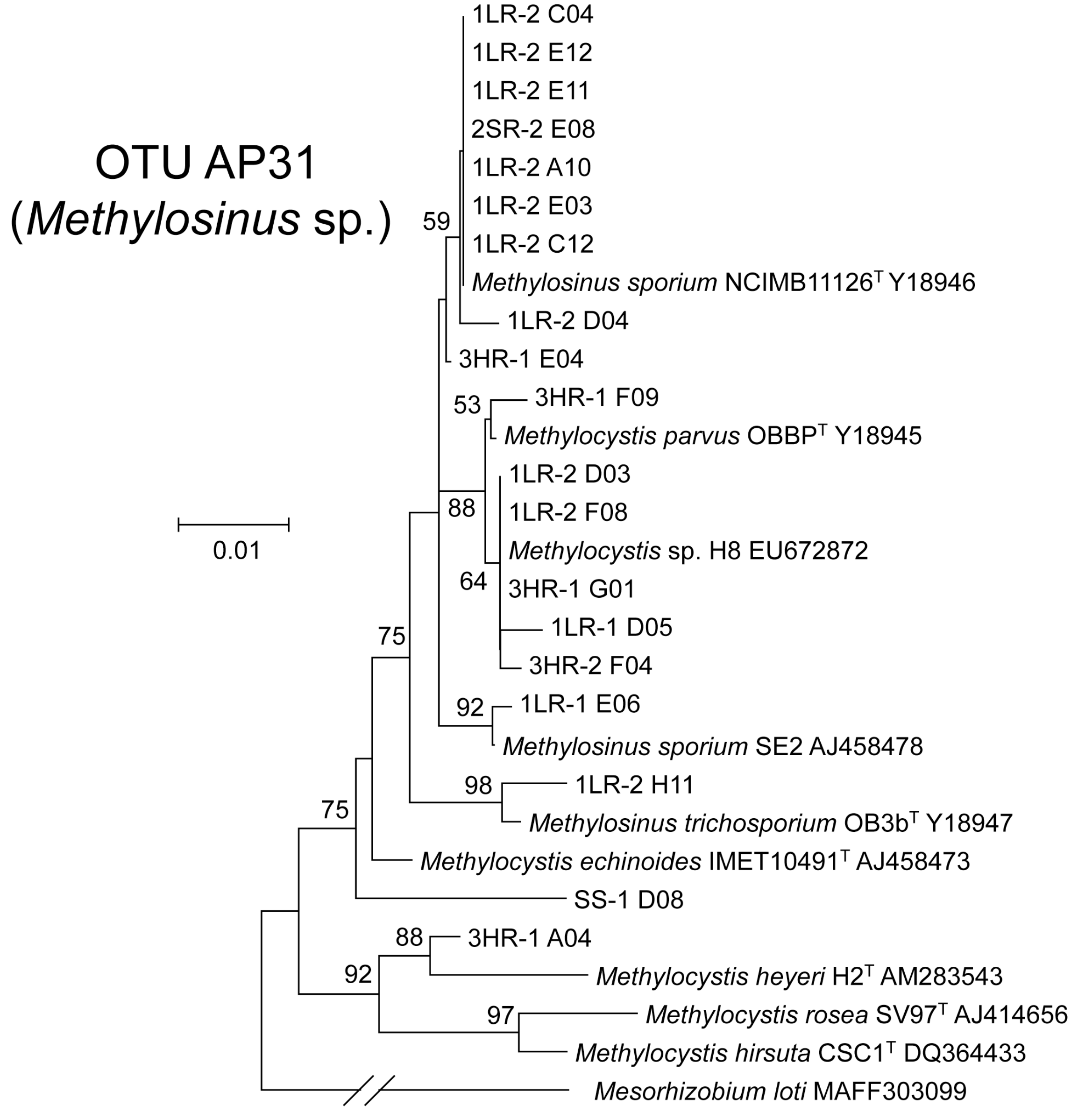

Fig. S2. Phylogenetic tree of OTU AP31 (see also Fig. 3 and text) represented by *Methylosinus* sp. The tree was constructed by the neighbor-joining method. The scale represents 0.01 substitutions per site. The numbers at the nodes are the proportions of 1000 bootstrap resamplings, and values of <50% are not shown.

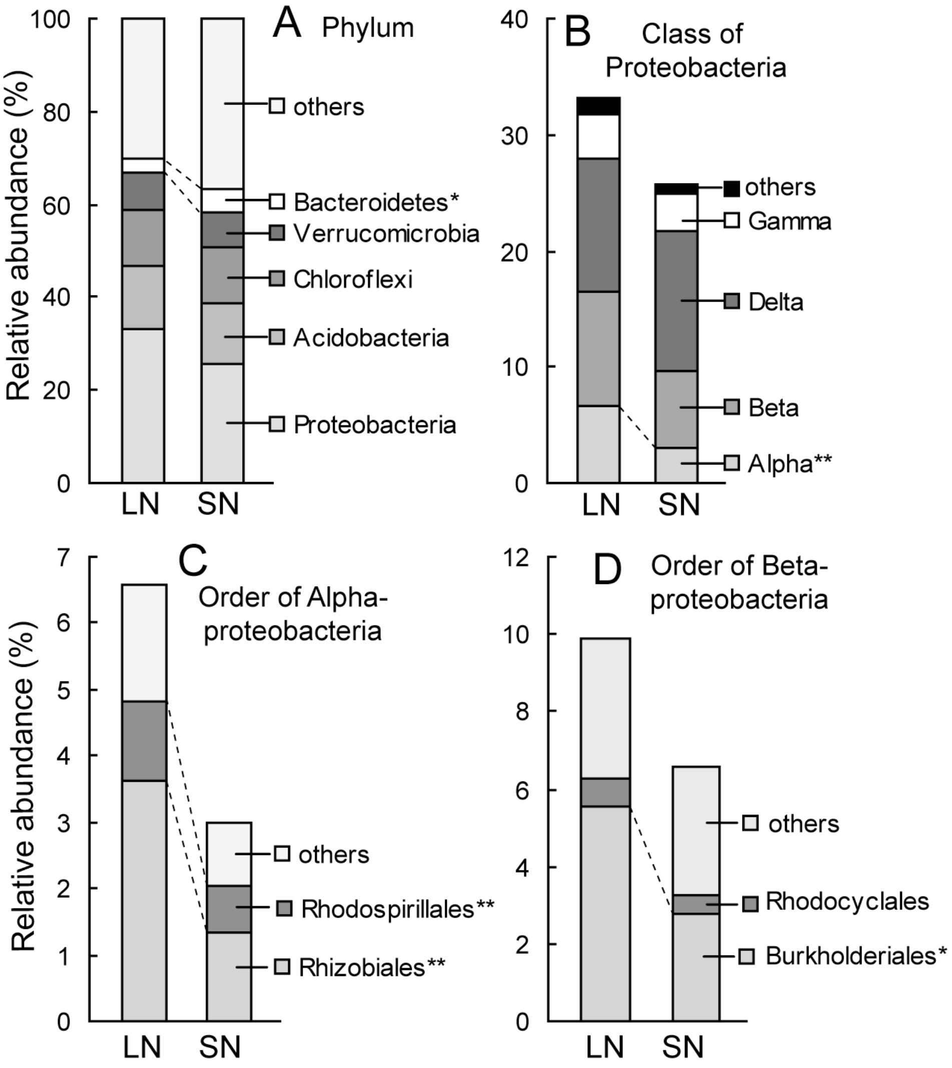

Fig. S3. Relative abundance of the dominant taxa found in 16S rRNA gene clone libraries of paddy soil bacteria under low (LN) and standard (SN) N fertilization regimes at different taxonomic levels: (A) phylum and (B) class for Proteobacteria; order for (C) Alphaproteobacteria and (D) Betaproteobacteria. Asterisks indicate significant differences (Student's *t*-test, $*P < 0.05$, $**P < 0.01$) by pyrosequencing with three barcodes (MIDs) using three DNA samples independently extracted from each soil.

Table S1. Summary of metagenomic reads from the rice root-associated microbiome of rice plants grown under low (LN) and standard (SN) N fertilization*

N level	LN			SN		
Barcode	MID 4	MID 5	MID 6	MID 1	MID 2	MID 3
Total reads						
No. of total reads (A)	401,675	114,036	80,385	227,660	395,794	49,058
Read length (Mb)	147.4	42.0	30.3	85.3	152.1	18.8
Average read length (bp)	366.9	368.4	376.4	374.6	384.3	383.3
Unique reads						
No. of unique reads	248,856	70,360	49,805	162,300	276,440	35,798
Read length (Mb)	94.0	26.8	19.2	62.1	108.6	14.0
Average read length (bp)	377.8	380.2	386.1	382.4	392.9	391.1

* Three independent DNA samples tagged with different barcodes (MIDs) were pyrosequenced (see text and Supplementary materials). Each DNA sample was independently analyzed from field sampling (a composite sample) to pyrosequencing.

Gomez-Alvarez V, Teal TK, Schmidt TM. 2009. Systematic artifacts in metagenomes from complex microbial communities. ISME J. **3**:1314–1317.

Table S2. List of functional genes from matagenome data of rice root microbiomes in LN and SN fields. The functional genes for methane oxidation (pmo and mmo), metanogenesis (mcr), and IAA (indole-3-acetic acid) biosynthesis (iaaMH and ipdC), and ACC (1-aminocyclopropane) deaminase (acdS) were listed.

Gene	Sample	MID	query name	Category	hit name	domain	phylum	class	order	organisms	identity (%)	e-value	annotation
nifH	LN Root	MID4	GJHFW2E02HT469	Nitrogen_fixation	ref|YP_001207334.1|	Bacteria	Proteobacteria	Alphaproteobacteria	Rhizobiales	Bradyrhizobium sp. ORS278	100	2.00E-58	nitrogenase reductase emb|CAL79117.1|
nifH	LN Root	MID4	GJHFW2E02F3ASE	Nitrogen_fixation	ref|YP_001207334.1|	Bacteria	Proteobacteria	Alphaproteobacteria	Rhizobiales	Bradyrhizobium sp. ORS278	81	2.00E-37	nitrogenase reductase emb|CAL79117.1|
nifH	LN Root	MID5	GJHFW2E02FMECL	Nitrogen_fixation	ref|YP_001241728.1|	Bacteria	Proteobacteria	Alphaproteobacteria	Rhizobiales	Bradyrhizobium sp. BTAi1	95	2.00E-15	nitrogenase reductase gb|ABQ37822.1|
nifH	LN Root	MID6	GJHFW2E02JVWXP	Nitrogen_fixation	ref|YP_001207334.1|	Bacteria	Proteobacteria	Alphaproteobacteria	Rhizobiales	Bradyrhizobium sp. ORS278	84	3.00E-57	nitrogenase reductase emb|CAL79117.1|
nifH	LN Root	MID6	GJHFW2E02FGPHB	Nitrogen_fixation	ref|YP_001207334.1|	Bacteria	Proteobacteria	Alphaproteobacteria	Rhizobiales	Bradyrhizobium sp. ORS278	93	4.00E-25	nitrogenase reductase emb|CAL79117.1|
nifH	LN Root	MID6	GJHFW2E02HXKE9	Nitrogen_fixation	ref|YP_004111076.1|	Bacteria	Proteobacteria	Alphaproteobacteria	Rhizobiales	Rhodopseudomonas palustris DX-1	85	2.00E-22	nitrogenase iron protein gb|ADU46343.1|
nifH	LN Root	MID4	GJHFW2E02GSH2A	Nitrogen_fixation	ref|YP_004677463.1|	Bacteria	Proteobacteria	Alphaproteobacteria	Rhizobiales	Hyphomicrobium sp. MC1	96	2.00E-41	nitrogenase iron protein 2 emb|CCB66897.1| Nitrogenase iron protein 2
nifH	LN Root	MID4	GJHFW2E02H3BBD	Nitrogen_fixation	ref|YP_004677463.1|	Bacteria	Proteobacteria	Alphaproteobacteria	Rhizobiales	Hyphomicrobium sp. MC1	91	3.00E-41	nitrogenase iron protein 2 emb|CCB66897.1| Nitrogenase iron protein 2
nifH	LN Root	MID4	GJHFW2E02I6RLM	Nitrogen_fixation	ref|YP_004677463.1|	Bacteria	Proteobacteria	Alphaproteobacteria	Rhizobiales	Hyphomicrobium sp. MC1	98	5.00E-43	nitrogenase iron protein 2 emb|CCB66897.1| Nitrogenase iron protein 2
nifH	LN Root	MID4	GJHFW2E02JIHJF	Nitrogen_fixation	ref|YP_004677463.1|	Bacteria	Proteobacteria	Alphaproteobacteria	Rhizobiales	Hyphomicrobium sp. MC1	91	7.00E-39	nitrogenase iron protein 2 emb|CCB66897.1| Nitrogenase iron protein 2
nifH	LN Root	MID4	GJHFW2E02IJJ7H	Nitrogen_fixation	ref|YP_004677463.1|	Bacteria	Proteobacteria	Alphaproteobacteria	Rhizobiales	Hyphomicrobium sp. MC1	91	4.00E-40	nitrogenase iron protein 2 emb|CCB66897.1| Nitrogenase iron protein 2
nifH	LN Root	MID5	GJHFW2E02JK183	Nitrogen_fixation	ref|YP_004677463.1|	Bacteria	Proteobacteria	Alphaproteobacteria	Rhizobiales	Hyphomicrobium sp. MC1	97	5.00E-78	nitrogenase iron protein 2 emb|CCB66897.1| Nitrogenase iron protein 2
nifH	LN Root	MID5	GJHFW2E02FISO6	Nitrogen_fixation	ref|YP_004677463.1|	Bacteria	Proteobacteria	Alphaproteobacteria	Rhizobiales	Hyphomicrobium sp. MC1	98	1.00E-61	nitrogenase iron protein 2 emb|CCB66897.1| Nitrogenase iron protein 2
nifH	LN Root	MID4	GJHFW2E02F24XL	Nitrogen_fixation	ref|ZP_08073552.1|	Bacteria	Proteobacteria	Alphaproteobacteria	Rhizobiales	Methylocystis sp. ATCC 49242	100	3.00E-07	nitrogenase iron protein gb|EFX98811.1| nitrogenase iron protein
nifH	LN Root	MID4	GJHFW2E02F63TS	Nitrogen_fixation	ref|ZP_06886595.1|	Bacteria	Proteobacteria	Alphaproteobacteria	Rhizobiales	Methylosinus trichosporium OB3b	98	9.00E-45	nitrogenase iron protein gb|EFH04962.1| nitrogenase iron protein
nifH	LN Root	MID4	GJHFW2E02GITN9	Nitrogen_fixation	ref|ZP_06886595.1|	Bacteria	Proteobacteria	Alphaproteobacteria	Rhizobiales	Methylosinus trichosporium OB3b	97	5.00E-70	nitrogenase iron protein gb|EFH04962.1| nitrogenase iron protein
nifH	LN Root	MID4	GJHFW2E02IAKLU	Nitrogen_fixation	ref|ZP_06886595.1|	Bacteria	Proteobacteria	Alphaproteobacteria	Rhizobiales	Methylosinus trichosporium OB3b	98	5.00E-73	nitrogenase iron protein gb|EFH04962.1| nitrogenase iron protein
nifH	LN Root	MID4	GJHFW2E02H06DZ	Nitrogen_fixation	ref|ZP_06886595.1|	Bacteria	Proteobacteria	Alphaproteobacteria	Rhizobiales	Methylosinus trichosporium OB3b	93	4.00E-74	nitrogenase iron protein gb|EFH04962.1| nitrogenase iron protein
nifH	LN Root	MID4	GJHFW2E02HFLP5	Nitrogen_fixation	ref|ZP_06886595.1|	Bacteria	Proteobacteria	Alphaproteobacteria	Rhizobiales	Methylosinus trichosporium OB3b	92	2.00E-38	nitrogenase iron protein gb|EFH04962.1| nitrogenase iron protein
nifH	LN Root	MID4	GJHFW2E02HRIOI	Nitrogen_fixation	ref|ZP_06886595.1|	Bacteria	Proteobacteria	Alphaproteobacteria	Rhizobiales	Methylosinus trichosporium OB3b	96	3.00E-27	nitrogenase iron protein gb|EFH04962.1| nitrogenase iron protein
nifH	LN Root	MID5	GJHFW2E02F1H2P	Nitrogen_fixation	ref|ZP_06886595.1|	Bacteria	Proteobacteria	Alphaproteobacteria	Rhizobiales	Methylosinus trichosporium OB3b	89	4.00E-06	nitrogenase iron protein gb|EFH04962.1| nitrogenase iron protein
nifH	LN Root	MID5	GJHFW2E02JDP6U	Nitrogen_fixation	ref|ZP_06886595.1|	Bacteria	Proteobacteria	Alphaproteobacteria	Rhizobiales	Methylosinus trichosporium OB3b	92	1.00E-60	nitrogenase iron protein gb|EFH04962.1| nitrogenase iron protein
nifH	LN Root	MID6	GJHFW2E02HTEQ5	Nitrogen_fixation	ref|ZP_06886595.1|	Bacteria	Proteobacteria	Alphaproteobacteria	Rhizobiales	Methylosinus trichosporium OB3b	98	7.00E-42	nitrogenase iron protein gb|EFH04962.1| nitrogenase iron protein
nifH	LN Root	MID6	GJHFW2E02HTPLV	Nitrogen_fixation	ref|ZP_06886595.1|	Bacteria	Proteobacteria	Alphaproteobacteria	Rhizobiales	Methylosinus trichosporium OB3b	92	2.00E-44	nitrogenase iron protein gb|EFH04962.1| nitrogenase iron protein
nifH	LN Root	MID6	GJHFW2E02JPXE6	Nitrogen_fixation	ref|ZP_06886595.1|	Bacteria	Proteobacteria	Alphaproteobacteria	Rhizobiales	Methylosinus trichosporium OB3b	98	2.00E-58	nitrogenase iron protein gb|EFH04962.1| nitrogenase iron protein
nifH	LN Root	MID4	GJHFW2E02G1VPV	Nitrogen_fixation	ref|ZP_03510730.1|	Bacteria	Proteobacteria	Alphaproteobacteria	Rhizobiales	Rhizobium etli 8C-3	89	5.00E-32	nitrogenase reductase
nifH	LN Root	MID4	GJHFW2E02F6AQA	Nitrogen_fixation	ref|YP_001523957.1|	Bacteria	Proteobacteria	Alphaproteobacteria	Rhizobiales	Azorhizobium caulinodans ORS 571	89	4.00E-23	nitrogenase reductase
nifH	LN Root	MID4	GJHFW2E02INP95	Nitrogen_fixation	ref|YP_001523957.1|	Bacteria	Proteobacteria	Alphaproteobacteria	Rhizobiales	Azorhizobium caulinodans ORS 571	82	5.00E-48	nitrogenase reductase
nifH	LN Root	MID4	GJHFW2E02I2I95	Nitrogen_fixation	ref|YP_001415004.1|	Bacteria	Proteobacteria	Alphaproteobacteria	Rhizobiales	Xanthobacter autotrophicus Py2	93	1.00E-09	nitrogenase reductase
nifH	LN Root	MID4	GJHFW2E02F21OT	Nitrogen_fixation	ref|YP_001415004.1|	Bacteria	Proteobacteria	Alphaproteobacteria	Rhizobiales	Xanthobacter autotrophicus Py2	97	1.00E-22	nitrogenase reductase
nifH	LN Root	MID5	GJHFW2E02GAEG4	Nitrogen_fixation	ref|YP_001415004.1|	Bacteria	Proteobacteria	Alphaproteobacteria	Rhizobiales	Xanthobacter autotrophicus Py2	53	0.004	nitrogenase reductase
nifH	LN Root	MID4	GJHFW2E02HC1E4	Nitrogen_fixation	gb|ABO64220.1|	Bacteria	Proteobacteria	Betaproteobacteria	Burkholderiales	Burkholderia vietnamiensis	100	3.00E-20	nitrogenase reductase
nifH	LN Root	MID4	GJHFW2E02F3QI2	Nitrogen_fixation	gb|ABL14187.1|	Bacteria	Proteobacteria	Betaproteobacteria	Burkholderiales	Burkholderia vietnamiensis	92	1.00E-60	NifH gb|ABL14188.1|
nifH	LN Root	MID4	GJHFW2E02FF5FP	Nitrogen_fixation	ref|YP_004307391.1|	Bacteria	Firmicutes	Clostridia	Clostridiales	Clostridium lentocellum DSM 5427	98	1.00E-26	nitrogenase iron protein gb|ADZ82193.1| nitrogenase iron protein
nifH	LN Root	MID6	GJHFW2E02HK9Z0	Nitrogen_fixation	ref|YP_181872.1|	Bacteria	Chloroflexi	Dehalococcoidetes		Dehalococcoides ethenogenes 195	73	2.00E-36	nitrogenase reductase
nifH	LN Root	MID4	GJHFW2E02F3BBU	Nitrogen_fixation	ref|YP_001404303.1|	Archaea	Euryarchaeota	Methanomicrobia	Methanomicrobiales	Methanoregula boonei 6A8	98	5.00E-24	nitrogenase iron protein
nifH	LN Root	MID4	GJHFW2E02HATE7	Nitrogen_fixation	ref|YP_001404303.1|	Archaea	Euryarchaeota	Methanomicrobia	Methanomicrobiales	Methanoregula boonei 6A8	91	6.00E-52	nitrogenase iron protein
nifH	LN Root	MID5	GJHFW2E02H52H1	Nitrogen_fixation	ref|YP_001404303.1|	Archaea	Euryarchaeota	Methanomicrobia	Methanomicrobiales	Methanoregula boonei 6A8	91	4.00E-68	nitrogenase iron protein
nifH	LN Root	MID5	GJHFW2E02FOXU5	Nitrogen_fixation	ref|YP_001404303.1|	Archaea	Euryarchaeota	Methanomicrobia	Methanomicrobiales	Methanoregula boonei 6A8	73	1.00E-25	nitrogenase iron protein
nifH	LN Root	MID4	GJHFW2E02JVDQ3	Nitrogen_fixation	gb|AAQ12257.1|	Bacteria	Nitrospirae	Nitrospira (class)	Nitrospirales	Leptospirillum ferrooxidans	86	1.00E-29	nitrogenase iron protein
nifH	LN Root	MID4	GJHFW2E02GWR6U	Nitrogen_fixation	dbj|BAD93432.1|	Bacteria				uncultured bacterium	100	1.00E-42	dinitrogenase reductase
nifH	LN Root	MID4	GJHFW2E02IHCMR	Nitrogen_fixation	gb|AAV52936.1|	Bacteria				uncultured bacterium	93	2.00E-49	nitrogenase iron protein
nifH	LN Root	MID4	GJHFW2E02GEUE6	Nitrogen_fixation	dbj|BAE45514.1|	Bacteria				uncultured nitrogen-fixing bacterium	100	2.00E-37	dinitrogenase reductase
nifH	LN Root	MID4	GJHFW2E02ISJYD	Nitrogen_fixation	gb|ACI25916.1|	Bacteria				uncultured soil bacterium	99	7.00E-55	dinitrogenase reductase
nifH	LN Root	MID4	GJHFW2E02JZE5W	Nitrogen_fixation	dbj|BAE45514.1|	Bacteria				uncultured nitrogen-fixing bacterium	100	9.00E-48	dinitrogenase reductase
nifH	LN Root	MID5	GJHFW2E02FM627	Nitrogen_fixation	gb|ABQ01538.1|	Bacteria				uncultured soil bacterium	98	9.00E-35	dinitrogenase reductase
nifH	LN Root	MID5	GJHFW2E02F1SJF	Nitrogen_fixation	dbj|BAD93409.1|	Bacteria				uncultured bacterium	100	2.00E-40	dinitrogenase reductase dbj|BAD93413.1| dinitrogenase reductase
nifH	SN Root	MID2	GJHFW2E01EDOOD	Nitrogen_fixation	ref|YP_001207334.1|	Bacteria	Proteobacteria	Alphaproteobacteria	Rhizobiales	Bradyrhizobium sp. ORS278	99	3.00E-64	nitrogenase reductase emb|CAL79117.1| nitrogenase iron protein, nifH
nifH	SN Root	MID1	GJHFW2E01CZPZX	Nitrogen_fixation	ref|YP_534305.1|	Bacteria	Proteobacteria	Alphaproteobacteria	Rhizobiales	Rhodopseudomonas palustris BisB18	98	3.00E-67	nitrogenase reductase gb|ABD89986.1| nitrogenase iron protein subunit NifH
nifH	SN Root	MID1	GJHFW2E01BX4FO	Nitrogen_fixation	dbj|BAF62117.1|	Bacteria	Proteobacteria	Alphaproteobacteria	Rhizobiales	Rhodopseudomonas lichen	100	6.00E-65	reductase of nitrogenase
nifH	SN Root	MID1	GJHFW2E01CIN4H	Nitrogen_fixation	ref|YP_004677463.1|	Bacteria	Proteobacteria	Alphaproteobacteria	Rhizobiales	Hyphomicrobium sp. MC1	85	2.00E-36	nitrogenase iron protein 2 emb|CCB66897.1| Nitrogenase iron protein 2
nifH	SN Root	MID2	GJHFW2E01CBZX8	Nitrogen_fixation	ref|YP_004677463.1|	Bacteria	Proteobacteria	Alphaproteobacteria	Rhizobiales	Hyphomicrobium sp. MC1	89	1.00E-38	nitrogenase iron protein 2 emb|CCB66897.1| Nitrogenase iron protein 2
nifH	SN Root	MID2	GJHFW2E01B8UKP	Nitrogen_fixation	ref|ZP_08073552.1|	Bacteria	Proteobacteria	Alphaproteobacteria	Rhizobiales	Methylocystis sp. ATCC 49242	89	1.00E-45	nitrogenase iron protein gb|EFX98811.1| nitrogenase iron protein
nifH	SN Root	MID3	GJHFW2E01B1JPW	Nitrogen_fixation	ref|ZP_08073552.1|	Bacteria	Proteobacteria	Alphaproteobacteria	Rhizobiales	Methylocystis sp. ATCC 49242	96	5.00E-11	nitrogenase iron protein gb|EFX98811.1| nitrogenase iron protein
nifH	SN Root	MID1	GJHFW2E01DO3W0	Nitrogen_fixation	gb|ACO40460.1|	Bacteria	Proteobacteria	Alphaproteobacteria	Rhizobiales	Mesorhizobium sp. WSM3862	91	1.00E-33	NifH
nifH	SN Root	MID2	GJHFW2E01ECB07	Nitrogen_fixation	ref|YP_001314762.1|	Bacteria	Proteobacteria	Alphaproteobacteria	Rhizobiales	Sinorhizobium medicae WSM419	94	7.00E-52	nitrogenase reductase gb|ABR64829.1| nitrogenase iron protein
nifH	SN Root	MID1	GJHFW2E01BYFRE	Nitrogen_fixation	ref|YP_001167452.1|	Bacteria	Proteobacteria	Alphaproteobacteria	Rhodobacterales	Rhodobacter sphaeroides ATCC 17025	89	1.00E-28	nitrogenase reductase gb|ABP70147.1| nitrogenase iron protein subunit NifH
nifH	SN Root	MID1	GJHFW2E01BWYQW	Nitrogen_fixation	ref|YP_002299844.1|	Bacteria	Proteobacteria	Alphaproteobacteria	Rhodospirillales	Rhodospirillum centenum SW	85	9.00E-64	nitrogenase reductase gb|ACJ01032.1| NifH
nifH	SN Root	MID2	GJHFW2E01C0OZD	Nitrogen_fixation	ref|YP_001415004.1|	Bacteria	Proteobacteria	Alphaproteobacteria	Rhizobiales	Xanthobacter autotrophicus Py2	89	6.00E-33	nitrogenase reductase gb|ABS65347.1| nitrogenase iron protein
nifH	SN Root	MID1	GJHFW2E01DNNZL	Nitrogen_fixation	ref|YP_003776253.1|	Bacteria	Proteobacteria	Betaproteobacteria	Burkholderiales	Herbaspirillum seropedicae SmR1	92	1.00E-20	dinitrogenase reductase
nifH	SN Root	MID1	GJHFW2E01DIIZB	Nitrogen_fixation	ref|YP_001434094.1|	Bacteria	Chloroflexi	Chloroflexi (class)	Chloroflexales	Roseiflexus castenholzii DSM 13941	42	3.00E-16	nitrogenase iron protein
nifH	SN Root	MID1	GJHFW2E01ADDN7	Nitrogen_fixation	ref|YP_003778674.1|	Bacteria	Firmicutes	Clostridia	Clostridiales	Clostridium ljungdahlii DSM 13528	90	9.00E-72	nitrogenase iron protein gb|ADK13572.1| nitrogenase iron protein
nifH	SN Root	MID2	GJHFW2E01ED5MF	Nitrogen_fixation	ref|ZP_08194286.1|	Bacteria	Firmicutes	Clostridia	Clostridiales	Clostridium papyrosolvens DSM 2782	100	1.00E-10	nitrogenase iron protein gb|EGD46298.1| nitrogenase iron protein
nifH	SN Root	MID1	GJHFW2E01CJFEI	Nitrogen_fixation	ref|YP_004267112.1|	Bacteria	Firmicutes	Clostridia	Clostridiales	Syntrophobotulus glycolicus DSM 8271	83	2.00E-44	Mo-nitrogenase iron protein subunit NifH
nifH	SN Root	MID2	GJHFW2E01BD16N	Nitrogen_fixation	ref|YP_004267112.1|	Bacteria	Firmicutes	Clostridia	Clostridiales	Syntrophobotulus glycolicus DSM 8271	80	4.00E-28	Mo-nitrogenase iron protein subunit NifH
nifH	SN Root	MID1	GJHFW2E01D12RL	Nitrogen_fixation	ref|ZP_07326207.1|	Bacteria	Firmicutes	Clostridia	Clostridiales	Acetivibrio cellulolyticus CD2	60	5.00E-13	nitrogenase iron protein gb|EFL62576.1| nitrogenase iron protein
nifH	SN Root	MID1	GJHFW2E01BKDK1	Nitrogen_fixation	ref|ZP_07015343.1|	Bacteria	Proteobacteria	Deltaproteobacteria	Desulfovibrionales	Desulfonatronospira thiodismutans ASO3-1	80	2.00E-53	nitrogenase iron protein gb|EFI35493.1| nitrogenase iron protein
nifH	SN Root	MID1	GJHFW2E01B7EGE	Nitrogen_fixation	ref|YP_002953433.1|	Bacteria	Proteobacteria	Deltaproteobacteria	Desulfovibrionales	Desulfovibrio magneticus RS-1	92	3.00E-41	nitrogenase iron protein nitrogenase component II
nifH	SN Root	MID2	GJHFW2E01EV8AA	Nitrogen_fixation	ref|ZP_01312343.1|	Bacteria	Proteobacteria	Deltaproteobacteria	Desulfuromonadales	Desulfuromonas acetoxidans DSM 684	88	7.00E-20	nitrogenase iron protein gb|EAT15955.1| nitrogenase iron protein
nifH	SN Root	MID1	GJHFW2E01BET8N	Nitrogen_fixation	ref|YP_001380211.1|	Bacteria	Proteobacteria	Deltaproteobacteria	Myxococcales	Anaeromyxobacter sp. Fw109-5	95	3.00E-87	nitrogenase iron protein gb|ABS27227.1| nitrogenase iron protein
nifH	SN Root	MID1	GJHFW2E01EUK14	Nitrogen_fixation	ref|YP_001380211.1|	Bacteria	Proteobacteria	Deltaproteobacteria	Myxococcales	Anaeromyxobacter sp. Fw109-5	93	1.00E-40	nitrogenase iron protein gb|ABS27227.1| nitrogenase iron protein
nifH	SN Root	MID2	GJHFW2E01B6WWQ	Nitrogen_fixation	ref|YP_001380211.1|	Bacteria	Proteobacteria	Deltaproteobacteria	Myxococcales	Anaeromyxobacter sp. Fw109-5	94	1.00E-47	nitrogenase iron protein gb|ABS27227.1| nitrogenase iron protein
nifH	SN Root	MID2	GJHFW2E01EUCYI	Nitrogen_fixation	ref|YP_001380211.1|	Bacteria	Proteobacteria	Deltaproteobacteria	Myxococcales	Anaeromyxobacter sp. Fw109-5	86	1.00E-32	nitrogenase iron protein gb|ABS27227.1| nitrogenase iron protein
nifH	SN Root	MID1	GJHFW2E01CCPAI	Nitrogen_fixation	ref|YP_004369422.1|	Bacteria	Proteobacteria	Deltaproteobacteria	Syntrophobacterales	Desulfobacca acetoxidans DSM 11109	85	1.00E-43	nitrogenase iron protein gb|AEB08241.1| Nitrogenase iron protein
nifH	SN Root	MID2	GJHFW2E01DICUN	Nitrogen_fixation	ref|YP_845148.1|	Bacteria	Proteobacteria	Deltaproteobacteria	Syntrophobacterales	Syntrophobacter fumaroxidans MPOB	90	2.00E-72	nitrogenase reductase
nifH	SN Root	MID1	GJHFW2E01CIL64	Nitrogen_fixation	sp|P08624.1|NIFH2_METIV	Archaea	Euryarchaeota	Methanobacteria	Methanobacteriales	Methanobacterium ivanovii	98	6.00E-53	RecName: Full=Nitrogenase iron protein
nifH	SN Root	MID1	GJHFW2E01AT6FS	Nitrogen_fixation	sp|P51602.1|NIFH1_METIV	Archaea	Euryarchaeota	Methanobacteria	Methanobacteriales	Methanobacterium ivanovii	84	2.00E-60	RecName: Full=Nitrogenase iron protein
nifH	SN Root	MID1	GJHFW2E01DY9GQ	Nitrogen_fixation	sp|P08624.1|NIFH2_METIV	Archaea	Euryarchaeota	Methanobacteria	Methanobacteriales	Methanobacterium ivanovii	91	3.00E-71	RecName: Full=Nitrogenase iron protein
nifH	SN Root	MID1	GJHFW2E01CVHPJ	Nitrogen_fixation	sp|P51602.1|NIFH1_METIV	Archaea	Euryarchaeota	Methanobacteria	Methanobacteriales	Methanobacterium ivanovii	99	3.00E-81	RecName: Full=Nitrogenase iron protein
nifH	SN Root	MID2	GJHFW2E01D6R5D	Nitrogen_fixation	sp|P08624.1|NIFH2_METIV	Archaea	Euryarchaeota	Methanobacteria	Methanobacteriales	Methanobacterium ivanovii	94	2.00E-44	RecName: Full=Nitrogenase iron protein
nifH	SN Root	MID2	GJHFW2E01AIEN8	Nitrogen_fixation	sp|P08624.1|NIFH2_METIV	Archaea	Euryarchaeota	Methanobacteria	Methanobacteriales	Methanobacterium ivanovii	96	4.00E-84	RecName: Full=Nitrogenase iron protein
nifH	SN Root	MID2	GJHFW2E01EBU1N	Nitrogen_fixation	ref|NP_275785.1|	Archaea	Euryarchaeota	Methanobacteria	Methanobacteriales	Methanothermobacter thermautotrophicus str. Delta H	67	5.00E-32	nitrogenase reductase-like protein
nifH	SN Root	MID1	GJHFW2E01AI3WO	Nitrogen_fixation	ref|YP_001404303.1|	Archaea	Euryarchaeota	Methanomicrobia	Methanomicrobiales	Methanoregula boonei 6A8	95	9.00E-50	nitrogenase iron protein gb|ABS55660.1| nitrogenase iron protein
nifH	SN Root	MID1	GJHFW2E01EONVS	Nitrogen_fixation	ref|YP_001404303.1|	Archaea	Euryarchaeota	Methanomicrobia	Methanomicrobiales	Methanoregula boonei 6A8	94	4.00E-51	nitrogenase iron protein gb|ABS55660.1| nitrogenase iron protein
nifH	SN Root	MID1	GJHFW2E01B51XL	Nitrogen_fixation	ref|YP_001404303.1|	Archaea	Euryarchaeota	Methanomicrobia	Methanomicrobiales	Methanoregula boonei 6A8	84	1.00E-30	nitrogenase iron protein gb|ABS55660.1| nitrogenase iron protein
nifH	SN Root	MID1	GJHFW2E01CDRO5	Nitrogen_fixation	ref|YP_001404303.1|	Archaea	Euryarchaeota	Methanomicrobia	Methanomicrobiales	Methanoregula boonei 6A8	86	3.00E-49	nitrogenase iron protein gb|ABS55660.1| nitrogenase iron protein
nifH	SN Root	MID2	GJHFW2E01AT5PV	Nitrogen_fixation	ref|YP_001404303.1|	Archaea	Euryarchaeota	Methanomicrobia	Methanomicrobiales	Methanoregula boonei 6A8	87	5.00E-73	nitrogenase iron protein gb|ABS55660.1| nitrogenase iron protein
nifH	SN Root	MID2	GJHFW2E01BVDXX	Nitrogen_fixation	ref|YP_001405165.1|	Archaea	Euryarchaeota	Methanomicrobia	Methanomicrobiales	Methanoregula boonei 6A8	65	2.00E-32	nitrogenase iron protein gb|ABS56522.1| nitrogenase iron protein
nifH	SN Root	MID1	GJHFW2E01CXUBP	Nitrogen_fixation	ref|ZP_08207527.1|					Novosphingobium nitrogenifigens DSM 19370	100	3.00E-06	nitrogenase reductase gb|EGD60366.1| nitrogenase reductase
nifH	SN Root	MID2	GJHFW2E01CCAZT	Nitrogen_fixation	gb|ACO36744.1|	Bacteria				uncultured bacterium	98	1.00E-77	dinitrogenase reductase
nifH	SN Root	MID3	GJHFW2E01CC0QU	Nitrogen_fixation	gb|ABQ01499.1|	Bacteria				uncultured soil bacterium	98	6.00E-57	dinitrogenase reductase

pmo	LN Root	MID4	GJHFW2E02F403C	Particulate_methane_monooxygenas	emb|CAE48352.1|	Bacteria	Proteobacteria	Alphaproteobacteria	Rhizobiales	Methylocystis sp. SC2	73	9.00E-13	pmoA2
pmo	LN Root	MID4	GJHFW2E02GOF85	Particulate_methane_monooxygenas	emb|CAE48352.1|	Bacteria	Proteobacteria	Alphaproteobacteria	Rhizobiales	Methylocystis sp. SC2	76	1.00E-18	pmoA2
pmo	LN Root	MID4	GJHFW2E02GJ0SG	Particulate_methane_monooxygenas	emb|CAE48353.1|	Bacteria	Proteobacteria	Alphaproteobacteria	Rhizobiales	Methylocystis sp. SC2	72	9.00E-42	pmoB2
pmo	LN Root	MID4	GJHFW2E02GP0PD	Particulate_methane_monooxygenas	emb|CAE48353.1|	Bacteria	Proteobacteria	Alphaproteobacteria	Rhizobiales	Methylocystis sp. SC2	74	8.00E-43	pmoB2
pmo	LN Root	MID4	GJHFW2E02H4NNO	Particulate_methane_monooxygenas	emb|CAE48353.1|	Bacteria	Proteobacteria	Alphaproteobacteria	Rhizobiales	Methylocystis sp. SC2	78	5.00E-40	pmoB2
pmo	LN Root	MID4	GJHFW2E02IO3NM	Particulate_methane_monooxygenas	emb|CAE48353.1|	Bacteria	Proteobacteria	Alphaproteobacteria	Rhizobiales	Methylocystis sp. SC2	74	2.00E-61	pmoB2
pmo	LN Root	MID4	GJHFW2E02IPUP7	Particulate_methane_monooxygenas	gb|AAF37897.1|	Bacteria	Proteobacteria	Alphaproteobacteria	Rhizobiales	Methylocystis sp. M	97	6.00E-12	PmoB
pmo	LN Root	MID4	GJHFW2E02H2HHH	Particulate_methane_monooxygenas	emb|CAE48351.1|	Bacteria	Proteobacteria	Alphaproteobacteria	Rhizobiales	Methylocystis sp. SC2	87	1.00E-43	pmoC2
pmo	LN Root	MID4	GJHFW2E02JUK5W	Particulate_methane_monooxygenas	emb|CAE48351.1|	Bacteria	Proteobacteria	Alphaproteobacteria	Rhizobiales	Methylocystis sp. SC2	77	2.00E-61	pmoC2
pmo	LN Root	MID4	GJHFW2E02G8BR2	Particulate_methane_monooxygenas	ref|ZP_08073627.1|	Bacteria	Proteobacteria	Alphaproteobacteria	Rhizobiales	Methylocystis sp. ATCC 49242	68	1.00E-17	Ammonia monooxygenase/methane monooxygenase subunit C
pmo	LN Root	MID4	GJHFW2E02GEFYG	Particulate_methane_monooxygenas	emb|CAE48351.1|	Bacteria	Proteobacteria	Alphaproteobacteria	Rhizobiales	Methylocystis sp. SC2	91	1.00E-47	pmoC2
pmo	LN Root	MID4	GJHFW2E02F4LBS	Particulate_methane_monooxygenas	emb|CAE47799.1|	Bacteria	Proteobacteria	Alphaproteobacteria	Rhizobiales	Methylocystis sp. SC2	71	3.00E-40	Particulate Methane Monooxigenase subunit C
pmo	LN Root	MID4	GJHFW2E02FI0XP	Particulate_methane_monooxygenas	ref|ZP_08074599.1|	Bacteria	Proteobacteria	Alphaproteobacteria	Rhizobiales	Methylocystis sp. ATCC 49242	90	6.00E-59	methane monooxygenase/ammonia monooxygenase, subunit C
pmo	LN Root	MID4	GJHFW2E02FSXP9	Particulate_methane_monooxygenas	emb|CAE48351.1|	Bacteria	Proteobacteria	Alphaproteobacteria	Rhizobiales	Methylocystis sp. SC2	92	3.00E-73	pmoC2
pmo	LN Root	MID4	GJHFW2E02G4GOB	Particulate_methane_monooxygenas	emb|CAE48351.1|	Bacteria	Proteobacteria	Alphaproteobacteria	Rhizobiales	Methylocystis sp. SC2	92	1.00E-71	pmoC2
pmo	LN Root	MID4	GJHFW2E02GAWW8	Particulate_methane_monooxygenas	ref|ZP_08071271.1|	Bacteria	Proteobacteria	Alphaproteobacteria	Rhizobiales	Methylocystis sp. ATCC 49242	95	3.00E-06	methane monooxygenase/ammonia monooxygenase, subunit C
pmo	LN Root	MID4	GJHFW2E02GCJ7D	Particulate_methane_monooxygenas	emb|CAE48351.1|	Bacteria	Proteobacteria	Alphaproteobacteria	Rhizobiales	Methylocystis sp. SC2	89	3.00E-39	pmoC2
pmo	LN Root	MID4	GJHFW2E02GHYOK	Particulate_methane_monooxygenas	emb|CAE48351.1|	Bacteria	Proteobacteria	Alphaproteobacteria	Rhizobiales	Methylocystis sp. SC2	71	1.00E-13	pmoC2
pmo	LN Root	MID4	GJHFW2E02GQ4XL	Particulate_methane_monooxygenas	emb|CAE48351.1|	Bacteria	Proteobacteria	Alphaproteobacteria	Rhizobiales	Methylocystis sp. SC2	90	6.00E-56	pmoC2
pmo	LN Root	MID4	GJHFW2E02HCLA4	Particulate_methane_monooxygenas	emb|CAE48351.1|	Bacteria	Proteobacteria	Alphaproteobacteria	Rhizobiales	Methylocystis sp. SC2	73	4.00E-19	pmoC2
pmo	LN Root	MID4	GJHFW2E02HGRH1	Particulate_methane_monooxygenas	emb|CAE48351.1|	Bacteria	Proteobacteria	Alphaproteobacteria	Rhizobiales	Methylocystis sp. SC2	91	6.00E-80	pmoC2
pmo	LN Root	MID4	GJHFW2E02HI4VK	Particulate_methane_monooxygenas	ref|ZP_08071271.1|	Bacteria	Proteobacteria	Alphaproteobacteria	Rhizobiales	Methylocystis sp. ATCC 49242	95	2.00E-64	methane monooxygenase/ammonia monooxygenase, subunit C
pmo	LN Root	MID4	GJHFW2E02HTZ6A	Particulate_methane_monooxygenas	emb|CAE47799.1|	Bacteria	Proteobacteria	Alphaproteobacteria	Rhizobiales	Methylocystis sp. SC2	70	2.00E-44	Particulate Methane Monooxigenase subunit C
pmo	LN Root	MID4	GJHFW2E02I44XW	Particulate_methane_monooxygenas	gb|ABD57884.1|	Bacteria	Proteobacteria	Alphaproteobacteria	Rhizobiales	uncultured Methylocystis sp. GSC357	97	2.00E-34	particulate methane monooxygenase subunit C
pmo	LN Root	MID4	GJHFW2E02I8713	Particulate_methane_monooxygenas	gb|AAF37896.1|	Bacteria	Proteobacteria	Alphaproteobacteria	Rhizobiales	Methylocystis sp. M	96	1.00E-40	PmoC
pmo	LN Root	MID4	GJHFW2E02IZ1ZR	Particulate_methane_monooxygenas	emb|CAE48351.1|	Bacteria	Proteobacteria	Alphaproteobacteria	Rhizobiales	Methylocystis sp. SC2	87	3.00E-44	pmoC2
pmo	LN Root	MID4	GJHFW2E02J17C9	Particulate_methane_monooxygenas	emb|CAE48351.1|	Bacteria	Proteobacteria	Alphaproteobacteria	Rhizobiales	Methylocystis sp. SC2	63	7.00E-08	pmoC2
pmo	LN Root	MID4	GJHFW2E02JR4PE	Particulate_methane_monooxygenas	emb|CAE48351.1|	Bacteria	Proteobacteria	Alphaproteobacteria	Rhizobiales	Methylocystis sp. SC2	80	4.00E-61	pmoC2
pmo	LN Root	MID5	GJHFW2E02FEQ21	Particulate_methane_monooxygenas	emb|CAE48352.1|	Bacteria	Proteobacteria	Alphaproteobacteria	Rhizobiales	Methylocystis sp. SC2	83	6.00E-27	pmoA2
pmo	LN Root	MID5	GJHFW2E02F11WS	Particulate_methane_monooxygenas	emb|CAE48353.1|	Bacteria	Proteobacteria	Alphaproteobacteria	Rhizobiales	Methylocystis sp. SC2	90	2.00E-16	pmoB2
pmo	LN Root	MID5	GJHFW2E02JXKB6	Particulate_methane_monooxygenas	emb|CAE48353.1|	Bacteria	Proteobacteria	Alphaproteobacteria	Rhizobiales	Methylocystis sp. SC2	85	2.00E-32	pmoB2
pmo	LN Root	MID5	GJHFW2E02FYDYR	Particulate_methane_monooxygenas	emb|CAE48351.1|	Bacteria	Proteobacteria	Alphaproteobacteria	Rhizobiales	Methylocystis sp. SC2	74	8.00E-25	pmoC2
pmo	LN Root	MID5	GJHFW2E02G49XC	Particulate_methane_monooxygenas	emb|CAE48351.1|	Bacteria	Proteobacteria	Alphaproteobacteria	Rhizobiales	Methylocystis sp. SC2	78	2.00E-35	pmoC2
pmo	LN Root	MID5	GJHFW2E02GVPJC	Particulate_methane_monooxygenas	emb|CAE48351.1|	Bacteria	Proteobacteria	Alphaproteobacteria	Rhizobiales	Methylocystis sp. SC2	60	6.00E-06	pmoC2
pmo	LN Root	MID5	GJHFW2E02I78GV	Particulate_methane_monooxygenas	emb|CAE48351.1|	Bacteria	Proteobacteria	Alphaproteobacteria	Rhizobiales	Methylocystis sp. SC2	83	6.00E-81	pmoC2
pmo	LN Root	MID5	GJHFW2E02IF1XA	Particulate_methane_monooxygenas	emb|CAE47799.1|	Bacteria	Proteobacteria	Alphaproteobacteria	Rhizobiales	Methylocystis sp. SC2	71	4.00E-37	Particulate Methane Monooxigenase subunit C
pmo	LN Root	MID5	GJHFW2E02IMRWN	Particulate_methane_monooxygenas	emb|CAE48351.1|	Bacteria	Proteobacteria	Alphaproteobacteria	Rhizobiales	Methylocystis sp. SC2	92	2.00E-61	pmoC2
pmo	LN Root	MID6	GJHFW2E02I1W9U	Particulate_methane_monooxygenas	ref|ZP_08071273.1|	Bacteria	Proteobacteria	Alphaproteobacteria	Rhizobiales	Methylocystis sp. ATCC 49242	91	2.00E-12	methane monooxygenase/ammonia monooxygenase, subunit B
pmo	LN Root	MID6	GJHFW2E02GO53N	Particulate_methane_monooxygenas	gb|AAF37896.1|	Bacteria	Proteobacteria	Alphaproteobacteria	Rhizobiales	Methylocystis sp. M	100	8.00E-37	PmoC
pmo	LN Root	MID4	GJHFW2E02F4NQD	Particulate_methane_monooxygenas	ref|ZP_06890678.1|	Bacteria	Proteobacteria	Alphaproteobacteria	Rhizobiales	Methylosinus trichosporium OB3b	89	7.00E-51	methane monooxygenase/ammonia monooxygenase, subunit B
pmo	LN Root	MID4	GJHFW2E02FS7UG	Particulate_methane_monooxygenas	pdb|3CHX|A	Bacteria	Proteobacteria	Alphaproteobacteria	Rhizobiales		93	4.00E-26	Chain A, b Particulate Methane Monooxygenase (Pmmo)
pmo	LN Root	MID4	GJHFW2E02FZOD1	Particulate_methane_monooxygenas	ref|ZP_06890678.1|	Bacteria	Proteobacteria	Alphaproteobacteria	Rhizobiales	Methylosinus trichosporium OB3b	86	1.00E-70	methane monooxygenase/ammonia monooxygenase, subunit B gb|AAF37894.1| PmoB
pmo	LN Root	MID4	GJHFW2E02GM29P	Particulate_methane_monooxygenas	pdb|3CHX|A	Bacteria	Proteobacteria	Alphaproteobacteria	Rhizobiales		91	4.00E-60	Chain A, Particulate Methane Monooxygenase (Pmmo)
pmo	LN Root	MID4	GJHFW2E02H16DF	Particulate_methane_monooxygenas	ref|ZP_06890678.1|	Bacteria	Proteobacteria	Alphaproteobacteria	Rhizobiales	Methylosinus trichosporium OB3b	62	2.00E-10	methane monooxygenase/ammonia monooxygenase, subunit B
pmo	LN Root	MID4	GJHFW2E02H1DQI	Particulate_methane_monooxygenas	ref|ZP_06890678.1|	Bacteria	Proteobacteria	Alphaproteobacteria	Rhizobiales	Methylosinus trichosporium OB3b	75	2.00E-28	methane monooxygenase/ammonia monooxygenase, subunit B
pmo	LN Root	MID4	GJHFW2E02H2NWY	Particulate_methane_monooxygenas	ref|ZP_06890678.1|	Bacteria	Proteobacteria	Alphaproteobacteria	Rhizobiales	Methylosinus trichosporium OB3b	58	2.00E-06	methane monooxygenase/ammonia monooxygenase, subunit B
pmo	LN Root	MID4	GJHFW2E02H3YCR	Particulate_methane_monooxygenas	ref|ZP_06890678.1|	Bacteria	Proteobacteria	Alphaproteobacteria	Rhizobiales	Methylosinus trichosporium OB3b	76	4.00E-36	methane monooxygenase/ammonia monooxygenase, subunit B
pmo	LN Root	MID4	GJHFW2E02H5EZG	Particulate_methane_monooxygenas	ref|ZP_06890678.1|	Bacteria	Proteobacteria	Alphaproteobacteria	Rhizobiales	Methylosinus trichosporium OB3b	89	1.00E-46	methane monooxygenase/ammonia monooxygenase, subunit B
pmo	LN Root	MID4	GJHFW2E02HGEBO	Particulate_methane_monooxygenas	ref|ZP_06890678.1|	Bacteria	Proteobacteria	Alphaproteobacteria	Rhizobiales	Methylosinus trichosporium OB3b	96	2.00E-54	methane monooxygenase/ammonia monooxygenase, subunit B
pmo	LN Root	MID4	GJHFW2E02HGESA	Particulate_methane_monooxygenas	ref|ZP_06890678.1|	Bacteria	Proteobacteria	Alphaproteobacteria	Rhizobiales	Methylosinus trichosporium OB3b	95	1.00E-68	methane monooxygenase/ammonia monooxygenase, subunit B
pmo	LN Root	MID4	GJHFW2E02HV3O4	Particulate_methane_monooxygenas	pdb|3CHX|A	Bacteria	Proteobacteria	Alphaproteobacteria	Rhizobiales		95	3.00E-47	Chain A, Particulate Methane Monooxygenase (Pmmo)
pmo	LN Root	MID4	GJHFW2E02HV6JR	Particulate_methane_monooxygenas	ref|ZP_06890678.1|	Bacteria	Proteobacteria	Alphaproteobacteria	Rhizobiales	Methylosinus trichosporium OB3b	86	6.00E-57	methane monooxygenase/ammonia monooxygenase, subunit B gb|AAF37894.1| PmoB
pmo	LN Root	MID4	GJHFW2E02IG53R	Particulate_methane_monooxygenas	ref|ZP_06890678.1|	Bacteria	Proteobacteria	Alphaproteobacteria	Rhizobiales	Methylosinus trichosporium OB3b	90	3.00E-72	methane monooxygenase/ammonia monooxygenase, subunit B gb|AAF37894.1| PmoB
pmo	LN Root	MID4	GJHFW2E02IHMEH	Particulate_methane_monooxygenas	ref|ZP_06890678.1|	Bacteria	Proteobacteria	Alphaproteobacteria	Rhizobiales	Methylosinus trichosporium OB3b	90	4.00E-53	methane monooxygenase/ammonia monooxygenase, subunit B gb|AAF37894.1| PmoB
pmo	LN Root	MID4	GJHFW2E02IIX6U	Particulate_methane_monooxygenas	ref|ZP_06890678.1|	Bacteria	Proteobacteria	Alphaproteobacteria	Rhizobiales	Methylosinus trichosporium OB3b	88	6.00E-39	methane monooxygenase/ammonia monooxygenase, subunit B gb|AAF37894.1| PmoB
pmo	LN Root	MID4	GJHFW2E02IO2OB	Particulate_methane_monooxygenas	pdb|3CHX|A	Bacteria	Proteobacteria	Alphaproteobacteria	Rhizobiales		90	5.00E-73	Chain A, Particulate Methane Monooxygenase (Pmmo)
pmo	LN Root	MID4	GJHFW2E02IOAYZ	Particulate_methane_monooxygenas	ref|ZP_06890678.1|	Bacteria	Proteobacteria	Alphaproteobacteria	Rhizobiales	Methylosinus trichosporium OB3b	90	1.00E-62	methane monooxygenase/ammonia monooxygenase, subunit B
pmo	LN Root	MID4	GJHFW2E02ISL66	Particulate_methane_monooxygenas	pdb|3CHX|A	Bacteria	Proteobacteria	Alphaproteobacteria	Rhizobiales		91	1.00E-83	Chain A, Particulate Methane Monooxygenase (Pmmo)
pmo	LN Root	MID4	GJHFW2E02ITP4H	Particulate_methane_monooxygenas	ref|ZP_06890679.1|	Bacteria	Proteobacteria	Alphaproteobacteria	Rhizobiales	Methylosinus trichosporium OB3b	97	2.00E-19	methane monooxygenase/ammonia monooxygenase, subunit A
pmo	LN Root	MID4	GJHFW2E02FNCMZ	Particulate_methane_monooxygenas	pdb|3CHX|B	Bacteria	Proteobacteria	Alphaproteobacteria	Rhizobiales	Methylosinus trichosporium OB3b	58	0.004	Chain B, Particulate Methane Monooxygenase (Pmmo)
pmo	LN Root	MID4	GJHFW2E02GC6KT	Particulate_methane_monooxygenas	ref|ZP_06890680.1|	Bacteria	Proteobacteria	Alphaproteobacteria	Rhizobiales	Methylosinus trichosporium OB3b	71	3.00E-26	methane monooxygenase/ammonia monooxygenase, subunit C
pmo	LN Root	MID4	GJHFW2E02GUH06	Particulate_methane_monooxygenas	ref|ZP_06890680.1|	Bacteria	Proteobacteria	Alphaproteobacteria	Rhizobiales	Methylosinus trichosporium OB3b	66	9.00E-40	methane monooxygenase/ammonia monooxygenase, subunit C
pmo	LN Root	MID4	GJHFW2E02HEELM	Particulate_methane_monooxygenas	ref|ZP_06890680.1|	Bacteria	Proteobacteria	Alphaproteobacteria	Rhizobiales	Methylosinus trichosporium OB3b	93	1.00E-41	methane monooxygenase/ammonia monooxygenase, subunit C
pmo	LN Root	MID4	GJHFW2E02ILTRN	Particulate_methane_monooxygenas	ref|ZP_06890680.1|	Bacteria	Proteobacteria	Alphaproteobacteria	Rhizobiales	Methylosinus trichosporium OB3b	73	2.00E-43	methane monooxygenase/ammonia monooxygenase, subunit C
pmo	LN Root	MID4	GJHFW2E02IS9NI	Particulate_methane_monooxygenas	ref|ZP_06890680.1|	Bacteria	Proteobacteria	Alphaproteobacteria	Rhizobiales	Methylosinus trichosporium OB3b	78	1.00E-44	methane monooxygenase/ammonia monooxygenase, subunit C
pmo	LN Root	MID4	GJHFW2E02JZT7N	Particulate_methane_monooxygenas	ref|ZP_06890680.1|	Bacteria	Proteobacteria	Alphaproteobacteria	Rhizobiales	Methylosinus trichosporium OB3b	80	4.00E-43	methane monooxygenase/ammonia monooxygenase, subunit C
pmo	LN Root	MID4	GJHFW2E02FIES9	Particulate_methane_monooxygenas	ref|ZP_06888910.1|	Bacteria	Proteobacteria	Alphaproteobacteria	Rhizobiales	Methylosinus trichosporium OB3b	91	0.002	methane monooxygenase/ammonia monooxygenase, subunit C
pmo	LN Root	MID4	GJHFW2E02FLJOI	Particulate_methane_monooxygenas	ref|ZP_06890680.1|	Bacteria	Proteobacteria	Alphaproteobacteria	Rhizobiales	Methylosinus trichosporium OB3b	92	1.00E-77	methane monooxygenase/ammonia monooxygenase, subunit C
pmo	LN Root	MID4	GJHFW2E02GIISG	Particulate_methane_monooxygenas	ref|ZP_06890680.1|	Bacteria	Proteobacteria	Alphaproteobacteria	Rhizobiales	Methylosinus trichosporium OB3b	91	1.00E-69	methane monooxygenase/ammonia monooxygenase, subunit C

pmo	LN Root	MID4	GJHFW2E02GRFG0	Particulate_methane_monooxygenas	ref|ZP_06888910.1|	Bacteria	Proteobacteria	Alphaproteobacteria	Rhizobiales	Methylosinus trichosporium OB3b	96	8.00E-28	methane monooxygenase/ammonia monooxygenase, subunit C
pmo	LN Root	MID4	GJHFW2E02GSFFO	Particulate_methane_monooxygenas	ref|ZP_06890680.1|	Bacteria	Proteobacteria	Alphaproteobacteria	Rhizobiales	Methylosinus trichosporium OB3b	93	6.00E-81	methane monooxygenase/ammonia monooxygenase, subunit C
pmo	LN Root	MID4	GJHFW2E02H07WT	Particulate_methane_monooxygenas	ref|ZP_06888910.1|	Bacteria	Proteobacteria	Alphaproteobacteria	Rhizobiales	Methylosinus trichosporium OB3b	96	3.00E-21	methane monooxygenase/ammonia monooxygenase, subunit C
pmo	LN Root	MID4	GJHFW2E02I0OZ1	Particulate_methane_monooxygenas	ref|ZP_06890680.1|	Bacteria	Proteobacteria	Alphaproteobacteria	Rhizobiales	Methylosinus trichosporium OB3b	91	2.00E-43	methane monooxygenase/ammonia monooxygenase, subunit C
pmo	LN Root	MID4	GJHFW2E02IZH0F	Particulate_methane_monooxygenas	ref|ZP_06888910.1|	Bacteria	Proteobacteria	Alphaproteobacteria	Rhizobiales	Methylosinus trichosporium OB3b	97	7.00E-79	methane monooxygenase/ammonia monooxygenase, subunit C
pmo	LN Root	MID4	GJHFW2E02J0B75	Particulate_methane_monooxygenas	ref|ZP_06890680.1|	Bacteria	Proteobacteria	Alphaproteobacteria	Rhizobiales	Methylosinus trichosporium OB3b	94	5.00E-31	methane monooxygenase/ammonia monooxygenase, subunit C
pmo	LN Root	MID4	GJHFW2E02J246D	Particulate_methane_monooxygenas	ref|ZP_06888910.1|	Bacteria	Proteobacteria	Alphaproteobacteria	Rhizobiales	Methylosinus trichosporium OB3b	80	1.00E-32	methane monooxygenase/ammonia monooxygenase, subunit C
pmo	LN Root	MID4	GJHFW2E02JC6C7	Particulate_methane_monooxygenas	ref|ZP_06888910.1|	Bacteria	Proteobacteria	Alphaproteobacteria	Rhizobiales	Methylosinus trichosporium OB3b	97	1.00E-52	methane monooxygenase/ammonia monooxygenase, subunit C
pmo	LN Root	MID4	GJHFW2E02JCGMA	Particulate_methane_monooxygenas	ref|ZP_06890680.1|	Bacteria	Proteobacteria	Alphaproteobacteria	Rhizobiales	Methylosinus trichosporium OB3b	92	2.00E-23	methane monooxygenase/ammonia monooxygenase, subunit C
pmo	LN Root	MID4	GJHFW2E02JJ3RP	Particulate_methane_monooxygenas	ref|ZP_06888910.1|	Bacteria	Proteobacteria	Alphaproteobacteria	Rhizobiales	Methylosinus trichosporium OB3b	96	3.00E-21	methane monooxygenase/ammonia monooxygenase, subunit C
pmo	LN Root	MID4	GJHFW2E02F0SF9	Particulate_methane_monooxygenas	ref|ZP_06890680.1|	Bacteria	Proteobacteria	Alphaproteobacteria	Rhizobiales	Methylosinus trichosporium OB3b	78	1.00E-30	methane monooxygenase/ammonia monooxygenase, subunit C
pmo	LN Root	MID4	GJHFW2E02F4ZD0	Particulate_methane_monooxygenas	ref|ZP_06890680.1|	Bacteria	Proteobacteria	Alphaproteobacteria	Rhizobiales	Methylosinus trichosporium OB3b	73	2.00E-17	methane monooxygenase/ammonia monooxygenase, subunit C
pmo	LN Root	MID4	GJHFW2E02F610V	Particulate_methane_monooxygenas	ref|ZP_06890680.1|	Bacteria	Proteobacteria	Alphaproteobacteria	Rhizobiales	Methylosinus trichosporium OB3b	73	7.00E-32	methane monooxygenase/ammonia monooxygenase, subunit C
pmo	LN Root	MID4	GJHFW2E02GCYI6	Particulate_methane_monooxygenas	ref|ZP_06890680.1|	Bacteria	Proteobacteria	Alphaproteobacteria	Rhizobiales	Methylosinus trichosporium OB3b	73	2.00E-28	methane monooxygenase/ammonia monooxygenase, subunit C
pmo	LN Root	MID4	GJHFW2E02GILA0	Particulate_methane_monooxygenas	ref|ZP_06888910.1|	Bacteria	Proteobacteria	Alphaproteobacteria	Rhizobiales	Methylosinus trichosporium OB3b	59	6.00E-14	methane monooxygenase/ammonia monooxygenase, subunit C
pmo	LN Root	MID4	GJHFW2E02GJIEW	Particulate_methane_monooxygenas	ref|ZP_06890680.1|	Bacteria	Proteobacteria	Alphaproteobacteria	Rhizobiales	Methylosinus trichosporium OB3b	82	9.00E-73	methane monooxygenase/ammonia monooxygenase, subunit C
pmo	LN Root	MID4	GJHFW2E02HCU8P	Particulate_methane_monooxygenas	ref|ZP_06890680.1|	Bacteria	Proteobacteria	Alphaproteobacteria	Rhizobiales	Methylosinus trichosporium OB3b	73	2.00E-18	methane monooxygenase/ammonia monooxygenase, subunit C
pmo	LN Root	MID4	GJHFW2E02HLGUA	Particulate_methane_monooxygenas	ref|ZP_06890680.1|	Bacteria	Proteobacteria	Alphaproteobacteria	Rhizobiales	Methylosinus trichosporium OB3b	89	3.00E-75	methane monooxygenase/ammonia monooxygenase, subunit C
pmo	LN Root	MID4	GJHFW2E02I12QJ	Particulate_methane_monooxygenas	ref|ZP_06888910.1|	Bacteria	Proteobacteria	Alphaproteobacteria	Rhizobiales	Methylosinus trichosporium OB3b	67	3.00E-14	methane monooxygenase/ammonia monooxygenase, subunit C
pmo	LN Root	MID4	GJHFW2E02IF97T	Particulate_methane_monooxygenas	ref|ZP_06890680.1|	Bacteria	Proteobacteria	Alphaproteobacteria	Rhizobiales	Methylosinus trichosporium OB3b	74	2.00E-29	methane monooxygenase/ammonia monooxygenase, subunit C
pmo	LN Root	MID4	GJHFW2E02IHHEU	Particulate_methane_monooxygenas	ref|ZP_06890680.1|	Bacteria	Proteobacteria	Alphaproteobacteria	Rhizobiales	Methylosinus trichosporium OB3b	73	5.00E-30	methane monooxygenase/ammonia monooxygenase, subunit C
pmo	LN Root	MID4	GJHFW2E02IOLD0	Particulate_methane_monooxygenas	ref|ZP_06890680.1|	Bacteria	Proteobacteria	Alphaproteobacteria	Rhizobiales	Methylosinus trichosporium OB3b	79	1.00E-43	methane monooxygenase/ammonia monooxygenase, subunit C
pmo	LN Root	MID4	GJHFW2E02J2YV3	Particulate_methane_monooxygenas	ref|ZP_06888910.1|	Bacteria	Proteobacteria	Alphaproteobacteria	Rhizobiales	Methylosinus trichosporium OB3b	80	9.00E-34	methane monooxygenase/ammonia monooxygenase, subunit C
pmo	LN Root	MID4	GJHFW2E02JVWH6	Particulate_methane_monooxygenas	ref|ZP_06888910.1|	Bacteria	Proteobacteria	Alphaproteobacteria	Rhizobiales	Methylosinus trichosporium OB3b	90	4.00E-61	methane monooxygenase/ammonia monooxygenase, subunit C
pmo	LN Root	MID5	GJHFW2E02GJX6Z	Particulate_methane_monooxygenas	ref|ZP_06890679.1|	Bacteria	Proteobacteria	Alphaproteobacteria	Rhizobiales	Methylosinus trichosporium OB3b	97	4.00E-20	methane monooxygenase/ammonia monooxygenase, subunit A
pmo	LN Root	MID5	GJHFW2E02G99Q6	Particulate_methane_monooxygenas	ref|ZP_06890678.1|	Bacteria	Proteobacteria	Alphaproteobacteria	Rhizobiales	Methylosinus trichosporium OB3b	97	5.00E-45	methane monooxygenase/ammonia monooxygenase, subunit B
pmo	LN Root	MID5	GJHFW2E02GY5TY	Particulate_methane_monooxygenas	ref|ZP_06890678.1|	Bacteria	Proteobacteria	Alphaproteobacteria	Rhizobiales	Methylosinus trichosporium OB3b	83	2.00E-23	methane monooxygenase/ammonia monooxygenase, subunit B
pmo	LN Root	MID5	GJHFW2E02JO0ZA	Particulate_methane_monooxygenas	ref|ZP_06890678.1|	Bacteria	Proteobacteria	Alphaproteobacteria	Rhizobiales	Methylosinus trichosporium OB3b	88	1.00E-73	methane monooxygenase/ammonia monooxygenase, subunit B
pmo	LN Root	MID5	GJHFW2E02JZ44P	Particulate_methane_monooxygenas	ref|ZP_06890678.1|	Bacteria	Proteobacteria	Alphaproteobacteria	Rhizobiales	Methylosinus trichosporium OB3b	100	2.00E-26	methane monooxygenase/ammonia monooxygenase, subunit B
pmo	LN Root	MID5	GJHFW2E02G9AM7	Particulate_methane_monooxygenas	ref|ZP_06890680.1|	Bacteria	Proteobacteria	Alphaproteobacteria	Rhizobiales	Methylosinus trichosporium OB3b	92	6.00E-14	methane monooxygenase/ammonia monooxygenase, subunit C
pmo	LN Root	MID5	GJHFW2E02H88KY	Particulate_methane_monooxygenas	ref|ZP_06888910.1|	Bacteria	Proteobacteria	Alphaproteobacteria	Rhizobiales	Methylosinus trichosporium OB3b	95	2.00E-17	methane monooxygenase/ammonia monooxygenase, subunit C
pmo	LN Root	MID5	GJHFW2E02HJCLY	Particulate_methane_monooxygenas	ref|ZP_06888910.1|	Bacteria	Proteobacteria	Alphaproteobacteria	Rhizobiales	Methylosinus trichosporium OB3b	96	8.00E-30	methane monooxygenase/ammonia monooxygenase, subunit C
pmo	LN Root	MID5	GJHFW2E02HQ0Q7	Particulate_methane_monooxygenas	ref|ZP_06890680.1|	Bacteria	Proteobacteria	Alphaproteobacteria	Rhizobiales	Methylosinus trichosporium OB3b	94	5.00E-80	methane monooxygenase/ammonia monooxygenase, subunit C
pmo	LN Root	MID5	GJHFW2E02I9QZO	Particulate_methane_monooxygenas	ref|ZP_06890680.1|	Bacteria	Proteobacteria	Alphaproteobacteria	Rhizobiales	Methylosinus trichosporium OB3b	94	1.00E-32	methane monooxygenase/ammonia monooxygenase, subunit C
pmo	LN Root	MID5	GJHFW2E02GM7W7	Particulate_methane_monooxygenas	ref|ZP_06890680.1|	Bacteria	Proteobacteria	Alphaproteobacteria	Rhizobiales	Methylosinus trichosporium OB3b	72	3.00E-15	methane monooxygenase/ammonia monooxygenase, subunit C
pmo	LN Root	MID5	GJHFW2E02HE53V	Particulate_methane_monooxygenas	ref|ZP_06890680.1|	Bacteria	Proteobacteria	Alphaproteobacteria	Rhizobiales	Methylosinus trichosporium OB3b	74	2.00E-29	methane monooxygenase/ammonia monooxygenase, subunit C
pmo	LN Root	MID5	GJHFW2E02HK7CN	Particulate_methane_monooxygenas	ref|ZP_06888910.1|	Bacteria	Proteobacteria	Alphaproteobacteria	Rhizobiales	Methylosinus trichosporium OB3b	86	6.00E-20	methane monooxygenase/ammonia monooxygenase, subunit C
pmo	LN Root	MID6	GJHFW2E02HO5LM	Particulate_methane_monooxygenas	pdb|3CHX|A	Bacteria	Proteobacteria	Alphaproteobacteria	Rhizobiales		88	1.00E-55	Chain A, Particulate Methane Monooxygenase (Pmmo)
pmo	LN Root	MID6	GJHFW2E02FU13K	Particulate_methane_monooxygenas	ref|ZP_06888910.1|	Bacteria	Proteobacteria	Alphaproteobacteria	Rhizobiales	Methylosinus trichosporium OB3b	82	4.00E-32	methane monooxygenase/ammonia monooxygenase, subunit C
pmo	LN Root	MID6	GJHFW2E02GLKTZ	Particulate_methane_monooxygenas	ref|ZP_06888910.1|	Bacteria	Proteobacteria	Alphaproteobacteria	Rhizobiales	Methylosinus trichosporium OB3b	96	2.00E-39	methane monooxygenase/ammonia monooxygenase, subunit C
pmo	LN Root	MID6	GJHFW2E02GSIVI	Particulate_methane_monooxygenas	ref|ZP_06888910.1|	Bacteria	Proteobacteria	Alphaproteobacteria	Rhizobiales	Methylosinus trichosporium OB3b	95	3.00E-19	methane monooxygenase/ammonia monooxygenase, subunit C
pmo	LN Root	MID6	GJHFW2E02F0WDP	Particulate_methane_monooxygenas	ref|ZP_06888910.1|	Bacteria	Proteobacteria	Alphaproteobacteria	Rhizobiales	Methylosinus trichosporium OB3b	88	1.00E-42	methane monooxygenase/ammonia monooxygenase, subunit C
pmo	LN Root	MID6	GJHFW2E02FUNWS	Particulate_methane_monooxygenas	ref|ZP_06888910.1|	Bacteria	Proteobacteria	Alphaproteobacteria	Rhizobiales	Methylosinus trichosporium OB3b	79	4.00E-54	methane monooxygenase/ammonia monooxygenase, subunit C
pmo	LN Root	MID6	GJHFW2E02GD6PT	Particulate_methane_monooxygenas	ref|ZP_06890680.1|	Bacteria	Proteobacteria	Alphaproteobacteria	Rhizobiales	Methylosinus trichosporium OB3b	94	8.00E-78	methane monooxygenase/ammonia monooxygenase, subunit C
pmo	LN Root	MID6	GJHFW2E02IB119	Particulate_methane_monooxygenas	ref|ZP_06890680.1|	Bacteria	Proteobacteria	Alphaproteobacteria	Rhizobiales	Methylosinus trichosporium OB3b	85	4.00E-43	methane monooxygenase/ammonia monooxygenase, subunit C
pmo	LN Root	MID6	GJHFW2E02JA68N	Particulate_methane_monooxygenas	ref|ZP_06890680.1|	Bacteria	Proteobacteria	Alphaproteobacteria	Rhizobiales	Methylosinus trichosporium OB3b	96	3.00E-57	methane monooxygenase/ammonia monooxygenase, subunit C

pmo	SN Root	MID1	GJHFW2E01C2CDJ	Particulate_methane_monooxygenas	ref|ZP_08071273.1|	Bacteria	Proteobacteria	Alphaproteobacteria	Rhizobiales	Methylocystis sp. ATCC 49242	97	2.00E-67	methane monooxygenase/ammonia monooxygenase, subunit B
pmo	SN Root	MID1	GJHFW2E01AK8GN	Particulate_methane_monooxygenas	ref|ZP_08074599.1|	Bacteria	Proteobacteria	Alphaproteobacteria	Rhizobiales	Methylocystis sp. ATCC 49242	93	3.00E-85	methane monooxygenase/ammonia monooxygenase, subunit C
pmo	SN Root	MID1	GJHFW2E01B96HW	Particulate_methane_monooxygenas	ref|ZP_08071271.1|	Bacteria	Proteobacteria	Alphaproteobacteria	Rhizobiales	Methylocystis sp. ATCC 49242	91	2.00E-70	methane monooxygenase/ammonia monooxygenase, subunit C
pmo	SN Root	MID1	GJHFW2E01C31R0	Particulate_methane_monooxygenas	ref|ZP_08070737.1|	Bacteria	Proteobacteria	Alphaproteobacteria	Rhizobiales	Methylocystis sp. ATCC 49242	78	3.00E-28	methane monooxygenase/ammonia monooxygenase, subunit C
pmo	SN Root	MID2	GJHFW2E01A8IIC	Particulate_methane_monooxygenas	ref|ZP_08071273.1|	Bacteria	Proteobacteria	Alphaproteobacteria	Rhizobiales	Methylocystis sp. ATCC 49242	90	1.00E-51	methane monooxygenase/ammonia monooxygenase, subunit B
pmo	SN Root	MID2	GJHFW2E01AO7XK	Particulate_methane_monooxygenas	emb|CAE48353.1|	Bacteria	Proteobacteria	Alphaproteobacteria	Rhizobiales	Methylocystis sp. SC2	82	2.00E-39	pmoB2
pmo	SN Root	MID2	GJHFW2E01AVM6J	Particulate_methane_monooxygenas	emb|CAE48353.1|	Bacteria	Proteobacteria	Alphaproteobacteria	Rhizobiales	Methylocystis sp. SC2	69	4.00E-50	pmoB2
pmo	SN Root	MID2	GJHFW2E01BUS5P	Particulate_methane_monooxygenas	emb|CAE48353.1|	Bacteria	Proteobacteria	Alphaproteobacteria	Rhizobiales	Methylocystis sp. SC2	82	3.00E-47	pmoB2
pmo	SN Root	MID2	GJHFW2E01D0XCU	Particulate_methane_monooxygenas	gb|AAF37897.1|	Bacteria	Proteobacteria	Alphaproteobacteria	Rhizobiales	Methylocystis sp. M	85	1.00E-71	PmoB
pmo	SN Root	MID2	GJHFW2E01D3V6G	Particulate_methane_monooxygenas	gb|AAF37897.1|	Bacteria	Proteobacteria	Alphaproteobacteria	Rhizobiales	Methylocystis sp. M	97	1.00E-31	PmoB
pmo	SN Root	MID2	GJHFW2E01BOZ03	Particulate_methane_monooxygenas	emb|CAE48351.1|	Bacteria	Proteobacteria	Alphaproteobacteria	Rhizobiales	Methylocystis sp. SC2	81	6.00E-76	pmoC2
pmo	SN Root	MID2	GJHFW2E01A3873	Particulate_methane_monooxygenas	emb|CAE48351.1|	Bacteria	Proteobacteria	Alphaproteobacteria	Rhizobiales	Methylocystis sp. SC2	47	6.00E-34	pmoC2
pmo	SN Root	MID3	GJHFW2E01CZI0X	Particulate_methane_monooxygenas	gb|AAC45295.2|	Bacteria	Proteobacteria	Alphaproteobacteria	Rhizobiales	Methylocystis sp. M	100	4.00E-24	PmoA
pmo	SN Root	MID3	GJHFW2E01C4JIW	Particulate_methane_monooxygenas	gb|AAF37896.1|	Bacteria	Proteobacteria	Alphaproteobacteria	Rhizobiales	Methylocystis sp. M	97	4.00E-45	PmoC
pmo	SN Root	MID3	GJHFW2E01A1S8Z	Particulate_methane_monooxygenas	ref|ZP_08074599.1|	Bacteria	Proteobacteria	Alphaproteobacteria	Rhizobiales	Methylocystis sp. ATCC 49242	94	6.00E-59	methane monooxygenase/ammonia monooxygenase, subunit
pmo	SN Root	MID1	GJHFW2E01BQMU8	Particulate_methane_monooxygenas	ref|ZP_06890680.1|	Bacteria	Proteobacteria	Alphaproteobacteria	Rhizobiales	Methylosinus trichosporium OB3b	95	4.00E-37	methane monooxygenase/ammonia monooxygenase, subunit
pmo	SN Root	MID1	GJHFW2E01A9PAT	Particulate_methane_monooxygenas	ref|ZP_06890680.1|	Bacteria	Proteobacteria	Alphaproteobacteria	Rhizobiales	Methylosinus trichosporium OB3b	62	2.00E-23	methane monooxygenase/ammonia monooxygenase, subunit C
pmo	SN Root	MID2	GJHFW2E01DISEL	Particulate_methane_monooxygenas	ref|ZP_06890679.1|	Bacteria	Proteobacteria	Alphaproteobacteria	Rhizobiales	Methylosinus trichosporium OB3b	98	1.00E-26	methane monooxygenase/ammonia monooxygenase, subunit A
pmo	SN Root	MID2	GJHFW2E01DU5AR	Particulate_methane_monooxygenas	ref|ZP_06890679.1|	Bacteria	Proteobacteria	Alphaproteobacteria	Rhizobiales	Methylosinus trichosporium OB3b	95	9.00E-32	methane monooxygenase/ammonia monooxygenase, subunit A
pmo	SN Root	MID2	GJHFW2E01EJ7EQ	Particulate_methane_monooxygenas	ref|ZP_06890680.1|	Bacteria	Proteobacteria	Alphaproteobacteria	Rhizobiales	Methylosinus trichosporium OB3b	93	2.00E-16	methane monooxygenase/ammonia monooxygenase, subunit C
pmo	SN Root	MID2	GJHFW2E01A3W5O	Particulate_methane_monooxygenas	ref|ZP_06888910.1|	Bacteria	Proteobacteria	Alphaproteobacteria	Rhizobiales	Methylosinus trichosporium OB3b	81	4.00E-51	methane monooxygenase/ammonia monooxygenase, subunit C
pmo	SN Root	MID2	GJHFW2E01B1NVU	Particulate_methane_monooxygenas	ref|ZP_06890680.1|	Bacteria	Proteobacteria	Alphaproteobacteria	Rhizobiales	Methylosinus trichosporium OB3b	89	2.00E-74	methane monooxygenase/ammonia monooxygenase, subunit C
pmo	SN Root	MID2	GJHFW2E01BDWZ1	Particulate_methane_monooxygenas	ref|ZP_06890680.1|	Bacteria	Proteobacteria	Alphaproteobacteria	Rhizobiales	Methylosinus trichosporium OB3b	82	9.00E-50	methane monooxygenase/ammonia monooxygenase, subunit C
pmo	SN Root	MID2	GJHFW2E01CEMX3	Particulate_methane_monooxygenas	ref|ZP_06888910.1|	Bacteria	Proteobacteria	Alphaproteobacteria	Rhizobiales	Methylosinus trichosporium OB3b	41	2.00E-14	methane monooxygenase/ammonia monooxygenase, subunit C
pmo	SN Root	MID2	GJHFW2E01CLUJR	Particulate_methane_monooxygenas	ref|ZP_06888910.1|	Bacteria	Proteobacteria	Alphaproteobacteria	Rhizobiales	Methylosinus trichosporium OB3b	88	2.00E-20	methane monooxygenase/ammonia monooxygenase, subunit C
pmo	SN Root	MID3	GJHFW2E01EEWMJ	Particulate_methane_monooxygenas	pdb|3CHX|A	Bacteria	Proteobacteria	Alphaproteobacteria	Rhizobiales		93	7.00E-85	Chain A, Particulate Methane Monooxygenase (Pmmo)
pmo	SN Root	MID2	GJHFW2E01A9KZR	Particulate_methane_monooxygenas	ref|YP_115247.1|	Bacteria	Proteobacteria	Gammaproteobacteria	Methylococcales	Methylococcus capsulatus str. Bath	55	6.00E-49	methane monooxygenase subunit B

mmo	LN Root	MID4	GJHFW2E02F5NGU	Soluble_methane_monooxygenase_(s	ref|YP_002361595.1|	Bacteria	Proteobacteria	Alphaproteobacteria	Rhizobiales	Methylocella silvestris BL2	67	5.00E-17	monooxygenase component MmoB/DmpM
mmo	LN Root	MID4	GJHFW2E02GTVPB	Soluble_methane_monooxygenase_(s	ref|YP_002361595.1|	Bacteria	Proteobacteria	Alphaproteobacteria	Rhizobiales	Methylocella silvestris BL2	87	1.00E-39	monooxygenase component MmoB/DmpM
mmo	LN Root	MID4	GJHFW2E02I7JD4	Soluble_methane_monooxygenase_(s	ref|YP_002361595.1|	Bacteria	Proteobacteria	Alphaproteobacteria	Rhizobiales	Methylocella silvestris BL2	88	1.00E-58	monooxygenase component MmoB/DmpM
mmo	LN Root	MID4	GJHFW2E02IEJ5J	Soluble_methane_monooxygenase_(s	ref|YP_002361595.1|	Bacteria	Proteobacteria	Alphaproteobacteria	Rhizobiales	Methylocella silvestris BL2	87	1.00E-37	monooxygenase component MmoB/DmpM
mmo	LN Root	MID4	GJHFW2E02F5FSW	Soluble_methane_monooxygenase_(s	ref|YP_002361593.1|	Bacteria	Proteobacteria	Alphaproteobacteria	Rhizobiales	Methylocella silvestris BL2	90	1.00E-13	Methane monooxygenase
mmo	LN Root	MID4	GJHFW2E02HS655	Soluble_methane_monooxygenase_(s	ref|YP_002361593.1|	Bacteria	Proteobacteria	Alphaproteobacteria	Rhizobiales	Methylocella silvestris BL2	85	1.00E-25	Methane monooxygenase
mmo	LN Root	MID4	GJHFW2E02I8QND	Soluble_methane_monooxygenase_(s	ref|YP_002361593.1|	Bacteria	Proteobacteria	Alphaproteobacteria	Rhizobiales	Methylocella silvestris BL2	86	7.00E-63	Methane monooxygenase
mmo	LN Root	MID4	GJHFW2E02IMMYG	Soluble_methane_monooxygenase_(s	ref|YP_002361593.1|	Bacteria	Proteobacteria	Alphaproteobacteria	Rhizobiales	Methylocella silvestris BL2	95	4.00E-67	Methane monooxygenase
mmo	LN Root	MID4	GJHFW2E02F3XUK	Soluble_methane_monooxygenase_(s	ref|YP_002361594.1|	Bacteria	Proteobacteria	Alphaproteobacteria	Rhizobiales	Methylocella silvestris BL2	89	2.00E-68	Methane monooxygenase
mmo	LN Root	MID4	GJHFW2E02HDES4	Soluble_methane_monooxygenase_(s	ref|YP_002361594.1|	Bacteria	Proteobacteria	Alphaproteobacteria	Rhizobiales	Methylocella silvestris BL2	85	2.00E-72	Methane monooxygenase
mmo	LN Root	MID4	GJHFW2E02HKC14	Soluble_methane_monooxygenase_(s	ref|YP_002361594.1|	Bacteria	Proteobacteria	Alphaproteobacteria	Rhizobiales	Methylocella silvestris BL2	88	5.00E-59	Methane monooxygenase
mmo	LN Root	MID4	GJHFW2E02IJF1O	Soluble_methane_monooxygenase_(s	ref|YP_002361594.1|	Bacteria	Proteobacteria	Alphaproteobacteria	Rhizobiales	Methylocella silvestris BL2	91	4.00E-61	Methane monooxygenase
mmo	LN Root	MID5	GJHFW2E02HEKFY	Soluble_methane_monooxygenase_(s	ref|YP_002361594.1|	Bacteria	Proteobacteria	Alphaproteobacteria	Rhizobiales	Methylocella silvestris BL2	65	1.00E-35	Methane monooxygenase
mmo	LN Root	MID5	GJHFW2E02H07RO	Soluble_methane_monooxygenase_(s	ref|YP_002361598.1|	Bacteria	Proteobacteria	Alphaproteobacteria	Rhizobiales	Methylocella silvestris BL2	75	1.00E-48	protein C of soluble methane monooxygenase
mmo	LN Root	MID5	GJHFW2E02HLDG5	Soluble_methane_monooxygenase_(s	ref|YP_002361598.1|	Bacteria	Proteobacteria	Alphaproteobacteria	Rhizobiales	Methylocella silvestris BL2	67	1.00E-11	protein C of soluble methane monooxygenase
mmo	LN Root	MID6	GJHFW2E02FMO4M	Soluble_methane_monooxygenase_(s	ref|YP_002361594.1|	Bacteria	Proteobacteria	Alphaproteobacteria	Rhizobiales	Methylocella silvestris BL2	86	7.00E-82	Methane monooxygenase
mmo	LN Root	MID6	GJHFW2E02GPLMH	Soluble_methane_monooxygenase_(s	ref|YP_002361594.1|	Bacteria	Proteobacteria	Alphaproteobacteria	Rhizobiales	Methylocella silvestris BL2	89	5.00E-80	Methane monooxygenase
mmo	LN Root	MID6	GJHFW2E02JEOTZ	Soluble_methane_monooxygenase_(s	ref|YP_002361594.1|	Bacteria	Proteobacteria	Alphaproteobacteria	Rhizobiales	Methylocella silvestris BL2	90	1.00E-82	Methane monooxygenase
mmo	LN Root	MID6	GJHFW2E02IHZSB	Soluble_methane_monooxygenase_(s	ref|YP_002361598.1|	Bacteria	Proteobacteria	Alphaproteobacteria	Rhizobiales	Methylocella silvestris BL2	74	2.00E-62	protein C of soluble methane monooxygenase
mmo	LN Root	MID5	GJHFW2E02JAHT6	Soluble_methane_monooxygenase_(s	emb|CBW47543.1|	Bacteria	Proteobacteria	Alphaproteobacteria	Rhizobiales	Beijerinckiaceae bacterium AR4	98	5.00E-91	soluble methane monooxygenase alpha subunit
mmo	LN Root	MID4	GJHFW2E02F5EH2	Soluble_methane_monooxygenase_(s	ref|NP_770317.1|	Bacteria	Proteobacteria	Alphaproteobacteria	Rhizobiales	Bradyrhizobium japonicum USDA 110	100	5.00E-50	monooxygenase component dbj|BAC48942.1| blr3677
mmo	LN Root	MID4	GJHFW2E02GRENE	Soluble_methane_monooxygenase_(s	ref|YP_001206321.1|	Bacteria	Proteobacteria	Alphaproteobacteria	Rhizobiales	Bradyrhizobium sp. ORS278	98	3.00E-84	putative methane/phenol/toluene monooxygenase subunit alpha
mmo	LN Root	MID4	GJHFW2E02HHPOM	Soluble_methane_monooxygenase_(s	ref|YP_001206321.1|	Bacteria	Proteobacteria	Alphaproteobacteria	Rhizobiales	Bradyrhizobium sp. ORS278	91	2.00E-77	putative methane/phenol/toluene monooxygenase subunit alpha
mmo	LN Root	MID4	GJHFW2E02FZK7D	Soluble_methane_monooxygenase_(s	ref|NP_770320.1|	Bacteria	Proteobacteria	Alphaproteobacteria	Rhizobiales	Bradyrhizobium japonicum USDA 110	93	8.00E-59	hypothetical protein blr3680 dbj|BAC48945.1| blr3680
mmo	LN Root	MID5	GJHFW2E02JK60L	Soluble_methane_monooxygenase_(s	ref|YP_001239624.1|	Bacteria	Proteobacteria	Alphaproteobacteria	Rhizobiales	Bradyrhizobium sp. BTAi1	96	4.00E-67	putative methane/phenol/toluene monooxygenase subunit beta
mmo	LN Root	MID5	GJHFW2E02GH7SM	Soluble_methane_monooxygenase_(s	ref|YP_001206321.1|	Bacteria	Proteobacteria	Alphaproteobacteria	Rhizobiales	Bradyrhizobium sp. ORS278	99	4.00E-88	putative methane/phenol/toluene monooxygenase subunit alpha
mmo	LN Root	MID5	GJHFW2E02JANBU	Soluble_methane_monooxygenase_(s	ref|YP_001206321.1|	Bacteria	Proteobacteria	Alphaproteobacteria	Rhizobiales	Bradyrhizobium sp. ORS278	100	1.00E-69	putative methane/phenol/toluene monooxygenase subunit alpha
mmo	LN Root	MID6	GJHFW2E02IJ3NB	Soluble_methane_monooxygenase_(s	ref|YP_001239622.1|	Bacteria	Proteobacteria	Alphaproteobacteria	Rhizobiales	Bradyrhizobium sp. BTAi1	97	1.00E-55	putative methane/phenol/toluene monooxygenase subunit alpha
mmo	LN Root	MID4	GJHFW2E02IXXM5	Soluble_methane_monooxygenase_(s	gb|AAC45290.1|	Bacteria	Proteobacteria	Alphaproteobacteria	Rhizobiales	Methylocystis sp. M	98	4.00E-51	soluble methane monooxygenase protein A beta subunit
mmo	LN Root	MID4	GJHFW2E02F4VZZ	Soluble_methane_monooxygenase_(s	gb|AAC45289.1|	Bacteria	Proteobacteria	Alphaproteobacteria	Rhizobiales	Methylocystis sp. M	100	3.00E-36	soluble methane monooxygenase protein A alpha subunit
mmo	LN Root	MID4	GJHFW2E02F7HG4	Soluble_methane_monooxygenase_(s	gb|AAC45289.1|	Bacteria	Proteobacteria	Alphaproteobacteria	Rhizobiales	Methylocystis sp. M	99	2.00E-71	soluble methane monooxygenase protein A alpha subunit
mmo	LN Root	MID4	GJHFW2E02GTJBD	Soluble_methane_monooxygenase_(s	gb|AAC45289.1|	Bacteria	Proteobacteria	Alphaproteobacteria	Rhizobiales	Methylocystis sp. M	100	1.00E-29	soluble methane monooxygenase protein A alpha subunit
mmo	LN Root	MID4	GJHFW2E02JYNYL	Soluble_methane_monooxygenase_(s	gb|AAF01268.1|AF153282_1	Bacteria	Proteobacteria	Alphaproteobacteria	Rhizobiales	Methylocystis sp. WI14	97	1.00E-61	MmoX
mmo	LN Root	MID4	GJHFW2E02JYV8N	Soluble_methane_monooxygenase_(s	gb|AAC45289.1|	Bacteria	Proteobacteria	Alphaproteobacteria	Rhizobiales	Methylocystis sp. M	100	2.00E-53	soluble methane monooxygenase protein A alpha subunit
mmo	LN Root	MID4	GJHFW2E02F32VU	Soluble_methane_monooxygenase_(s	gb|AAC45290.1|	Bacteria	Proteobacteria	Alphaproteobacteria	Rhizobiales	Methylocystis sp. M	100	1.00E-27	soluble methane monooxygenase protein A beta subunit
mmo	LN Root	MID4	GJHFW2E02FTEVP	Soluble_methane_monooxygenase_(s	gb|AAC45290.1|	Bacteria	Proteobacteria	Alphaproteobacteria	Rhizobiales	Methylocystis sp. M	100	7.00E-63	soluble methane monooxygenase protein A beta subunit
mmo	LN Root	MID4	GJHFW2E02G6LNC	Soluble_methane_monooxygenase_(s	gb|AAC45290.1|	Bacteria	Proteobacteria	Alphaproteobacteria	Rhizobiales	Methylocystis sp. M	99	6.00E-89	soluble methane monooxygenase protein A beta subunit
mmo	LN Root	MID4	GJHFW2E02GGW49	Soluble_methane_monooxygenase_(s	gb|AAC45290.1|	Bacteria	Proteobacteria	Alphaproteobacteria	Rhizobiales	Methylocystis sp. M	97	4.00E-52	soluble methane monooxygenase protein A beta subunit
mmo	LN Root	MID4	GJHFW2E02GOBEW	Soluble_methane_monooxygenase_(s	gb|AAF01269.1|AF153282_2	Bacteria	Proteobacteria	Alphaproteobacteria	Rhizobiales	Methylocystis sp. WI14	79	9.00E-42	MmoY
mmo	LN Root	MID4	GJHFW2E02GYJJY	Soluble_methane_monooxygenase_(s	gb|AAC45290.1|	Bacteria	Proteobacteria	Alphaproteobacteria	Rhizobiales	Methylocystis sp. M	95	1.00E-71	soluble methane monooxygenase protein A beta subunit
mmo	LN Root	MID4	GJHFW2E02HKBSP	Soluble_methane_monooxygenase_(s	gb|AAC45290.1|	Bacteria	Proteobacteria	Alphaproteobacteria	Rhizobiales	Methylocystis sp. M	71	3.00E-35	soluble methane monooxygenase protein A beta subunit
mmo	LN Root	MID4	GJHFW2E02HL2KW	Soluble_methane_monooxygenase_(s	gb|AAC45290.1|	Bacteria	Proteobacteria	Alphaproteobacteria	Rhizobiales	Methylocystis sp. M	95	2.00E-70	soluble methane monooxygenase protein A beta subunit
mmo	LN Root	MID4	GJHFW2E02HMKJF	Soluble_methane_monooxygenase_(s	gb|AAC45290.1|	Bacteria	Proteobacteria	Alphaproteobacteria	Rhizobiales	Methylocystis sp. M	85	1.00E-44	soluble methane monooxygenase protein A beta subunit
mmo	LN Root	MID4	GJHFW2E02IAIWR	Soluble_methane_monooxygenase_(s	gb|AAC45290.1|	Bacteria	Proteobacteria	Alphaproteobacteria	Rhizobiales	Methylocystis sp. M	98	1.00E-83	soluble methane monooxygenase protein A beta subunit
mmo	LN Root	MID4	GJHFW2E02INSPK	Soluble_methane_monooxygenase_(s	gb|AAC45290.1|	Bacteria	Proteobacteria	Alphaproteobacteria	Rhizobiales	Methylocystis sp. M	95	1.00E-71	soluble methane monooxygenase protein A beta subunit
mmo	LN Root	MID4	GJHFW2E02HBCCM	Soluble_methane_monooxygenase_(s	gb|AAC45293.1|	Bacteria	Proteobacteria	Alphaproteobacteria	Rhizobiales	Methylocystis sp. M	94	1.00E-39	orfY
mmo	LN Root	MID4	GJHFW2E02I3PYR	Soluble_methane_monooxygenase_(s	gb|AAC45294.1|	Bacteria	Proteobacteria	Alphaproteobacteria	Rhizobiales	Methylocystis sp. M	98	3.00E-84	soluble methane monooxygenase protein C
mmo	LN Root	MID4	GJHFW2E02I88Z0	Soluble_methane_monooxygenase_(s	gb|AAF01273.1|AF153282_6	Bacteria	Proteobacteria	Alphaproteobacteria	Rhizobiales	Methylocystis sp. WI14	97	7.00E-48	MmoC
mmo	LN Root	MID4	GJHFW2E02JXH56	Soluble_methane_monooxygenase_(s	gb|AAF01273.1|AF153282_6	Bacteria	Proteobacteria	Alphaproteobacteria	Rhizobiales	Methylocystis sp. WI14	75	5.00E-32	MmoC
mmo	LN Root	MID5	GJHFW2E02G1JUE	Soluble_methane_monooxygenase_(s	gb|AAF01268.1|AF153282_1	Bacteria	Proteobacteria	Alphaproteobacteria	Rhizobiales	Methylocystis sp. WI14	93	6.00E-18	MmoX
mmo	LN Root	MID5	GJHFW2E02GQZDI	Soluble_methane_monooxygenase_(s	gb|AAC45289.1|	Bacteria	Proteobacteria	Alphaproteobacteria	Rhizobiales	Methylocystis sp. M	100	7.00E-55	soluble methane monooxygenase protein A alpha subunit
mmo	LN Root	MID5	GJHFW2E02HZTN4	Soluble_methane_monooxygenase_(s	gb|AAC45290.1|	Bacteria	Proteobacteria	Alphaproteobacteria	Rhizobiales	Methylocystis sp. M	98	9.00E-69	soluble methane monooxygenase protein A beta subunit
mmo	LN Root	MID6	GJHFW2E02JF87A	Soluble_methane_monooxygenase_(s	gb|AAC45289.1|	Bacteria	Proteobacteria	Alphaproteobacteria	Rhizobiales	Methylocystis sp. M	100	5.00E-58	soluble methane monooxygenase protein A alpha subunit
mmo	LN Root	MID6	GJHFW2E02H28PU	Soluble_methane_monooxygenase_(s	gb|AAF01269.1|AF153282_2	Bacteria	Proteobacteria	Alphaproteobacteria	Rhizobiales	Methylocystis sp. WI14	92	2.00E-66	MmoY
mmo	LN Root	MID6	GJHFW2E02HB90K	Soluble_methane_monooxygenase_(s	gb|AAF01269.1|AF153282_2	Bacteria	Proteobacteria	Alphaproteobacteria	Rhizobiales	Methylocystis sp. WI14	96	5.00E-85	MmoY
mmo	LN Root	MID6	GJHFW2E02IMXAY	Soluble_methane_monooxygenase_(s	gb|AAC45294.1|	Bacteria	Proteobacteria	Alphaproteobacteria	Rhizobiales	Methylocystis sp. M	94	4.00E-51	soluble methane monooxygenase protein C
mmo	LN Root	MID4	GJHFW2E02FR6ZU	Soluble_methane_monooxygenase_(s	gb|AAZ81969.1|	Bacteria	Proteobacteria	Alphaproteobacteria	Rhizobiales	Methylomonas sp. GYJ3	86	3.00E-43	protein A beta subunit of soluble methane monooxygenase
mmo	LN Root	MID4	GJHFW2E02GBVLB	Soluble_methane_monooxygenase_(s	gb|ABD46895.1|	Bacteria	Proteobacteria	Alphaproteobacteria	Rhizobiales	Methylosinus sporium	100	1.00E-40	MmoZ
mmo	LN Root	MID4	GJHFW2E02GIA6N	Soluble_methane_monooxygenase_(s	gb|ABD46895.1|	Bacteria	Proteobacteria	Alphaproteobacteria	Rhizobiales	Methylosinus sporium	100	2.00E-27	MmoZ
mmo	LN Root	MID4	GJHFW2E02GRWLP	Soluble_methane_monooxygenase_(s	gb|ABD46894.1|	Bacteria	Proteobacteria	Alphaproteobacteria	Rhizobiales	Methylosinus sporium	88	9.00E-40	MmoB
mmo	LN Root	MID4	GJHFW2E02HE9IK	Soluble_methane_monooxygenase_(s	gb|ABD46894.1|	Bacteria	Proteobacteria	Alphaproteobacteria	Rhizobiales	Methylosinus sporium	89	4.00E-55	MmoB
mmo	LN Root	MID4	GJHFW2E02HK9WG	Soluble_methane_monooxygenase_(s	gb|ABD46894.1|	Bacteria	Proteobacteria	Alphaproteobacteria	Rhizobiales	Methylosinus sporium	90	1.00E-53	MmoB
mmo	LN Root	MID4	GJHFW2E02I519H	Soluble_methane_monooxygenase_(s	gb|ABD46893.1|	Bacteria	Proteobacteria	Alphaproteobacteria	Rhizobiales	Methylosinus sporium	92	1.00E-44	MmoY
mmo	LN Root	MID4	GJHFW2E02I9HH5	Soluble_methane_monooxygenase_(s	gb|ABD46894.1|	Bacteria	Proteobacteria	Alphaproteobacteria	Rhizobiales	Methylosinus sporium	85	2.00E-38	MmoB
mmo	LN Root	MID4	GJHFW2E02IBI6G	Soluble_methane_monooxygenase_(s	gb|ABD46894.1|	Bacteria	Proteobacteria	Alphaproteobacteria	Rhizobiales	Methylosinus sporium	83	7.00E-32	MmoB
mmo	LN Root	MID4	GJHFW2E02JZW6Y	Soluble_methane_monooxygenase_(s	gb|ABD46894.1|	Bacteria	Proteobacteria	Alphaproteobacteria	Rhizobiales	Methylosinus sporium	80	3.00E-42	MmoB
mmo	LN Root	MID4	GJHFW2E02FLZ1W	Soluble_methane_monooxygenase_(s	gb|ABD46892.1|	Bacteria	Proteobacteria	Alphaproteobacteria	Rhizobiales	Methylosinus sporium	100	4.00E-55	MmoX1
mmo	LN Root	MID4	GJHFW2E02G3407	Soluble_methane_monooxygenase_(s	gb|ABD46898.1|	Bacteria	Proteobacteria	Alphaproteobacteria	Rhizobiales	Methylosinus sporium	100	2.00E-63	MmoX2
mmo	LN Root	MID4	GJHFW2E02GEO2M	Soluble_methane_monooxygenase_(s	gb|ABD46898.1|	Bacteria	Proteobacteria	Alphaproteobacteria	Rhizobiales	Methylosinus sporium	96	7.00E-08	MmoX2
mmo	LN Root	MID4	GJHFW2E02GLU4U	Soluble_methane_monooxygenase_(s	gb|ABD46892.1|	Bacteria	Proteobacteria	Alphaproteobacteria	Rhizobiales	Methylosinus sporium	100	1.00E-16	MmoX1
mmo	LN Root	MID4	GJHFW2E02GROXN	Soluble_methane_monooxygenase_(s	gb|ABD46898.1|	Bacteria	Proteobacteria	Alphaproteobacteria	Rhizobiales	Methylosinus sporium	100	7.00E-24	MmoX2
mmo	LN Root	MID4	GJHFW2E02H0HPQ	Soluble_methane_monooxygenase_(s	gb|ABD46898.1|	Bacteria	Proteobacteria	Alphaproteobacteria	Rhizobiales	Methylosinus sporium	98	2.00E-74	MmoX2
mmo	LN Root	MID4	GJHFW2E02I0PYL	Soluble_methane_monooxygenase_(s	gb|ABD46892.1|	Bacteria	Proteobacteria	Alphaproteobacteria	Rhizobiales	Methylosinus sporium	97	4.00E-49	MmoX1
mmo	LN Root	MID4	GJHFW2E02I1NHA	Soluble_methane_monooxygenase_(s	gb|ABD46898.1|	Bacteria	Proteobacteria	Alphaproteobacteria	Rhizobiales	Methylosinus sporium	97	2.00E-13	MmoX2
mmo	LN Root	MID4	GJHFW2E02IIZUX	Soluble_methane_monooxygenase_(s	gb|ABD46892.1|	Bacteria	Proteobacteria	Alphaproteobacteria	Rhizobiales	Methylosinus sporium	100	6.00E-47	MmoX1
mmo	LN Root	MID4	GJHFW2E02ILMAT	Soluble_methane_monooxygenase_(s	gb|ABD46898.1|	Bacteria	Proteobacteria	Alphaproteobacteria	Rhizobiales	Methylosinus sporium	100	7.00E-53	MmoX2
mmo	LN Root	MID4	GJHFW2E02GX7BR	Soluble_methane_monooxygenase_(s	gb|ABD46893.1|	Bacteria	Proteobacteria	Alphaproteobacteria	Rhizobiales	Methylosinus sporium	99	9.00E-77	MmoY
mmo	LN Root	MID4	GJHFW2E02HE8J0	Soluble_methane_monooxygenase_(s	gb|ABD46897.1|	Bacteria	Proteobacteria	Alphaproteobacteria	Rhizobiales	Methylosinus sporium	97	4.00E-71	MmoC
mmo	LN Root	MID4	GJHFW2E02HGLNX	Soluble_methane_monooxygenase_(s	gb|ABD46896.1|	Bacteria	Proteobacteria	Alphaproteobacteria	Rhizobiales	Methylosinus sporium	100	2.00E-21	MmoD
mmo	LN Root	MID4	GJHFW2E02HKS25	Soluble_methane_monooxygenase_(s	gb|ABD46897.1|	Bacteria	Proteobacteria	Alphaproteobacteria	Rhizobiales	Methylosinus sporium	100	8.00E-59	MmoC
mmo	LN Root	MID4	GJHFW2E02IYQVS	Soluble_methane_monooxygenase_(s	gb|ABD46897.1|	Bacteria	Proteobacteria	Alphaproteobacteria	Rhizobiales	Methylosinus sporium	99	3.00E-63	MmoC
mmo	LN Root	MID4	GJHFW2E02J0NHM	Soluble_methane_monooxygenase_(s	gb|ABD46897.1|	Bacteria	Proteobacteria	Alphaproteobacteria	Rhizobiales	Methylosinus sporium	99	6.00E-76	MmoC
mmo	LN Root	MID4	GJHFW2E02JV6RH	Soluble_methane_monooxygenase_(s	gb|AAZ81973.1|	Bacteria	Proteobacteria	Alphaproteobacteria	Rhizobiales	Methylosinus trichosporium	98	2.00E-30	protein C of soluble methane monooxygenase
mmo	LN Root	MID5	GJHFW2E02H72UH	Soluble_methane_monooxygenase_(s	gb|ABD46894.1|	Bacteria	Proteobacteria	Alphaproteobacteria	Rhizobiales	Methylosinus sporium	88	4.00E-45	MmoB
mmo	LN Root	MID5	GJHFW2E02HHTXE	Soluble_methane_monooxygenase_(s	gb|AAZ81969.1|	Bacteria	Proteobacteria	Alphaproteobacteria	Rhizobiales	Methylomonas sp. GYJ3	92	2.00E-22	protein A beta subunit of soluble methane monooxygenase
mmo	LN Root	MID5	GJHFW2E02G5S82	Soluble_methane_monooxygenase_(s	gb|ABD46898.1|	Bacteria	Proteobacteria	Alphaproteobacteria	Rhizobiales	Methylosinus sporium	100	1.00E-25	MmoX2
mmo	LN Root	MID5	GJHFW2E02GLJZ1	Soluble_methane_monooxygenase_(s	gb|ABD46892.1|	Bacteria	Proteobacteria	Alphaproteobacteria	Rhizobiales	Methylosinus sporium	100	1.00E-84	MmoX1
mmo	LN Root	MID5	GJHFW2E02GO11H	Soluble_methane_monooxygenase_(s	gb|ABD46892.1|	Bacteria	Proteobacteria	Alphaproteobacteria	Rhizobiales	Methylosinus sporium	100	5.00E-45	MmoX1
mmo	LN Root	MID5	GJHFW2E02I9XJ9	Soluble_methane_monooxygenase_(s	gb|ABD46898.1|	Bacteria	Proteobacteria	Alphaproteobacteria	Rhizobiales	Methylosinus sporium	100	1.00E-67	MmoX2
mmo	LN Root	MID5	GJHFW2E02F23S1	Soluble_methane_monooxygenase_(s	gb|ABD46897.1|	Bacteria	Proteobacteria	Alphaproteobacteria	Rhizobiales	Methylosinus sporium	100	5.00E-40	MmoC
mmo	LN Root	MID5	GJHFW2E02FVMEK	Soluble_methane_monooxygenase_(s	gb|ABD46896.1|	Bacteria	Proteobacteria	Alphaproteobacteria	Rhizobiales	Methylosinus sporium	76	1.00E-43	MmoD
mmo	LN Root	MID5	GJHFW2E02HPZEK	Soluble_methane_monooxygenase_(s	ref|ZP_06887024.1|	Bacteria	Proteobacteria	Alphaproteobacteria	Rhizobiales	Methylosinus trichosporium OB3b	98	8.00E-44	protein C of soluble methane monooxygenase
mmo	LN Root	MID6	GJHFW2E02FGUUK	Soluble_methane_monooxygenase_(s	gb|ABD46894.1|	Bacteria	Proteobacteria	Alphaproteobacteria	Rhizobiales	Methylosinus sporium	84	1.00E-34	MmoB

mmo	LN Root	MID6	GJHFW2E02HAI1Y	Soluble_methane_monooxygenase_(s	gb|ABD46894.1|	Bacteria	Proteobacteria	Alphaproteobacteria	Rhizobiales	Methylosinus sporium	91	3.00E-63	MmoB
mmo	LN Root	MID6	GJHFW2E02G0LR4	Soluble_methane_monooxygenase_(s	gb|ABD46892.1|	Bacteria	Proteobacteria	Alphaproteobacteria	Rhizobiales	Methylosinus sporium	94	2.00E-24	MmoX1
mmo	LN Root	MID6	GJHFW2E02H0Z7W	Soluble_methane_monooxygenase_(s	gb|ABD46892.1|	Bacteria	Proteobacteria	Alphaproteobacteria	Rhizobiales	Methylosinus sporium	100	4.00E-86	MmoX1
mmo	LN Root	MID6	GJHFW2E02IZHV9	Soluble_methane_monooxygenase_(s	gb|ABD46898.1|	Bacteria	Proteobacteria	Alphaproteobacteria	Rhizobiales	Methylosinus sporium	97	1.00E-85	MmoX2
mmo	LN Root	MID4	GJHFW2E02HQ48I	Soluble_methane_monooxygenase_(s	ref|ZP_05780578.1|	Bacteria	Proteobacteria	Alphaproteobacteria	Rhodobacterales	Citreicella sp. SE45	91	2.00E-69	methane monooxygenase
mmo	LN Root	MID4	GJHFW2E02IZP2T	Soluble_methane_monooxygenase_(s	ref|YP_003452126.1|	Bacteria	Proteobacteria	Alphaproteobacteria	Rhodospirillales	Azospirillum sp. B510	100	3.00E-51	methane monooxygenase-like
mmo	LN Root	MID4	GJHFW2E02IJN8N	Soluble_methane_monooxygenase_(s	ref|ZP_03266520.1|	Bacteria	Proteobacteria	Betaproteobacteria	Burkholderiales	Burkholderia sp. H160	93	2.00E-40	Methane monooxygenase
mmo	LN Root	MID4	GJHFW2E02HDGWY	Soluble_methane_monooxygenase_(s	ref|ZP_08631704.1|					Acidiphilium sp. PM	67	1.00E-39	Oxidoreductase FAD/NAD(P)-binding subunit
mmo	SN Root	MID2	GJHFW2E01D5JYF	Soluble_methane_monooxygenase_(s	dbj|BAJ76720.1|	Bacteria	Actinobacteria	Actinobacteria (class)	Actinomycetales	Mycobacterium goodii	75	4.00E-61	phenol and propane monooxygenase small subunit
mmo	SN Root	MID1	GJHFW2E01AP4WJ	Soluble_methane_monooxygenase_(s	emb|CAD88243.1|	Bacteria	Proteobacteria	Alphaproteobacteria	Rhizobiales	Methylocella tundrae	85	1.00E-77	soluble methane monooxygenase component A alpha subunit
mmo	SN Root	MID1	GJHFW2E01D5N24	Soluble_methane_monooxygenase_(s	ref|YP_002361594.1|	Bacteria	Proteobacteria	Alphaproteobacteria	Rhizobiales	Methylocella silvestris BL2	83	7.00E-79	protein A-beta subunit of soluble methane monooxygenase
mmo	SN Root	MID2	GJHFW2E01AFN6Y	Soluble_methane_monooxygenase_(s	ref|YP_002361595.1|	Bacteria	Proteobacteria	Alphaproteobacteria	Rhizobiales	Methylocella silvestris BL2	90	3.00E-32	protein B of soluble methane monooxygenase
mmo	SN Root	MID2	GJHFW2E01C18WT	Soluble_methane_monooxygenase_(s	ref|YP_002361595.1|	Bacteria	Proteobacteria	Alphaproteobacteria	Rhizobiales	Methylocella silvestris BL2	87	1.00E-23	protein B of soluble methane monooxygenase
mmo	SN Root	MID2	GJHFW2E01DKG23	Soluble_methane_monooxygenase_(s	ref|YP_002361595.1|	Bacteria	Proteobacteria	Alphaproteobacteria	Rhizobiales	Methylocella silvestris BL2	87	5.00E-40	protein B of soluble methane monooxygenase
mmo	SN Root	MID2	GJHFW2E01B2M67	Soluble_methane_monooxygenase_(s	ref|YP_002361594.1|	Bacteria	Proteobacteria	Alphaproteobacteria	Rhizobiales	Methylocella silvestris BL2	77	2.00E-71	protein A-beta subunit of soluble methane monooxygenase
mmo	SN Root	MID1	GJHFW2E01C9MRV	Soluble_methane_monooxygenase_(s	ref|YP_001239624.1|	Bacteria	Proteobacteria	Alphaproteobacteria	Rhizobiales	Bradyrhizobium sp. BTAi1	80	2.00E-57	putative methane/phenol/toluene monooxygenase subunit beta
mmo	SN Root	MID1	GJHFW2E01D9IL6	Soluble_methane_monooxygenase_(s	ref|YP_001239624.1|	Bacteria	Proteobacteria	Alphaproteobacteria	Rhizobiales	Bradyrhizobium sp. BTAi1	79	1.00E-35	putative methane/phenol/toluene monooxygenase subunit beta
mmo	SN Root	MID1	GJHFW2E01CM3X9	Soluble_methane_monooxygenase_(s	ref|YP_001239623.1|	Bacteria	Proteobacteria	Alphaproteobacteria	Rhizobiales	Bradyrhizobium sp. BTAi1	76	2.00E-57	putative phenol hydroxylase (Phenol 2-monooxygenase P5 component)
mmo	SN Root	MID2	GJHFW2E01C8JIC	Soluble_methane_monooxygenase_(s	ref|YP_001206321.1|	Bacteria	Proteobacteria	Alphaproteobacteria	Rhizobiales	Bradyrhizobium sp. ORS278	78	7.00E-13	putative methane/phenol/toluene monooxygenase subunit alpha
mmo	SN Root	MID1	GJHFW2E01C6KVW	Soluble_methane_monooxygenase_(s	gb|AAF01268.1|AF153282_1	Bacteria	Proteobacteria	Alphaproteobacteria	Rhizobiales	Methylocystis sp. WI14	95	4.00E-67	MmoX
mmo	SN Root	MID1	GJHFW2E01CQ7Z1	Soluble_methane_monooxygenase_(s	gb|AAF01269.1|AF153282_2	Bacteria	Proteobacteria	Alphaproteobacteria	Rhizobiales	Methylocystis sp. WI14	99	2.00E-64	MmoY
mmo	SN Root	MID2	GJHFW2E01CMRDQ	Soluble_methane_monooxygenase_(s	gb|AAF01268.1|AF153282_1	Bacteria	Proteobacteria	Alphaproteobacteria	Rhizobiales	Methylocystis sp. WI14	98	2.00E-90	MmoX
mmo	SN Root	MID2	GJHFW2E01A5P6M	Soluble_methane_monooxygenase_(s	gb|AAC45290.1|	Bacteria	Proteobacteria	Alphaproteobacteria	Rhizobiales	Methylocystis sp. M	97	9.00E-80	soluble methane monooxygenase protein A beta subunit
mmo	SN Root	MID2	GJHFW2E01DUYWL	Soluble_methane_monooxygenase_(s	gb|AAF01269.1|AF153282_2	Bacteria	Proteobacteria	Alphaproteobacteria	Rhizobiales	Methylocystis sp. WI14	96	9.00E-66	MmoY
mmo	SN Root	MID3	GJHFW2E01B1US7	Soluble_methane_monooxygenase_(s	gb|AAC45289.1|	Bacteria	Proteobacteria	Alphaproteobacteria	Rhizobiales	Methylocystis sp. M	100	5.00E-48	soluble methane monooxygenase protein A alpha subunit
mmo	SN Root	MID3	GJHFW2E01EGZMS	Soluble_methane_monooxygenase_(s	gb|AAF01269.1|AF153282_2	Bacteria	Proteobacteria	Alphaproteobacteria	Rhizobiales	Methylocystis sp. WI14	95	7.00E-77	MmoY
mmo	SN Root	MID1	GJHFW2E01CAFH3	Soluble_methane_monooxygenase_(s	gb|ABD46898.1|	Bacteria	Proteobacteria	Alphaproteobacteria	Rhizobiales	Methylosinus sporium	98	5.00E-51	MmoX2
mmo	SN Root	MID1	GJHFW2E01E1OH0	Soluble_methane_monooxygenase_(s	gb|ABD46892.1|	Bacteria	Proteobacteria	Alphaproteobacteria	Rhizobiales	Methylosinus sporium	98	1.00E-90	MmoX1
mmo	SN Root	MID2	GJHFW2E01B2XFP	Soluble_methane_monooxygenase_(s	gb|ABD46895.1|	Bacteria	Proteobacteria	Alphaproteobacteria	Rhizobiales	Methylosinus sporium	100	2.00E-38	MmoZ
mmo	SN Root	MID2	GJHFW2E01CD1WK	Soluble_methane_monooxygenase_(s	gb|ABD46893.1|	Bacteria	Proteobacteria	Alphaproteobacteria	Rhizobiales	Methylosinus sporium	97	2.00E-45	MmoY
mmo	SN Root	MID2	GJHFW2E01DXAQR	Soluble_methane_monooxygenase_(s	gb|ABD46894.1|	Bacteria	Proteobacteria	Alphaproteobacteria	Rhizobiales	Methylosinus sporium	86	4.00E-60	MmoB
mmo	SN Root	MID2	GJHFW2E01EN8DN	Soluble_methane_monooxygenase_(s	gb|ABD46894.1|	Bacteria	Proteobacteria	Alphaproteobacteria	Rhizobiales	Methylosinus sporium	81	2.00E-29	MmoB
mmo	SN Root	MID2	GJHFW2E01AK62N	Soluble_methane_monooxygenase_(s	gb|ABD46898.1|	Bacteria	Proteobacteria	Alphaproteobacteria	Rhizobiales	Methylosinus sporium	80	4.00E-20	MmoX2
mmo	SN Root	MID2	GJHFW2E01B3WF5	Soluble_methane_monooxygenase_(s	gb|ABD46892.1|	Bacteria	Proteobacteria	Alphaproteobacteria	Rhizobiales	Methylosinus sporium	98	5.00E-49	MmoX1
mmo	SN Root	MID2	GJHFW2E01AXRP0	Soluble_methane_monooxygenase_(s	emb|CAB45257.1|	Bacteria	Proteobacteria	Alphaproteobacteria	Rhizobiales	Methylosinus trichosporium OB3b	78	7.00E-56	Protein C of soluble methane monooxygenase (sMMO)
mmo	SN Root	MID2	GJHFW2E01DZ0JU	Soluble_methane_monooxygenase_(s	gb|ABD46897.1|	Bacteria	Proteobacteria	Alphaproteobacteria	Rhizobiales	Methylosinus sporium	99	1.00E-74	MmoC
mmo	SN Root	MID2	GJHFW2E01EBZLN	Soluble_methane_monooxygenase_(s	gb|AAZ81973.1|	Bacteria	Proteobacteria	Alphaproteobacteria	Rhizobiales	Methylosinus trichosporium	94	2.00E-66	protein C of soluble methane monooxygenase

mcr	LN Root	MID4	GJHFW2E02IRX3T	Methanogenesis	ref|YP_004291464.1|	Archaea	Euryarchaeota	Methanobacteria	Methanobacteriales	Methanobacterium sp. AL-21	80	6.00E-06	methyl-coenzyme M reductase subunit beta
mcr	LN Root	MID4	GJHFW2E02JRX6S	Methanogenesis	ref|YP_004520871.1|	Archaea	Euryarchaeota	Methanobacteria	Methanobacteriales	Methanobacterium sp. SWAN-1	78	6.00E-47	methanogenesis marker protein 10
mcr	LN Root	MID5	GJHFW2E02HBAVR	Methanogenesis	ref|YP_004291467.1|	Archaea	Euryarchaeota	Methanobacteria	Methanobacteriales	Methanobacterium sp. AL-21	88	2.00E-68	methyl-coenzyme M reductase subunit alpha
mcr	LN Root	MID5	GJHFW2E02HVYHC	Methanogenesis	ref|YP_004520868.1|	Archaea	Euryarchaeota	Methanobacteria	Methanobacteriales	Methanobacterium sp. SWAN-1	83	1.00E-62	methyl-coenzyme M reductase I operon protein C
mcr	LN Root	MID4	GJHFW2E02IT0QA	Methanogenesis	ref|NP_276296.1|	Archaea	Euryarchaeota	Methanobacteria	Methanobacteriales	Methanothermobacter thermautotrophicus str. Delta H	79	3.00E-51	methyl coenzyme M reductase I, beta subunit
mcr	LN Root	MID4	GJHFW2E02JXNE3	Methanogenesis	ref|NP_276296.1|	Archaea	Euryarchaeota	Methanobacteria	Methanobacteriales	Methanothermobacter thermautotrophicus str. Delta H	75	1.00E-33	methyl coenzyme M reductase I, beta subunit
mcr	LN Root	MID6	GJHFW2E02I9MWC	Methanogenesis	gb|AAA73443.1|	Archaea	Euryarchaeota	Methanobacteria	Methanobacteriales	Methanothermobacter thermautotrophicus	80	2.00E-52	methyl coenzyme M reductase I C protein
mcr	LN Root	MID6	GJHFW2E02F7QFR	Methanogenesis	gb|AAA73443.1|	Archaea	Euryarchaeota	Methanobacteria	Methanobacteriales	Methanothermobacter thermautotrophicus	83	5.00E-32	methyl coenzyme M reductase I C protein
mcr	LN Root	MID4	GJHFW2E02HI737	Methanogenesis	ref|YP_003458728.1|	Archaea	Euryarchaeota	Methanococci	Methanococcales	Methanocaldococcus sp. FS406-22	84	3.00E-27	methyl-coenzyme M reductase, alpha subunit
mcr	LN Root	MID4	GJHFW2E02IZ35Z	Methanogenesis	ref|YP_003457854.1|	Archaea	Euryarchaeota	Methanococci	Methanococcales	Methanocaldococcus sp. FS406-22	92	3.00E-66	methyl-coenzyme M reductase, alpha subunit
mcr	LN Root	MID4	GJHFW2E02HJYCP	Methanogenesis	ref|NP_619407.1|	Archaea	Euryarchaeota	Methanomicrobia	Methanosarcinales	Methanosarcina acetivorans C2A	80	3.00E-53	methyl coenzyme M reductase, subunit D
mcr	LN Root	MID5	GJHFW2E02HMP9F	Methanogenesis	ref|NP_619407.1|	Archaea	Euryarchaeota	Methanomicrobia	Methanosarcinales	Methanosarcina acetivorans C2A	90	5.00E-36	methyl coenzyme M reductase, subunit D
mcr	LN Root	MID4	GJHFW2E02F7RWL	Methanogenesis	ref|YP_503931.1|	Archaea	Euryarchaeota	Methanomicrobia	Methanomicrobiales	Methanospirillum hungatei JF-1	69	2.00E-26	hypothetical protein Mhun_2512
mcr	SN Root	MID1	GJHFW2E01D5IRA	Methanogenesis	ref|YP_004520871.1|	Archaea	Euryarchaeota	Methanobacteria	Methanobacteriales	Methanobacterium sp. SWAN-1	75	8.00E-20	methanogenesis marker protein 10
mcr	SN Root	MID1	GJHFW2E01EQTOX	Methanogenesis	ref|YP_004291467.1|	Archaea	Euryarchaeota	Methanobacteria	Methanobacteriales	Methanobacterium sp. AL-21	87	3.00E-65	methyl-coenzyme M reductase subunit alpha
mcr	SN Root	MID1	GJHFW2E01EXZDR	Methanogenesis	ref|YP_004291467.1|	Archaea	Euryarchaeota	Methanobacteria	Methanobacteriales	Methanobacterium sp. AL-21	70	5.00E-40	methyl-coenzyme M reductase subunit alpha
mcr	SN Root	MID1	GJHFW2E01BGMZP	Methanogenesis	ref|YP_004291464.1|	Archaea	Euryarchaeota	Methanobacteria	Methanobacteriales	Methanobacterium sp. AL-21	85	1.00E-64	methyl-coenzyme M reductase subunit beta
mcr	SN Root	MID1	GJHFW2E01AFA1V	Methanogenesis	ref|YP_004291464.1|	Archaea	Euryarchaeota	Methanobacteria	Methanobacteriales	Methanobacterium sp. AL-21	85	4.00E-46	methyl-coenzyme M reductase subunit beta
mcr	SN Root	MID1	GJHFW2E01DS3TL	Methanogenesis	ref|YP_004289573.1|	Archaea	Euryarchaeota	Methanobacteria	Methanobacteriales	Methanobacterium sp. AL-21	72	2.00E-53	methyl-coenzyme M reductase subunit beta
mcr	SN Root	MID1	GJHFW2E01AVZG6	Methanogenesis	ref|YP_004291465.1|	Archaea	Euryarchaeota	Methanobacteria	Methanobacteriales	Methanobacterium sp. AL-21	79	1.00E-29	methyl-coenzyme M reductase operon protein D
mcr	SN Root	MID1	GJHFW2E01DWUBQ	Methanogenesis	ref|YP_004291464.1|	Archaea	Euryarchaeota	Methanobacteria	Methanobacteriales	Methanobacterium sp. AL-21	83	2.00E-43	methyl-coenzyme M reductase subunit beta
mcr	SN Root	MID1	GJHFW2E01BB2OK	Methanogenesis	ref|YP_004289577.1|	Archaea	Euryarchaeota	Methanobacteria	Methanobacteriales	Methanobacterium sp. AL-21	100	1.00E-81	methyl-coenzyme M reductase subunit alpha
mcr	SN Root	MID1	GJHFW2E01DBE9E	Methanogenesis	ref|YP_004520866.1|	Archaea	Euryarchaeota	Methanobacteria	Methanobacteriales	Methanobacterium sp. SWAN-1	81	1.00E-62	methyl-coenzyme M reductase subunit alpha
mcr	SN Root	MID1	GJHFW2E01DVR1K	Methanogenesis	ref|YP_004520866.1|	Archaea	Euryarchaeota	Methanobacteria	Methanobacteriales	Methanobacterium sp. SWAN-1	91	3.00E-65	methyl-coenzyme M reductase subunit alpha
mcr	SN Root	MID1	GJHFW2E01D9KLH	Methanogenesis	ref|YP_004291466.1|	Archaea	Euryarchaeota	Methanobacteria	Methanobacteriales	Methanobacterium sp. AL-21	83	2.00E-53	methyl-coenzyme M reductase subunit gamma
mcr	SN Root	MID1	GJHFW2E01E4DPE	Methanogenesis	ref|YP_004291466.1|	Archaea	Euryarchaeota	Methanobacteria	Methanobacteriales	Methanobacterium sp. AL-21	81	3.00E-59	methyl-coenzyme M reductase subunit gamma
mcr	SN Root	MID1	GJHFW2E01BXZ1R	Methanogenesis	ref|YP_004520868.1|	Archaea	Euryarchaeota	Methanobacteria	Methanobacteriales	Methanobacterium sp. SWAN-1	69	2.00E-21	methyl-coenzyme M reductase I operon protein C
mcr	SN Root	MID1	GJHFW2E01A8GC7	Methanogenesis	ref|YP_004520869.1|	Archaea	Euryarchaeota	Methanobacteria	Methanobacteriales	Methanobacterium sp. SWAN-1	65	7.00E-24	methyl-coenzyme M reductase operon protein D
mcr	SN Root	MID2	GJHFW2E01BTGJW	Methanogenesis	ref|YP_004291467.1|	Archaea	Euryarchaeota	Methanobacteria	Methanobacteriales	Methanobacterium sp. AL-21	82	1.00E-65	methyl-coenzyme M reductase subunit alpha
mcr	SN Root	MID2	GJHFW2E01DXPTX	Methanogenesis	ref|YP_004291466.1|	Archaea	Euryarchaeota	Methanobacteria	Methanobacteriales	Methanobacterium sp. AL-21	82	5.00E-51	methyl-coenzyme M reductase subunit gamma
mcr	SN Root	MID2	GJHFW2E01ESJFY	Methanogenesis	dbj|BAI67101.1|	Archaea	Euryarchaeota	Methanobacteria	Methanobacteriales	Methanobacterium oryzae	100	6.00E-30	methyl-coenzyme M reductase alpha subunit
mcr	SN Root	MID2	GJHFW2E01B1UK3	Methanogenesis	ref|YP_004291466.1|	Archaea	Euryarchaeota	Methanobacteria	Methanobacteriales	Methanobacterium sp. AL-21	95	6.00E-62	methyl-coenzyme M reductase subunit gamma
mcr	SN Root	MID2	GJHFW2E01EX2YB	Methanogenesis	ref|YP_004291464.1|	Archaea	Euryarchaeota	Methanobacteria	Methanobacteriales	Methanobacterium sp. AL-21	78	9.00E-57	methyl-coenzyme M reductase subunit beta
mcr	SN Root	MID2	GJHFW2E01D2ENF	Methanogenesis	ref|YP_004520871.1|	Archaea	Euryarchaeota	Methanobacteria	Methanobacteriales	Methanobacterium sp. SWAN-1	68	2.00E-56	methanogenesis marker protein 10
mcr	SN Root	MID2	GJHFW2E01EE3NJ	Methanogenesis	ref|YP_004520871.1|	Archaea	Euryarchaeota	Methanobacteria	Methanobacteriales	Methanobacterium sp. SWAN-1	67	1.00E-36	methanogenesis marker protein 11
mcr	SN Root	MID2	GJHFW2E01DI6CO	Methanogenesis	ref|YP_004289573.1|	Archaea	Euryarchaeota	Methanobacteria	Methanobacteriales	Methanobacterium sp. AL-21	90	2.00E-66	methyl-coenzyme M reductase subunit beta
mcr	SN Root	MID2	GJHFW2E01C55B6	Methanogenesis	ref|YP_004289573.1|	Archaea	Euryarchaeota	Methanobacteria	Methanobacteriales	Methanobacterium sp. AL-21	100	5.00E-10	methyl-coenzyme M reductase subunit beta
mcr	SN Root	MID2	GJHFW2E01E1FZ3	Methanogenesis	ref|YP_004291464.1|	Archaea	Euryarchaeota	Methanobacteria	Methanobacteriales	Methanobacterium sp. AL-21	87	1.00E-54	methyl-coenzyme M reductase subunit beta
mcr	SN Root	MID3	GJHFW2E01BZ6ET	Methanogenesis	ref|YP_004291465.1|	Archaea	Euryarchaeota	Methanobacteria	Methanobacteriales	Methanobacterium sp. AL-21	67	1.00E-30	methyl-coenzyme M reductase operon protein D
mcr	SN Root	MID2	GJHFW2E01C01YO	Methanogenesis	ref|ZP_05975544.1|	Archaea	Euryarchaeota	Methanobacteria	Methanobacteriales	Methanobrevibacter smithii DSM 2374	80	4.00E-58	methyl-coenzyme M reductase, alpha subunit
mcr	SN Root	MID1	GJHFW2E01DWOCE	Methanogenesis	ref|YP_447374.1|	Archaea	Euryarchaeota	Methanobacteria	Methanobacteriales	Methanosphaera stadtmanae DSM 3091	77	1.00E-38	MrtA
mcr	SN Root	MID1	GJHFW2E01B1QJ0	Methanogenesis	ref|YP_003850442.1|	Archaea	Euryarchaeota	Methanobacteria	Methanobacteriales	Methanothermobacter marburgensis str. Marburg	86	4.00E-34	methyl-coenzyme M reductase I, subunit epsilon
mcr	SN Root	MID1	GJHFW2E01DW724	Methanogenesis	gb|AAA73445.1|	Archaea	Euryarchaeota	Methanobacteria	Methanobacteriales	Methanothermobacter thermautotrophicus	87	4.00E-52	methyl coenzyme M reductase I alpha subunit
mcr	SN Root	MID1	GJHFW2E01EBHUA	Methanogenesis	pdb|3POT|A	Archaea	Euryarchaeota	Methanobacteria	Methanobacteriales		84	5.00E-29	Methyl-Coenzyme M Reductase Chain D,
mcr	SN Root	MID1	GJHFW2E01EVS66	Methanogenesis	ref|NP_276296.1|	Archaea	Euryarchaeota	Methanobacteria	Methanobacteriales	Methanothermobacter thermautotrophicus str. Delta H	87	7.00E-45	methyl coenzyme M reductase I, beta subunit
mcr	SN Root	MID1	GJHFW2E01CR2X1	Methanogenesis	ref|NP_276296.1|	Archaea	Euryarchaeota	Methanobacteria	Methanobacteriales	Methanothermobacter thermautotrophicus str. Delta H	75	7.00E-61	methyl coenzyme M reductase I, beta subunit
mcr	SN Root	MID2	GJHFW2E01AFL3R	Methanogenesis	ref|NP_276296.1|	Archaea	Euryarchaeota	Methanobacteria	Methanobacteriales	Methanothermobacter thermautotrophicus str. Delta H	85	9.00E-63	methyl coenzyme M reductase I, beta subunit
mcr	SN Root	MID2	GJHFW2E01D90EJ	Methanogenesis	ref|NP_276296.1|	Archaea	Euryarchaeota	Methanobacteria	Methanobacteriales	Methanothermobacter thermautotrophicus str. Delta H	89	8.00E-27	methyl coenzyme M reductase I, beta subunit
mcr	SN Root	MID2	GJHFW2E01DCOID	Methanogenesis	ref|NP_276296.1|	Archaea	Euryarchaeota	Methanobacteria	Methanobacteriales	Methanothermobacter thermautotrophicus str. Delta H	86	4.00E-59	methyl coenzyme M reductase I, beta subunit
mcr	SN Root	MID2	GJHFW2E01EPQLK	Methanogenesis	ref|NP_276296.1|	Archaea	Euryarchaeota	Methanobacteria	Methanobacteriales	Methanothermobacter thermautotrophicus str. Delta H	73	3.00E-25	methyl coenzyme M reductase I, beta subunit
mcr	SN Root	MID2	GJHFW2E01EZOLZ	Methanogenesis	gb|AAA73445.1|	Archaea	Euryarchaeota	Methanobacteria	Methanobacteriales	Methanothermobacter thermautotrophicus	87	1.00E-46	methyl coenzyme M reductase I alpha subunit
mcr	SN Root	MID3	GJHFW2E01CLZFN	Methanogenesis	gb|ACJ03594.1|	Archaea	Euryarchaeota	Methanobacteria	Methanobacteriales	Methanothermobacter marburgensis	68	1.00E-54	methyl-coenzyme M reductase I gamma subunit
mcr	SN Root	MID2	GJHFW2E01EM7J6	Methanogenesis	ref|YP_003355567.1|	Archaea	Euryarchaeota	Methanomicrobia	Methanocellales	Methanocella paludicola SANAE	97	1.00E-28	methyl-coenzyme M reductase beta subunit
mcr	SN Root	MID1	GJHFW2E01CE9LA	Methanogenesis	ref|NP_619409.1|	Archaea	Euryarchaeota	Methanomicrobia	Methanosarcinales	Methanosarcina acetivorans C2A	73	1.00E-10	methyl coenzyme M reductase, subunit beta
mcr	SN Root	MID1	GJHFW2E01DMMYH	Methanogenesis	ref|NP_633268.1|	Archaea	Euryarchaeota	Methanomicrobia	Methanosarcinales	Methanosarcina mazei Go1	86	9.00E-34	methyl-coenzyme M reductase subunit beta
mcr	SN Root	MID1	GJHFW2E01EXAZ0	Methanogenesis	ref|NP_633269.1|	Archaea	Euryarchaeota	Methanomicrobia	Methanosarcinales	Methanosarcina mazei Go1	87	5.00E-30	hypothetical protein MM_1245
mcr	SN Root	MID2	GJHFW2E01DW94N	Methanogenesis	ref|NP_633268.1|	Archaea	Euryarchaeota	Methanomicrobia	Methanosarcinales	Methanosarcina mazei Go1	88	2.00E-04	methyl-coenzyme M reductase subunit beta
mcr	SN Root	MID2	GJHFW2E01BP627	Methanogenesis	ref|NP_619410.1|	Archaea	Euryarchaeota	Methanomicrobia	Methanosarcinales	Methanosarcina acetivorans C2A	82	3.00E-70	hypothetical protein MA4551
mcr	SN Root	MID2	GJHFW2E01B9OSV	Methanogenesis	ref|NP_619408.1|	Archaea	Euryarchaeota	Methanomicrobia	Methanosarcinales	Methanosarcina acetivorans C2A	82	7.00E-21	methyl coenzyme M reductase, subunit C
mcr	SN Root	MID2	GJHFW2E01E5GDR	Methanogenesis	pdb|1E6Y|A	Archaea	Euryarchaeota	Methanomicrobia	Methanosarcinales		93	6.00E-78	Chain A, Methyl-Coenzyme M Reductase
mcr	SN Root	MID2	GJHFW2E01BCX8S	Methanogenesis	ref|YP_304447.1|	Archaea	Euryarchaeota	Methanomicrobia	Methanosarcinales	Methanosarcina barkeri str. Fusaro	81	7.00E-47	methyl-coenzyme M reductase subunit alpha
mcr	SN Root	MID2	GJHFW2E01EJNCF	Methanogenesis	ref|YP_304452.1|	Archaea	Euryarchaeota	Methanomicrobia	Methanosarcinales	Methanosarcina barkeri str. Fusaro	84	7.00E-61	hypothetical protein Mbar_A0898
mcr	SN Root	MID1	GJHFW2E01BI22S	Methanogenesis	ref|YP_686526.1|	Archaea	Euryarchaeota			uncultured methanogenic archaeon RC-I	79	8.00E-44	methyl-coenzyme M reductase, beta subunit
mcr	SN Root	MID2	GJHFW2E01CHRZZ	Methanogenesis	ref|YP_004384715.1|					Methanosaeta concilii GP6	79	4.00E-46	putative methanogenesis marker protein 10
mcr	SN Root	MID3	GJHFW2E01BIRP1	Methanogenesis	ref|YP_686528.1|	Archaea	Euryarchaeota			uncultured methanogenic archaeon RC-I	93	2.00E-60	methyl-coenzyme M reductase, operon protein C

acdS	LN Root	MID6	GJHFW2E02IBPUH	Experimental-COG2515	ref|YP_001104984.1|	Bacteria	Actinobacteria	Actinobacteria (class)	Actinomycetales	Saccharopolyspora erythraea NRRL 2338	88	2.00E-24	1-aminocyclopropane-1-carboxylate deaminase
acdS	LN Root	MID4	GJHFW2E02GN5BR	Experimental-COG2515	ref|YP_001205796.1|	Bacteria	Proteobacteria	Alphaproteobacteria	Rhizobiales	Bradyrhizobium sp. ORS278	96	8.00E-22	1-aminocyclopropane-1-carboxylate deaminase
acdS	LN Root	MID4	GJHFW2E02G2HZQ	Experimental-COG2515	ref|YP_001240096.1|	Bacteria	Proteobacteria	Alphaproteobacteria	Rhizobiales	Bradyrhizobium sp. BTAi1	96	3.00E-28	1-aminocyclopropane-1-carboxylate deaminase
acdS	LN Root	MID4	GJHFW2E02II265	Experimental-COG2515	ref|YP_001240096.1|	Bacteria	Proteobacteria	Alphaproteobacteria	Rhizobiales	Bradyrhizobium sp. BTAi1	98	9.00E-55	1-aminocyclopropane-1-carboxylate deaminase
acdS	LN Root	MID5	GJHFW2E02FHO9H	Experimental-COG2515	ref|YP_001240096.1|	Bacteria	Proteobacteria	Alphaproteobacteria	Rhizobiales	Bradyrhizobium sp. BTAi1	95	3.00E-36	1-aminocyclopropane-1-carboxylate deaminase
acdS	LN Root	MID6	GJHFW2E02HMJLV	Experimental-COG2515	ref|YP_001205796.1|	Bacteria	Proteobacteria	Alphaproteobacteria	Rhizobiales	Bradyrhizobium sp. ORS278	95	7.00E-61	1-aminocyclopropane-1-carboxylate deaminase
acdS	LN Root	MID6	GJHFW2E02GNVQ2	Experimental-COG2515	ref|NP_766881.1|	Bacteria	Proteobacteria	Alphaproteobacteria	Rhizobiales	Bradyrhizobium japonicum USDA 110	97	1.00E-30	1-aminocyclopropane-1-carboxylate deaminase
acdS	LN Root	MID4	GJHFW2E02F71W8	Experimental-COG2515	gb|ABE66282.2|	Bacteria	Proteobacteria	Alphaproteobacteria	Rhodospirillales	Azospirillum lipoferum	94	3.00E-76	1-aminocyclopropane-1-carboxylate deaminase
acdS	LN Root	MID4	GJHFW2E02FGZ4D	Experimental-COG2515	ref|YP_003451624.1|	Bacteria	Proteobacteria	Alphaproteobacteria	Rhodospirillales	Azospirillum sp. B510	86	2.00E-16	1-aminocyclopropane-1-carboxylate deaminase
acdS	LN Root	MID4	GJHFW2E02H7114	Experimental-COG2515	gb|ABE66282.2|	Bacteria	Proteobacteria	Alphaproteobacteria	Rhodospirillales	Azospirillum lipoferum	86	8.00E-57	1-aminocyclopropane-1-carboxylate deaminase
acdS	LN Root	MID4	GJHFW2E02IOQID	Experimental-COG2515	gb|ABE66293.1|	Bacteria	Proteobacteria	Alphaproteobacteria	Rhodospirillales	Azospirillum lipoferum	94	4.00E-55	1-aminocyclopropane-1-carboxylate deaminase
acdS	LN Root	MID4	GJHFW2E02JDY4R	Experimental-COG2515	gb|ABE66282.2|	Bacteria	Proteobacteria	Alphaproteobacteria	Rhodospirillales	Azospirillum lipoferum	92	2.00E-54	1-aminocyclopropane-1-carboxylate deaminase
acdS	LN Root	MID4	GJHFW2E02JNBGS	Experimental-COG2515	gb|ABE66282.2|	Bacteria	Proteobacteria	Alphaproteobacteria	Rhodospirillales	Azospirillum lipoferum	94	6.00E-49	1-aminocyclopropane-1-carboxylate deaminase
acdS	LN Root	MID4	GJHFW2E02JDZY1	Experimental-COG2515	ref|YP_003451624.1|	Bacteria	Proteobacteria	Alphaproteobacteria	Rhodospirillales	Azospirillum sp. B510	70	2.00E-23	1-aminocyclopropane-1-carboxylate deaminase
acdS	LN Root	MID5	GJHFW2E02FTGTI	Experimental-COG2515	gb|ACS92701.1|	Bacteria	Proteobacteria	Alphaproteobacteria	Rhodospirillales	Azospirillum lipoferum	76	2.00E-19	Lrp-like regulator of acdS
acdS	LN Root	MID5	GJHFW2E02GSBOQ	Experimental-COG2515	ref|YP_003451624.1|	Bacteria	Proteobacteria	Alphaproteobacteria	Rhodospirillales	Azospirillum sp. B510	94	3.00E-44	1-aminocyclopropane-1-carboxylate deaminase
acdS	LN Root	MID4	GJHFW2E02FKB32	Experimental-COG2515	ref|ZP_06838775.1|	Bacteria	Proteobacteria	Betaproteobacteria	Burkholderiales	Burkholderia sp. Ch1-1	92	6.00E-50	1-aminocyclopropane-1-carboxylate deaminase
acdS	LN Root	MID4	GJHFW2E02JK8XV	Experimental-COG2515	gb|ACH81539.1|	Bacteria	Proteobacteria	Betaproteobacteria	Burkholderiales	Burkholderia kururiensis	100	1.00E-63	1-aminocyclopropane-1-carboxylate deaminase
acdS	LN Root	MID4	GJHFW2E02F5NJ7	Experimental-COG2515	gb|ACH81539.1|	Bacteria	Proteobacteria	Betaproteobacteria	Burkholderiales	Burkholderia kururiensis	100	1.00E-70	1-aminocyclopropane-1-carboxylate deaminase
acdS	LN Root	MID4	GJHFW2E02HGNRW	Experimental-COG2515	gb|ACH81532.1|	Bacteria	Proteobacteria	Betaproteobacteria	Burkholderiales	Burkholderia vietnamiensis	99	4.00E-84	1-aminocyclopropane-1-carboxylate deaminase
acdS	LN Root	MID4	GJHFW2E02HBIFO	Experimental-COG2515	ref|YP_001892430.1|	Bacteria	Proteobacteria	Betaproteobacteria	Burkholderiales	Ralstonia pickettii 12D	75	2.00E-21	protein of unknown function DUF1697
acdS	LN Root	MID6	GJHFW2E02HNPMO	Experimental-COG2515	ref|YP_001892430.1|	Bacteria	Proteobacteria	Betaproteobacteria	Burkholderiales	Ralstonia pickettii 12D	77	2.00E-22	protein of unknown function DUF1697
acdS	SN Root	MID2	GJHFW2E01DBX8L	Experimental-COG2515	ref|YP_003380387.1|	Bacteria	Actinobacteria	Actinobacteria (class)	Actinomycetales	Kribbella flavida DSM 17836	87	7.00E-61	1-aminocyclopropane-1-carboxylate deaminase
acdS	SN Root	MID1	GJHFW2E01DNBIY	Experimental-COG2515	ref|ZP_08199237.1|	Bacteria	Actinobacteria	Actinobacteria (class)	Actinomycetales	Nocardioidaceae bacterium Broad-1	82	2.00E-49	1-aminocyclopropane-1-carboxylate deaminase
acdS	SN Root	MID2	GJHFW2E01BKMGL	Experimental-COG2515	ref|ZP_06916233.1|	Bacteria	Actinobacteria	Actinobacteria (class)	Actinomycetales	Streptomyces sviceus ATCC 29083	67	1.00E-43	1-aminocyclopropane-1-carboxylate deaminase
acdS	SN Root	MID2	GJHFW2E01AKE8L	Experimental-COG2515	ref|YP_003451624.1|	Bacteria	Proteobacteria	Alphaproteobacteria	Rhodospirillales	Azospirillum sp. B510	95	4.00E-42	1-aminocyclopropane-1-carboxylate deaminase
acdS	SN Root	MID2	GJHFW2E01AKEFS	Experimental-COG2515	ref|YP_003451624.1|	Bacteria	Proteobacteria	Alphaproteobacteria	Rhodospirillales	Azospirillum sp. B510	93	4.00E-77	1-aminocyclopropane-1-carboxylate deaminase
acdS	SN Root	MID1	GJHFW2E01A0ZHH	Experimental-COG2515	gb|ACH81532.1|	Bacteria	Proteobacteria	Betaproteobacteria	Burkholderiales	Burkholderia vietnamiensis	100	7.00E-29	1-aminocyclopropane-1-carboxylate deaminase
acdS	SN Root	MID1	GJHFW2E01B5CAL	Experimental-COG2515	ref|YP_008120.1|	Bacteria	Chlamydiae	Chlamydiae (class)	Chlamydiales	Candidatus Protochlamydia amoebophila UWE25	46	7.00E-29	putative 1-aminocyclopropane-1-carboxylate deaminase (ACC deaminase)
acdS	SN Root	MID1	GJHFW2E01BOLT7	Experimental-COG2515	ref|YP_008120.1|	Bacteria	Chlamydiae	Chlamydiae (class)	Chlamydiales	Candidatus Protochlamydia amoebophila UWE25	46	4.00E-31	putative 1-aminocyclopropane-1-carboxylate deaminase (ACC deaminase)

iaaH	LN Root	MID4	GJHFW2E02FM58J	Aromatic_amino_acid_degradation	ref|YP_001207672.1|	Bacteria	Proteobacteria	Alphaproteobacteria	Rhizobiales	Bradyrhizobium sp. ORS278	82	9.00E-47	amidase emb|CAL79455.1| putative amidase
iaaH	LN Root	MID4	GJHFW2E02FO6YP	Aromatic_amino_acid_degradation	ref|YP_001242120.1|	Bacteria	Proteobacteria	Alphaproteobacteria	Rhizobiales	Bradyrhizobium sp. BTAi1	64	1.00E-36	amidase gb|ABQ38214.1| putative amidase
iaaH	LN Root	MID4	GJHFW2E02GEH2Z	Aromatic_amino_acid_degradation	ref|YP_001204182.1|	Bacteria	Proteobacteria	Alphaproteobacteria	Rhizobiales	Bradyrhizobium sp. ORS278	38	2.00E-21	Indoleacetamide hydrolase (IAH) (Indole-3-acetamide hydrolase)
iaaH	LN Root	MID4	GJHFW2E02HIN6I	Aromatic_amino_acid_degradation	ref|YP_001207500.1|	Bacteria	Proteobacteria	Alphaproteobacteria	Rhizobiales	Bradyrhizobium sp. ORS278	80	3.00E-49	putative amidase emb|CAL79283.1| putative amidase
iaaH	LN Root	MID6	GJHFW2E02IDTU3	Aromatic_amino_acid_degradation	ref|YP_001238475.1|	Bacteria	Proteobacteria	Alphaproteobacteria	Rhizobiales	Bradyrhizobium sp. BTAi1	40	4.00E-20	indoleacetamide hydrolase
iaaH	LN Root	MID6	GJHFW2E02IFN8B	Aromatic_amino_acid_degradation	ref|YP_001242120.1|	Bacteria	Proteobacteria	Alphaproteobacteria	Rhizobiales	Bradyrhizobium sp. BTAi1	69	4.00E-45	amidase gb|ABQ38214.1| putative amidase
iaaH	LN Root	MID4	GJHFW2E02F3N3Y	Aromatic_amino_acid_degradation	ref|YP_782613.1|	Bacteria	Proteobacteria	Alphaproteobacteria	Rhizobiales	Rhodopseudomonas palustris BisA53	83	1.00E-26	amidase gb|ABJ07633.1| Amidase
iaaH	LN Root	MID4	GJHFW2E02HHXHF	Aromatic_amino_acid_degradation	ref|YP_001993120.1|	Bacteria	Proteobacteria	Alphaproteobacteria	Rhizobiales	Rhodopseudomonas palustris TIE-1	73	7.00E-34	amidase gb|ACF02645.1| Amidase
iaaH	LN Root	MID4	GJHFW2E02IVV95	Aromatic_amino_acid_degradation	ref|YP_485515.1|	Bacteria	Proteobacteria	Alphaproteobacteria	Rhizobiales	Rhodopseudomonas palustris HaA2	73	7.00E-39	amidase gb|ABD06604.1| Amidase
iaaH	LN Root	MID4	GJHFW2E02JWNPV	Aromatic_amino_acid_degradation	ref|YP_001993120.1|	Bacteria	Proteobacteria	Alphaproteobacteria	Rhizobiales	Rhodopseudomonas palustris TIE-1	72	9.00E-58	amidase gb|ACF02645.1| Amidase
iaaH	LN Root	MID6	GJHFW2E02F1OX1	Aromatic_amino_acid_degradation	ref|YP_782613.1|	Bacteria	Proteobacteria	Alphaproteobacteria	Rhizobiales	Rhodopseudomonas palustris BisA53	73	4.00E-38	amidase gb|ABJ07633.1| Amidase
iaaH	LN Root	MID5	GJHFW2E02JD3LK	Aromatic_amino_acid_degradation	ref|YP_001753587.1|	Bacteria	Proteobacteria	Alphaproteobacteria	Rhizobiales	Methylobacterium radiotolerans JCM 2831	70	8.00E-25	amidase gb|ACB22904.1| Amidase
iaaH	LN Root	MID4	GJHFW2E02FF2I9	Aromatic_amino_acid_degradation	ref|ZP_05122334.1|	Bacteria	Proteobacteria	Alphaproteobacteria	Rhodobacterales	Rhodobacteraceae bacterium KLH11	71	5.00E-15	indoleacetamide hydrolase gb|EEE36966.1| indoleacetamide hydrolase
iaaH	LN Root	MID4	GJHFW2E02JM7EQ	Aromatic_amino_acid_degradation	ref|YP_001859673.1|	Bacteria	Proteobacteria	Betaproteobacteria	Burkholderiales	Burkholderia phymatum STM815	72	3.00E-60	amidase gb|ACC72627.1| Amidase
iaaH	LN Root	MID4	GJHFW2E02JX728	Aromatic_amino_acid_degradation	ref|YP_001859673.1|	Bacteria	Proteobacteria	Betaproteobacteria	Burkholderiales	Burkholderia phymatum STM815	57	5.00E-25	amidase gb|ACC72627.1| Amidase
iaaH	LN Root	MID4	GJHFW2E02HG985	Aromatic_amino_acid_degradation	ref|YP_001116850.1|	Bacteria	Proteobacteria	Betaproteobacteria	Burkholderiales	Burkholderia vietnamiensis G4	82	2.00E-43	indole acetimide hydrolase gb|ABO57385.1| Amidase
iaaH	LN Root	MID4	GJHFW2E02JWGHK	Aromatic_amino_acid_degradation	ref|YP_001116850.1|	Bacteria	Proteobacteria	Betaproteobacteria	Burkholderiales	Burkholderia vietnamiensis G4	96	1.00E-44	indole acetimide hydrolase gb|ABO57385.1| Amidase
iaaH	LN Root	MID4	GJHFW2E02FL3RH	Aromatic_amino_acid_degradation	ref|YP_001889425.1|	Bacteria	Proteobacteria	Betaproteobacteria	Burkholderiales	Burkholderia phytofirmans PsJN	60	7.00E-13	indole acetimide hydrolase gb|ACD20055.1| Amidase
iaaH	LN Root	MID4	GJHFW2E02GEGO1	Aromatic_amino_acid_degradation	ref|YP_001859673.1|	Bacteria	Proteobacteria	Betaproteobacteria	Burkholderiales	Burkholderia phymatum STM815	64	3.00E-34	amidase gb|ACC72627.1| Amidase
iaaH	LN Root	MID4	GJHFW2E02GK1SJ	Aromatic_amino_acid_degradation	ref|YP_001859673.1|	Bacteria	Proteobacteria	Betaproteobacteria	Burkholderiales	Burkholderia phymatum STM815	69	3.00E-52	amidase gb|ACC72627.1| Amidase
iaaH	LN Root	MID4	GJHFW2E02GZJL1	Aromatic_amino_acid_degradation	ref|ZP_03264071.1|	Bacteria	Proteobacteria	Betaproteobacteria	Burkholderiales	Burkholderia sp. H160	60	3.00E-45	Amidase gb|EEA04445.1| Amidase
iaaH	LN Root	MID4	GJHFW2E02IS3RY	Aromatic_amino_acid_degradation	ref|YP_001859673.1|	Bacteria	Proteobacteria	Betaproteobacteria	Burkholderiales	Burkholderia phymatum STM815	66	1.00E-45	amidase gb|ACC72627.1| Amidase
iaaH	LN Root	MID4	GJHFW2E02F8QJ1	Aromatic_amino_acid_degradation	ref|YP_366363.1|	Bacteria	Proteobacteria	Betaproteobacteria	Burkholderiales	Burkholderia sp. 383	64	2.00E-36	amidase gb|ABB05719.1| Amidase
iaaH	LN Root	MID6	GJHFW2E02GF23C	Aromatic_amino_acid_degradation	ref|YP_001859673.1|	Bacteria	Proteobacteria	Betaproteobacteria	Burkholderiales	Burkholderia phymatum STM815	75	3.00E-60	amidase gb|ACC72627.1| Amidase
iaaH	LN Root	MID6	GJHFW2E02I1CV2	Aromatic_amino_acid_degradation	ref|YP_001859673.1|	Bacteria	Proteobacteria	Betaproteobacteria	Burkholderiales	Burkholderia phymatum STM815	67	5.00E-46	amidase gb|ACC72627.1| Amidase
iaaH	LN Root	MID4	GJHFW2E02HHIWQ	Aromatic_amino_acid_degradation	ref|YP_585697.1|	Bacteria	Proteobacteria	Betaproteobacteria	Burkholderiales	Cupriavidus metallidurans CH34	58	4.00E-36	indoleacetamide hydrolase (IAH) (Indole-3-acetamide hydrolase)
iaaH	LN Root	MID4	GJHFW2E02F6X0G	Aromatic_amino_acid_degradation	ref|YP_585697.1|	Bacteria	Proteobacteria	Betaproteobacteria	Burkholderiales	Cupriavidus metallidurans CH34	56	2.00E-26	indoleacetamide hydrolase (IAH) (Indole-3-acetamide hydrolase)
iaaH	LN Root	MID4	GJHFW2E02IS6OY	Aromatic_amino_acid_degradation	ref|YP_585697.1|	Bacteria	Proteobacteria	Betaproteobacteria	Burkholderiales	Cupriavidus metallidurans CH34	56	5.00E-42	indoleacetamide hydrolase (IAH) (Indole-3-acetamide hydrolase)

iaaH	LN Root	MID4	GJHFW2E02FEOMU	Aromatic_amino_acid_degradation	ref|YP_001020179.1|	Bacteria	Proteobacteria	Betaproteobacteria	Burkholderiales	Methylibium petroleiphilum PM1	46	9.00E-10	indoleacetamide hydrolase gb|ABM93944.1| indoleacetamide hydrolase
iaaH	LN Root	MID5	GJHFW2E02G831Q	Aromatic_amino_acid_degradation	ref|YP_001020179.1|	Bacteria	Proteobacteria	Betaproteobacteria	Burkholderiales	Methylibium petroleiphilum PM1	61	3.00E-21	indoleacetamide hydrolase gb|ABM93944.1| indoleacetamide hydrolase
iaaH	LN Root	MID5	GJHFW2E02FQZNF	Aromatic_amino_acid_degradation	ref|YP_001020179.1|	Bacteria	Proteobacteria	Betaproteobacteria	Burkholderiales	Methylibium petroleiphilum PM1	50	3.00E-28	indoleacetamide hydrolase gb|ABM93944.1| indoleacetamide hydrolase
iaaH	LN Root	MID4	GJHFW2E02JOTQ7	Aromatic_amino_acid_degradation	dbj|BAJ23974.1|	Bacteria				uncultured bacterium	57	4.00E-17	benzamide amidohydrolase
iaaH	LN Root	MID5	GJHFW2E02HWQZM	Aromatic_amino_acid_degradation	gb|EGP48040.1|					Achromobacter xylosoxidans AXX-A	77	6.00E-36	amidase
iaaH	SN Root	MID1	GJHFW2E01BANNM	Aromatic_amino_acid_degradation	ref|YP_001204182.1|	Bacteria	Proteobacteria	Alphaproteobacteria	Rhizobiales	Bradyrhizobium sp. ORS278	80	9.00E-40	Indoleacetamide hydrolase (IAH) (Indole-3-acetamide hydrolase)
iaaH	SN Root	MID1	GJHFW2E01C91EH	Aromatic_amino_acid_degradation	ref|YP_001238174.1|	Bacteria	Proteobacteria	Alphaproteobacteria	Rhizobiales	Bradyrhizobium sp. BTAi1	63	4.00E-20	amidase gb|ABQ34268.1| Indoleacetamide hydrolase
iaaH	SN Root	MID1	GJHFW2E01DFRN0	Aromatic_amino_acid_degradation	ref|NP_766886.1|	Bacteria	Proteobacteria	Alphaproteobacteria	Rhizobiales	Bradyrhizobium japonicum USDA 110	72	5.00E-58	Indole-3-acetamide hydrolase
iaaH	SN Root	MID1	GJHFW2E01EOH7E	Aromatic_amino_acid_degradation	sp|P19922.1|HYIN2_BRAJA	Bacteria	Proteobacteria	Alphaproteobacteria	Rhizobiales	Bradyrhizobium japonicum	88	2.00E-67	Indole-3-acetamide hydrolase
iaaH	SN Root	MID2	GJHFW2E01AWROL	Aromatic_amino_acid_degradation	ref|YP_001203880.1|	Bacteria	Proteobacteria	Alphaproteobacteria	Rhizobiales	Bradyrhizobium sp. ORS278	93	2.00E-67	Indoleacetamide hydrolase (IAH) (Indole-3-acetamide hydrolase)
iaaH	SN Root	MID2	GJHFW2E01CTHF1	Aromatic_amino_acid_degradation	ref|YP_001204182.1|	Bacteria	Proteobacteria	Alphaproteobacteria	Rhizobiales	Bradyrhizobium sp. ORS278	84	9.00E-64	Indoleacetamide hydrolase (IAH) (Indole-3-acetamide hydrolase)
iaaH	SN Root	MID2	GJHFW2E01AFLQ9	Aromatic_amino_acid_degradation	ref|YP_001203880.1|	Bacteria	Proteobacteria	Alphaproteobacteria	Rhizobiales	Bradyrhizobium sp. ORS278	75	1.00E-58	Indoleacetamide hydrolase (IAH) (Indole-3-acetamide hydrolase)
iaaH	SN Root	MID2	GJHFW2E01DMDO2	Aromatic_amino_acid_degradation	ref|NP_766886.1|	Bacteria	Proteobacteria	Alphaproteobacteria	Rhizobiales	Bradyrhizobium japonicum USDA 110	90	1.00E-49	Indole-3-acetamide hydrolase dbj|BAC45511.1| indoleacetamide hydrolase
iaaH	SN Root	MID2	GJHFW2E01DAZ85	Aromatic_amino_acid_degradation	ref|YP_570595.1|	Bacteria	Proteobacteria	Alphaproteobacteria	Rhizobiales	Rhodopseudomonas palustris BisB5	73	1.00E-54	amidase gb|ABE40694.1| Amidase
iaaH	SN Root	MID2	GJHFW2E01EG24V	Aromatic_amino_acid_degradation	ref|YP_001993120.1|	Bacteria	Proteobacteria	Alphaproteobacteria	Rhizobiales	Rhodopseudomonas palustris TIE-1	84	3.00E-61	amidase gb|ACF02645.1| Amidase
iaaH	SN Root	MID2	GJHFW2E01CHGUB	Aromatic_amino_acid_degradation	ref|YP_585697.1|	Bacteria	Proteobacteria	Betaproteobacteria	Burkholderiales	Cupriavidus metallidurans CH34	60	2.00E-30	indoleacetamide hydrolase (IAH) (Indole-3-acetamide hydrolase)
iaaH	SN Root	MID1	GJHFW2E01A6FP3	Aromatic_amino_acid_degradation	dbj|BAJ23974.1|	Bacteria				uncultured bacterium	72	5.00E-14	benzamide amidohydrolase

iaaM	LN Root	MID4	GJHFW2E02GT19J	Aromatic_amino_acid_degradation	ref|NP_421589.1|	Bacteria	Proteobacteria	Alphaproteobacteria	Caulobacterales	Caulobacter crescentus NA1000	60	7.00E-53	amine oxidase
iaaM	LN Root	MID4	GJHFW2E02FQ40Z	Aromatic_amino_acid_degradation	ref|YP_002300075.1|	Bacteria	Proteobacteria	Alphaproteobacteria	Rhodospirillales	Rhodospirillum centenum SW	56	1.00E-48	amine oxidase
iaaM	LN Root	MID4	GJHFW2E02IE65J	Aromatic_amino_acid_degradation	ref|YP_002300075.1|	Bacteria	Proteobacteria	Alphaproteobacteria	Rhodospirillales	Rhodospirillum centenum SW	56	3.00E-49	amine oxidase
iaaM	LN Root	MID4	GJHFW2E02J1RX7	Aromatic_amino_acid_degradation	ref|YP_002300075.1|	Bacteria	Proteobacteria	Alphaproteobacteria	Rhodospirillales	Rhodospirillum centenum SW	59	4.00E-50	amine oxidase
iaaM	LN Root	MID4	GJHFW2E02JWHS6	Aromatic_amino_acid_degradation	ref|YP_002300075.1|	Bacteria	Proteobacteria	Alphaproteobacteria	Rhodospirillales	Rhodospirillum centenum SW	59	6.00E-55	amine oxidase
iaaM	LN Root	MID4	GJHFW2E02G09PE	Aromatic_amino_acid_degradation	ref|YP_001116856.1|	Bacteria	Proteobacteria	Betaproteobacteria	Burkholderiales	Burkholderia vietnamiensis G4	87	2.00E-39	amine oxidase
iaaM	LN Root	MID4	GJHFW2E02I1JDR	Aromatic_amino_acid_degradation	ref|YP_001116856.1|	Bacteria	Proteobacteria	Betaproteobacteria	Burkholderiales	Burkholderia vietnamiensis G4	70	8.00E-33	amine oxidase
iaaM	LN Root	MID4	GJHFW2E02INDZ1	Aromatic_amino_acid_degradation	ref|YP_001116856.1|	Bacteria	Proteobacteria	Betaproteobacteria	Burkholderiales	Burkholderia vietnamiensis G4	83	5.00E-04	amine oxidase
iaaM	LN Root	MID4	GJHFW2E02JCJ8K	Aromatic_amino_acid_degradation	ref|YP_001116856.1|	Bacteria	Proteobacteria	Betaproteobacteria	Burkholderiales	Burkholderia vietnamiensis G4	57	1.00E-10	amine oxidase
iaaM	LN Root	MID4	GJHFW2E02F8T4J	Aromatic_amino_acid_degradation	ref|YP_001116856.1|	Bacteria	Proteobacteria	Betaproteobacteria	Burkholderiales	Burkholderia vietnamiensis G4	85	5.00E-48	amine oxidase
iaaM	LN Root	MID4	GJHFW2E02IU08H	Aromatic_amino_acid_degradation	ref|YP_001116856.1|	Bacteria	Proteobacteria	Betaproteobacteria	Burkholderiales	Burkholderia vietnamiensis G4	91	5.00E-78	amine oxidase
iaaM	LN Root	MID4	GJHFW2E02H02T9	Aromatic_amino_acid_degradation	ref|YP_001116856.1|	Bacteria	Proteobacteria	Betaproteobacteria	Burkholderiales	Burkholderia vietnamiensis G4	69	6.00E-51	amine oxidase
iaaM	LN Root	MID4	GJHFW2E02I7YFI	Aromatic_amino_acid_degradation	ref|YP_001116856.1|	Bacteria	Proteobacteria	Betaproteobacteria	Burkholderiales	Burkholderia vietnamiensis G4	89	1.00E-51	amine oxidase
iaaM	LN Root	MID4	GJHFW2E02IZEFD	Aromatic_amino_acid_degradation	ref|YP_001116856.1|	Bacteria	Proteobacteria	Betaproteobacteria	Burkholderiales	Burkholderia vietnamiensis G4	88	5.00E-71	amine oxidase
iaaM	LN Root	MID4	GJHFW2E02JXMPG	Aromatic_amino_acid_degradation	ref|YP_001116856.1|	Bacteria	Proteobacteria	Betaproteobacteria	Burkholderiales	Burkholderia vietnamiensis G4	87	7.00E-53	amine oxidase
iaaM	LN Root	MID4	GJHFW2E02HO0BK	Aromatic_amino_acid_degradation	ref|YP_001116856.1|	Bacteria	Proteobacteria	Betaproteobacteria	Burkholderiales	Burkholderia vietnamiensis G4	81	1.00E-58	amine oxidase
iaaM	LN Root	MID4	GJHFW2E02ISGL2	Aromatic_amino_acid_degradation	ref|YP_001116856.1|	Bacteria	Proteobacteria	Betaproteobacteria	Burkholderiales	Burkholderia vietnamiensis G4	87	1.00E-55	amine oxidase
iaaM	LN Root	MID4	GJHFW2E02GX2A1	Aromatic_amino_acid_degradation	ref|YP_001116856.1|	Bacteria	Proteobacteria	Betaproteobacteria	Burkholderiales	Burkholderia vietnamiensis G4	88	1.00E-64	amine oxidase
iaaM	LN Root	MID5	GJHFW2E02HWIVF	Aromatic_amino_acid_degradation	ref|YP_001116856.1|	Bacteria	Proteobacteria	Betaproteobacteria	Burkholderiales	Burkholderia vietnamiensis G4	81	6.00E-49	amine oxidase
iaaM	LN Root	MID5	GJHFW2E02IKQZL	Aromatic_amino_acid_degradation	ref|YP_001116856.1|	Bacteria	Proteobacteria	Betaproteobacteria	Burkholderiales	Burkholderia vietnamiensis G4	88	6.00E-33	amine oxidase
iaaM	LN Root	MID6	GJHFW2E02H5GE8	Aromatic_amino_acid_degradation	ref|YP_001116856.1|	Bacteria	Proteobacteria	Betaproteobacteria	Burkholderiales	Burkholderia vietnamiensis G4	75	2.00E-47	amine oxidase
iaaM	LN Root	MID4	GJHFW2E02GD3GX	Aromatic_amino_acid_degradation	ref|YP_995606.1|	Bacteria	Proteobacteria	Betaproteobacteria	Burkholderiales	Verminephrobacter eiseniae EF01-2	72	4.00E-29	amine oxidase
iaaM	LN Root	MID4	GJHFW2E02HAM5M	Aromatic_amino_acid_degradation	ref|YP_995606.1|	Bacteria	Proteobacteria	Betaproteobacteria	Burkholderiales	Verminephrobacter eiseniae EF01-2	60	4.00E-48	amine oxidase
iaaM	LN Root	MID4	GJHFW2E02HD9VO	Aromatic_amino_acid_degradation	ref|YP_995606.1|	Bacteria	Proteobacteria	Betaproteobacteria	Burkholderiales	Verminephrobacter eiseniae EF01-2	46	2.00E-08	amine oxidase
iaaM	LN Root	MID4	GJHFW2E02FD97Z	Aromatic_amino_acid_degradation	ref|XP_003021012.1|	Eukaryota	Ascomycota	Eurotiomycetes	Onygenales	Trichophyton verrucosum HKI 0517	32	5.2	hypothetical protein TRV_04877
iaaM	LN Root	MID4	GJHFW2E02I22GR	Aromatic_amino_acid_degradation	ref|YP_001172137.1|	Bacteria	Proteobacteria	Gammaproteobacteria	Pseudomonadales	Pseudomonas stutzeri A1501	66	2.00E-41	flavin monoamine oxidase-related protein
iaaM	LN Root	MID4	GJHFW2E02I8PE5	Aromatic_amino_acid_degradation	ref|YP_001172137.1|	Bacteria	Proteobacteria	Gammaproteobacteria	Pseudomonadales	Pseudomonas stutzeri A1501	70	1.00E-46	flavin monoamine oxidase-related protein
iaaM	LN Root	MID4	GJHFW2E02IRV98	Aromatic_amino_acid_degradation	ref|YP_001172137.1|	Bacteria	Proteobacteria	Gammaproteobacteria	Pseudomonadales	Pseudomonas stutzeri A1501	72	1.00E-54	flavin monoamine oxidase-related protein
iaaM	LN Root	MID4	GJHFW2E02J1RVT	Aromatic_amino_acid_degradation	ref|ZP_06483703.1|	Bacteria	Proteobacteria	Gammaproteobacteria	Xanthomonadales	Xanthomonas campestris pv. vasculorum NCPPB702	56	5.00E-08	flavin monoamine oxidase-related protein
iaaM	LN Root	MID4	GJHFW2E02JPTUZ	Aromatic_amino_acid_degradation	ref|ZP_06483703.1|	Bacteria	Proteobacteria	Gammaproteobacteria	Xanthomonadales	Xanthomonas campestris pv. vasculorum NCPPB702	49	4.00E-17	flavin monoamine oxidase-related protein
iaaM	LN Root	MID4	GJHFW2E02I4EPU	Aromatic_amino_acid_degradation	ref|ZP_08180487.1|	Bacteria	Proteobacteria	Gammaproteobacteria	Xanthomonadales	Xanthomonas vesicatoria ATCC 35937	56	3.00E-34	monoamine oxidase
iaaM	SN Root	MID2	GJHFW2E01DNJZ5	Aromatic_amino_acid_degradation	ref|YP_096846.1|	Bacteria	Proteobacteria	Gammaproteobacteria	Legionellales	Legionella pneumophila Philadelphia 1	46	1.00E-10	protoporphyrinogen oxidase

ipdC	LN Root	MID4	GJHFW2E02FZUJU	Auxin_biosynthesis	ref|ZP_08074854.1|	Bacteria	Proteobacteria	Alphaproteobacteria	Rhizobiales	Methylocystis sp. ATCC 49242	68	5.00E-38	thiamine pyrophosphate TPP-binding domain-containing protein
ipdC	SN Root	MID2	GJHFW2E01BLFCB	Auxin_biosynthesis	ref|ZP_01091097.1|	Bacteria	Planctomycetes	Planctomycetacia	Planctomycetales	Blastopirellula marina DSM 3645	71	2.00E-39	indole-3-pyruvate decarboxylase
ipdC	SN Root	MID1	GJHFW2E01DCFYY	Auxin_biosynthesis	ref|YP_003628930.1|	Bacteria	Planctomycetes	Planctomycetacia	Planctomycetales	Planctomyces limnophilus DSM 3776	51	6.00E-22	thiamine pyrophosphate protein TPP binding domain protein

Table S3. Statistics for the 16S rRNA gene pyrosequence of rice root-associated and paddy soil bacteria under low (LN) and standard (SN) N fertilization*

Sample IDs†	No. of reads	99% similarity				97% similarity				95% similarity			
		OTUs	Coverage‡	Chao1	Shannon	OTUs	Coverage	Chao1	Shannon	OTUs	Coverage	Chao1	Shannon
Soil LN1	7,838	3392	0.72	7827	7.78	2424	0.84	4235	7.28	1929	0.88	3293	6.89
Soil LN2	10,380	5082	0.66	12810	8.30	3831	0.78	7835	7.80	3078	0.84	5942	7.40
Soil LN3	9,671	4536	0.69	11403	8.14	3384	0.80	6607	7.66	2676	0.86	4842	7.25
Soil LN total	27,889	10206	0.77	23782	8.57	6829	0.87	12955	7.97	5178	0.91	9133	7.51
Soil SN1	11,441	5225	0.71	11312	8.35	4078	0.81	7404	7.95	3311	0.86	5629	7.60
Soil SN2	5,897	2880	0.69	6375	7.86	2358	0.77	4557	7.50	1963	0.83	3443	7.21
Soil SN3	10,076	4382	0.73	9218	8.13	3322	0.83	5752	7.71	2630	0.88	4283	7.35
Soil SN total	27,414	9826	0.79	19832	8.69	6880	0.88	11842	8.19	5260	0.92	8476	7.77
Root LN1	5,371	895	0.90	1819	4.98	616	0.94	1031	4.57	487	0.96	725	4.34
Root LN2	4,516	805	0.89	1753	4.86	568	0.93	1047	4.47	454	0.95	780	4.22
Root LN3	41,978	3729	0.95	7202	4.73	1966	0.98	2986	4.22	1413	0.99	2006	3.96
Root LN total	51,865	4437	0.95	8475	4.87	2279	0.98	3541	4.34	1617	0.99	2344	4.07
Root SN1	35,744	6113	0.90	11934	6.91	3513	0.96	5545	6.31	2528	0.97	3652	5.90
Root SN2	31,018	5216	0.90	10336	6.64	2962	0.96	4386	6.06	2153	0.97	2910	5.63
Root SN3	20,789	3793	0.90	7505	6.54	2235	0.96	3405	5.99	1610	0.97	2266	5.55
Root SN total	87,551	11221	0.93	21964	6.98	5457	0.97	8335	6.29	3680	0.99	5077	5.82

* OTUs were defined at 99%, 97%, and 95% sequence similarity. DNAs were prepared from three independent samples for each treatment.

† Barcode (MID) pyrosequences were conducted using three independent samples for each treatment. Gray shading indicates the subtotal of each treatment. Each DNA sample was independently analyzed from field sampling (a composite sample) to pyrosequenced PCR products of 16S rRNA genes.

‡ $C_x = 1 - (n_x/N)$, where n is the number of singletons encountered only once in the library and N is the total number of reads in the library.

Table S4. Soil chemical propeties of paddy field soils under low N (LN) and standard N (SN) fertilization condition[a]

Soil property	LN soil			SN soil			*P* (*t*-test)
	Mean		SD	Mean		SD	
pH (H_2O)	5.42	±	0.03	5.25	±	0.20	0.279
Total N (%)	0.17	±	0.01	0.15	±	0.01	0.079
NH_4-N (mgN/100 g)	5.01	±	0.16	4.16	±	0.20	0.005
NO_3-N (mgN/100 g)	0.46	±	0.26	0.48	±	0.24	0.951
Organic C (%)	3.11	±	0.03	2.81	±	0.06	0.004
CEC (meq/100 g)	29.87	±	0.70	23.55	±	0.53	0.000

[a] Gray indicates significant difference between LN and SN soils based on *t*-test ($P<0.01$). SD shows standard deviation of triplicate determinations.
